# Winter Activity of Coastal Plain Populations of Bat Species Affected by White-Nose Syndrome and Wind Energy Facilities

**DOI:** 10.1371/journal.pone.0166512

**Published:** 2016-11-16

**Authors:** John F. Grider, Angela L. Larsen, Jessica A. Homyack, Matina C. Kalcounis-Rueppell

**Affiliations:** 1 Warnell School of Forestry and Natural Resources, University of Georgia, Athens, Georgia, United States of America, 30602; 2 Biology Department, University of North Carolina at Greensboro, Greensboro, North Carolina, United States of America, 27402; 3 Weyerhaeuser Company, 505 North Pearl Street, Centralia, Washington, United States of America, 98531; University of Porto, PORTUGAL

## Abstract

Across the entire distribution of a species, populations may have variable responses to environmental perturbations. Many bat species experience mortality in large portions of their range during hibernation and along migratory paths to and from wintering grounds, from White-nose syndrome (WNS) and wind energy development, respectively. In some areas, warm temperatures may allow bats to remain active through winter, thus decreasing their susceptibility to WNS and/or mortality associated with migration to wintering grounds. These areas could act as a refugia and be important for the persistence of local populations. To determine if warmer temperatures affect bat activity, we compared year-round activity of bat populations in the Coastal Plain and Piedmont of North Carolina, USA, two regions that differ in winter temperature. We established six recording stations, four along a 295-kilometer north-south transect in the Coastal Plain, and two in the Piedmont of North Carolina. We recorded bat activity over two years. We supplemented our recordings with mist-net data. Although bat activity was lower during winter at all sites, the odds of recording a bat during winter were higher at Coastal Plain sites when compared with Piedmont sites. Further, bats in the Piedmont had a lower level of winter activity compared to summer activity than bats in the Coastal Plain that had more similar levels of activity in the winter and summer. We found high bat species richness on the Coastal Plain in winter, with winter-active species including those known to hibernate throughout most of their range and others known to be long distance migrants. In particular, two species impacted by WNS, the northern long-eared bat (*Myotis septentrionalis*) and tricolored bat (*Perimyotis subflavus*), were present year round in the Coastal Plain. The tricolored bat was also present year-round in the Piedmont. In the Coastal Plain, the long distance migratory hoary bat (*Lasiurus cinereus*) was active in the winter but not present during the other seasons, and the long distance migratory silver-haired bat (*Lasionycteris noctivagans*) was active primarily in the winter, suggesting the Coastal Plain may be an overwintering ground for these two species. We suggest that the winter activity exhibited by populations of bats on the North Carolina Coastal Plain has important conservation implications and these populations should be carefully monitored and afforded protection.

## Introduction

In temperate regions during the winter, bats generally migrate, either to hibernacula or warmer wintering grounds. Bats that hibernate in winter when food becomes scarce often undertake regional migrations up to several hundred kilometers [1]. Alternatively, some temperate bat species such as *Lasiurus cinereus* and *Lasionycteris noctivagans*, migrate long distances to warmer wintering grounds [1,2]. During these movements, bats use distinct geographic features for navigation, and forested areas for brief stopovers to forage and roost [3–5]. Although rare, some individuals do not leave their summering area, but instead remain resident and active, or remain resident and use a combination of activity and short torpor bouts [6,7]. This winter residency is possible where nightly temperatures are warm enough for insectivorous prey to be active.

Currently, seven temperate bat species in eastern North America have shown symptoms of White-nose syndrome (WNS) with some species experiencing high mortality during hibernation [8]. WNS is caused by *Pseudogymnoascus destructans*, a fungus that grows on the skin of bats during hibernation [9,10]. While death from WNS is not fully understood, the disease causes more frequent arousal events during winter torpor bouts, leading to death [11,12]. WNS has killed millions of bats in the United States [13], with several species seeing significant declines [14]. Bats that do not succumb to WNS in the winter months often show signs of deteriorated wings and poor body condition, which can lower future foraging and reproductive success [15].

Bats have also seen increased mortality associated with wind turbines. Bat fatalities from wind turbines occur predominantly in *Lasiurus cinereus*, *Lasiurus borealis*, and *Lasionycteris noctivagans* in late summer and early autumn, coinciding with their seasonal migration [1,16,17]. Arnett and Baerwald [18] estimated that hundreds of thousands to > 1 million bats died from wind turbines between 2000 and 2011. Hypotheses about why bats are killed at wind turbines center on pre-existing sensory biases that make turbines attractive to bats [16]. Bats may not have the cognitive ability to distinguish turbines from trees, and may approach turbines expecting to land at potential roost sites, find insects aggregations on the leeward side, or find other bats as potential mates [16,17]. Regardless of why bats are attracted to wind turbines, fatalities are increased because wind turbines are often placed adjacent to migratory corridors, such as forested ridgetops [3]. Thus, bats that make seasonal movements associated with the onset and retreat of winter are more susceptible to mortality from wind turbines than resident bats.

Semi-tropical and temperate coastal areas, like those of the Coastal Plain of the southeastern United States, may be warm enough for bats to remain active year-round. These areas have mild winters due to pole-ward movement of ocean waters that release heat to surrounding land masses as they move from tropical regions, a process of land warming that has more influence on temperature during winter months [19,20]. Six of the species that occur in the North Carolina Coastal Plain have experienced mortality over some of their range due to WNS and wind turbines. If populations of these species use different behaviors and are active in the Coastal Plain year round, they could avoid contact with, or have reduced mortality from WNS. If the tree bat species do not migrate, then they may also have lower fatalities from wind turbines. The objective of this study was to determine if populations of bats in the North Carolina Coastal Plain sustain higher winter activity than non-coastal populations.

## Methods

**Fig 1:**
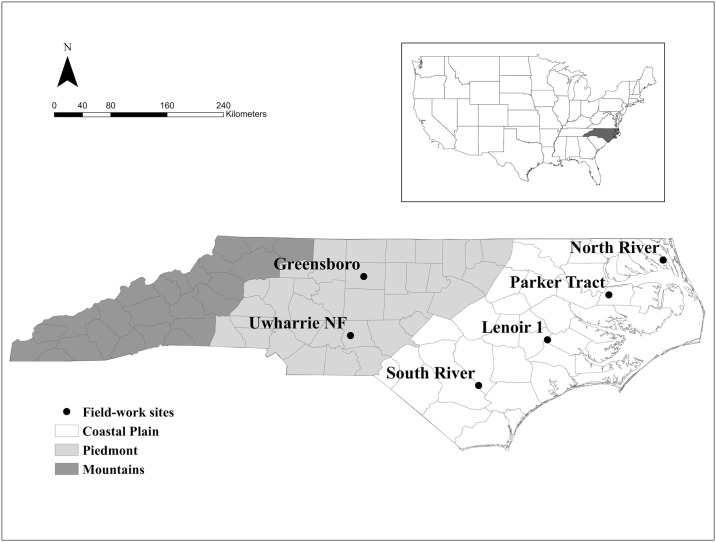
Location of field sites in the Piedmont and Coastal Plain regions of North Carolina, USA. The two Piedmont sites are Greensboro and the Uwharrie National Forest. The four Coastal Plain sites are North River, Parker Tract, Lenoir 1, and South River. Despite the proximity of regions, winters are milder in the Coastal Plain than the Piedmont.

We examined bat activity at six sites in the Coastal Plain and Piedmont of North Carolina (Fig 1). Sites represented common forest types of each region. The Coastal Plain sites included two intensively managed forest (Parker Tract and Lenoir 1), and two bottomland hardwood forest (North River Game Land, and South River) sites. The South River site was originally located on Whitehall Plantation Game Land (near South River) but equipment was vandalized and subsequently moved to South River in February 2013. Sites in the Piedmont contained an urban eastern mixed hardwood forest (Greensboro) and an eastern mixed hardwood forest (Uwharrie National Forest).

### Field Methods

Bat activity was measured using Song Meter SM2 and SM2+ bat ultrasound detectors (henceforth “detectors”; Wildlife Acoustics, Concord, Massachusetts), which recorded sunset to sunrise, each night. Microphones were set 10 to 30 feet off the ground. For recording consistency among detectors, we used the following settings: recording at 48 decibels, high-pass filter set to 1000 Hertz, and sampling rate set to 192,000 Hz (the only option for the SM2+).

Acoustic data were collected on 2,885 nights from 1 September 2012 to 31 August 2014. Stations operated for an average of 480.8 (SD 137.2) nights during the study. Recording did not take place every night due to equipment malfunction, theft, and wildlife encounters. During the winter of 2012, recording stations were missing the first hour of recording for three days at three coastal sites due to a mistake in settings on the recording units. These nine days were included, even though they underestimated winter activity.

We measured relative bat activity by manually examining and counting all recorded .wav files in SonoBat 3.2 NE (henceforth “SonoBat”; DND Designs, Arcata, California). Files containing at least one bat echolocation pulse were counted as a single echolocation sequence (henceforth sequence) for the night it was recorded. Files with recordings from more than one bat were conservatively counted as a single sequence. Files that contained bat social calls (ie, calls not part of an echolocation sequence) were counted as a sequence if a search phase echolocation pulse was in the recording. Eleven winter nights with high numbers of sequences were randomly selected to determine if they contained feeding buzzes [21,22]

Sequences were analyzed to species using the auto classifiers SonoBat 3.2 NE and BCID East 2.6a (henceforth “BCID”; Bat Call Identification Inc., Kansas City, Missouri). SonoBat contains all species potentially present, except for the Southeastern myotis (*M*. *austroriparius*), Seminole bat (*Lasiurus seminolus*) and Northern yellow bat (*Lasiurus intermedius*). BCID contains all species potentially present with the exception of the Seminole bat and the Northern yellow bat. For auto classification, files were first processed through the SonoBat SM2 Batch Attributer and Batch Scrubber 5.2 to compensate for using xms-ultrasound microphones and to remove low quality recordings. Second, remaining files were run through SonoBat using recommended settings for SM2 and SM2+ recordings as maximum number of calls to consider per file = 8, acceptable call quality = 0.7, and decision threshold = 0.9. Further, identified echolocation sequences were not accepted unless a minimum of 3 pulses was identified and there was “consensus” species decision. All sequences identified using SonoBat were then converted to zero-cross files using Kaleidoscope software 2.0.7 (Wildlife Acoustics, Concord, Massachusetts). Converted zero-cross files were then identified a second time using BCID, to validate the initial classification made in SonoBat. In BCID the default settings were used, and at least five identifiable pulses were needed to identify a species. All identified calls from the genus *Myotis* were manually inspected to confirm identification and were put into one group, *Myotis*. Identifications were only accepted and used for analysis if there was concordance between SonoBat and BCID.

In total, 152,078 echolocation sequences were recorded. On average 25,346.3 (SD 23,466.9) sequences were recorded at each site, and across all sites an average of 3,543.2 (SD 5,923.3) sequences were recorded during each season. Of the 152,078 sequences recorded, 36,632 could be identified to species using SonoBat and of these, 7,238 could be identified to the same species using BCID. Although 7,238 recorded files could be identified to species (S1 Table), making inferences about seasonal changes in species composition was limited by small sample sizes at some sites. Thus, we examined species presence based on acoustic recordings by region.

Both relative bat activity and species-specific relative bat activity were examined in relation to nightly temperature and season to assess differences in winter activity between Piedmont and Coastal Plain populations. We defined winter as December–February, spring as March–May, summer as June–August, and autumn as September–November. Nightly temperature was determined for all recording nights by calculating a mean of all hourly temperature measurements between sunset and sunrise. Hourly temperature measurements were obtained from weather stations run by the North Carolina State Climate office (http://www.nc-climate.ncsu.edu). Weather stations were 12.3 km (SD 6.1) with a range of 4.2 to 22.9 km, from recording sites. Nights with missing temperature data were only used if two or fewer non-consecutive, hourly temperature measurements were missing. Our seasonal definitions corresponded to warmest and coldest months, for summer and winter, respectively, based on actual temperatures during this study.

Mist netting for bats in the Coastal Plain occurred on 61 nights between summer 2012 and winter 2013 with the majority of effort occurring in summer 2012 (S2 Table). We used mist netting to complement bat activity data and to confirm the presence of species detected through recordings. Mist netting occurred at all Coastal Plain sites during summer 2012 and sporadically in the spring and winter of 2013. Mist-nets were set up on road/forest corridors or around bodies of water using standard mist netting techniques [23] and under the permission of the North Carolina Wildlife Resources Commission and the UNCG Institutional Animal Care and Use Committee.

### Statistical Methods

Normality and equality of variance of activity data were tested using Shapiro-Wilk and Levene’s tests, respectively. Data violating parametric assumptions were normalized using natural log transformations. When transformations failed to normalize data, non-parametric tests were used. Because of missing activity data during parts of some seasons in some years, year could not be used as a unit of replication. Instead, seasons were pooled across years (i.e., a summer night in 2012 and 2013 was coded as “summer”).

Kruskal-Wallis tests were used to compare winter temperatures between the Piedmont and Coastal Plain regions. A ratio of the sum of summer sequences to winter sequences was calculated for each site to determine likelihood of recording summer *vs* winter echolocation pulse sequences. Seasons did not have the same number of recording nights, therefore, all ratio numerators and denominators were constrained to the smaller number of sampling nights by averaging the sum of 1000 random subsets of nights from the season with the larger number of sampling nights. We only had 2 Piedmont sites; therefore we did not statistically compare ratios, but describe magnitude of difference of ratios between selected sites in each region. A generalized linear mixed effects model was used to analyze the effects of temperature (Celsius), region (Piedmont and Coastal Plain), season (winter and summer), and site (as a random effect) on bat activity (calls/night). The model was run on untransformed data [24]. Because our sample consists of count data, our sample size is large, and our data are robust to interpretation issues from transformations, a generalized mixed effect model with a negative binomial distribution (log link) and zero-inflation was used to account for overdispersion and excess zeros, respectively. Residual and qq plots were used to determine that the negative binomial distribution was the best fit for the data. Rather than using an automated selection process, only biologically relevant models were considered. Model selection was based on Akaike information criterion (AIC) values and the Akaike’s weights of the limited model set. Models were further validated with Markov Chain Monte Carlo methods [25,26]. Although not all main effects were statistically significant, all remained in the model to include interactions. Program R 3.3.0 [27] was used for all statistical analyses [packages: glmmADMB [25,26], and pgirmess [28]].

## Results

Coastal Plain sites were warmer on winter nights than Piedmont sites (Kruskal-Wallis chi-squared = 24.33, df = 1, p < 0.001). The average nightly winter temperature at Piedmont sites was 4.1 (SD 5.7) °C (n = 358 nights) whereas the average nightly winter temperature at Coastal Plain sites was 6.0 (SD 5.9) °C (n = 689 nights).

Ratios of summer to winter sequences (Table 1) were highest in the two Piedmont sites and Lenoir 1, a Coastal Plain site. Except for Lenoir 1, ratios were an order of magnitude less, and closer to 1:1 in the Coastal Plain when compared to the Piedmont (Table 1). For example, in Greensboro there were 37.8 times more summer bat echolocation pulse sequences recorded than winter bat echolocation pulse sequences, whereas those values in Parker Tract and South River were only 1.2 and 2.5, respectively (Table 1). The highest Piedmont ratio (Greensboro) was 31.5 times that of the lowest Coastal Plain ratio (Parker Tract) and the lowest Piedmont ratio (Uwharrie National Forest) was still 1.8 times that of the highest Coastal Plain ratio (Lenoir 1; Table 1).

**Table 1:** Average (± 1SE) number of echolocation pulse sequences at each site per night, and ratios of the sum of summer echolocation pulse sequences divided by the sum of winter echolocation pulse sequences.

Site	Region	Summer	Winter	Adjusted Ratio
Greensboro	Piedmont	25.4±1.9	0.7±0.1	37.8
Uwharrie	Piedmont	529.4±38.6	21.5±9.0	24.7
North River	Coastal Plain	85.7±6.6	11.2±2.3	7.6
Parker Tract	Coastal Plain	10.4±0.6	8.9±2.6	1.2
Lenoir 1	Coastal Plain	10.2±2.1	0.8±0.4	13.5
South River	Coastal Plain	110.6±13.3	56.2±15.1	2.5

Ratios were adjusted to account for differences in the number of days sampled between seasons.

Our top generalized linear mixed effects model assessing fixed effects of temperature, region, and season, with site as a random effect on bat activity had a three-way interaction of temperature, season, and region (Tables 2 and 3).

**Table 2:** Top models investigated to explain variation in calls per night with number of parameters per model (k).

Model	k	*w*_*i*_	Δ AIC
**Activity ~ {t+r+s+t:r+r:s+t:r:s+(t:s|site)}**	**13**	**0.707**	**0.0**
Activity ~ {t+s+(t:s|site)}	8	0.293	1.8
Activity ~ {t+r+s+t:r+r:s+t:s+t:r:s+(1|site)}	11	0.000	129.5
Activity ~ {r+t:r+(t|site)}	8	0.000	149.8
Activity ~ {1+(1|site)}	4	0.000	1162.0

The best model (bold) was selected based on AIC and normalized Akaike weight (*w*_*i*_). Temperature (t), region (r), and season (s) were fixed effects and site was a random effect. For model syntax, we used a (:) to denote an interaction and (|) to denote a random effect with effect|grouping factor; see [29] for more detailed syntax description.

**Table 3:** Coefficient estimates from the best fit model using reference groups region = Piedmont and season = summer.

	Estimate	SE	z	P value
Intercept_Piedmont and Summer_	2.89	1.03	2.79	0.01
Temperature	0.09	0.06	1.58	0.11
Region_Coastal Plain_	-0.27	1.21	-0.22	0.82
Season_Winter_	-4.15	0.88	-4.71	<0.01
Temperature: Region_Coastal Plain_	-0.05	0.07	-0.76	0.45
Region_Coastal Plain_: Season_Winter_	0.29	1.02	0.29	0.77
Temperature: Region_Piedmont_: Season_Winter_	0.23	0.09	2.42	0.02
Temperature: Region_Coastal Plain_: Season_Winter_	0.25	0.06	3.89	<0.01

For all re-leveling see S3 Table. Data were collected in the Coastal Plain and Piedmont regions of North Carolina from 1 September 2012 to 31 August 2014. Best fit model: Activity ~ Temperature+Region+Season+Temperature:Region+Region:Season+Temperature:Region:Season+ (Temperature:Season | Site).

**Fig 2:**
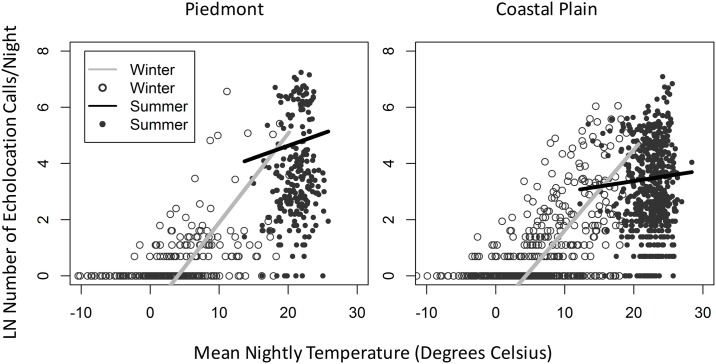
Scatterplots by region of the natural log number of echolocation pulses recorded per night by average nightly temperature, in both summer and winter. Lines were fit based on the best fit model: Activity ~ Temperature+Region+Season+Temperature:Region+Region:Season+Temperature:Region:Season+(Temperature:Season | Site). Predicted bat activity values were derived on the log link scale. The difference in bat activity between summer and winter is greater in the Piedmont than the Coastal Plain. The positive relationship between temperature and activity is similar between Piedmont and Coastal Plain in the winter, but differs in the summer with a more positive relationship in the Piedmont than the Coastal Plain.

Bat activity responded positively to temperature regardless of region (Fig 2). Given the same increase in temperature, bats respond with higher activity in winter than summer (Fig 2). Activity during summer and winter was dependent on region, with Piedmont bats showing a lower level of winter activity compared to summer activity and Coastal bats showing more similar levels of activity in the winter and summer (Fig 2). The relationship between temperature and activity was different in the summer between regions with bat activity in the Piedmont being more positively related to temperature than bat activity in the Coastal Plain (Fig 2).

An analysis of 11 randomly selected subset of nights with high winter activity revealed that bats were feeding. The percentage of calls with feeding buzzes in the Coastal Plain was 13.4 (SD 5.7) % (range = 5.5–21.1%, n = 6 nights) and in the Piedmont was 12.9 (SD 10.9) % (range = 2.4–28.5%, n = 5 nights). Therefore, the bats that were active during the winter in both the Coastal Plain and the Piedmont were feeding.

**Fig 3:**
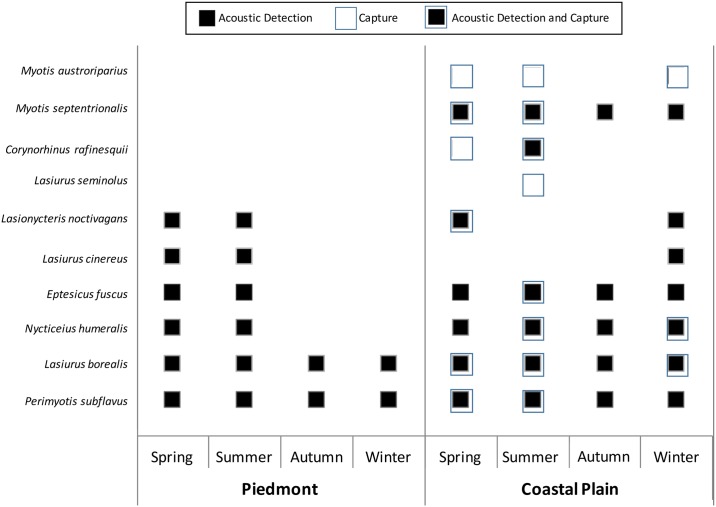
Species presence by season and region. Presence was determined from an acoustic detection, mist-net capture, or both. In the Piedmont, species richness was high in the spring and summer, but not the fall and winter. In contrast, species richness was high in all seasons in the Coastal Plain.

Species richness was high (6 species) in the spring and summer, but low (2 species) in the autumn and winter in the Piedmont (Fig 3). Winter was the season with the highest richness in the Coastal Plain (7 species). In both regions, *L*. *borealis* and *Perimyotis subflavus* were recorded year round. Additionally, *Eptesicus fuscus*, *Nycticeius humeralis*, and *M*. *septentrionalis* were recorded year round in the Coastal Plain. The migratory tree bats, *L*. *cinereus* and *L*. *noctivagans*, were only detected in the spring and summer in the Piedmont, but were present during the winter in Coastal Plain. Furthermore, *L*. *cinereus* and *L*. *noctivagans* were the only species recorded at Parker Tract in winter and they were not recorded during any time other than the winter at Parker Tract and South River (S1 Table), suggesting the Coastal Plain is a wintering site for these two species. During spring in the Piedmont (specifically the Uwharrie National Forest–S1 Table), six species were present including *L*. *cinereus*, *L*. *noctivagans*, *N*. *humeralis*, *E*. *fuscus*, *L*. *borealis*, and *P*. *subflavus*, suggesting *L*. *cinereus* and *L*. *noctivagans* were spring migrants either into or through the area.

Supplementing acoustic data with mist netting data from the Coastal Plain supported seasonal acoustic results (Fig 3). Both *M*. *austroriparius* and *M*. *septentrionalis* were present in the winter, along with the migratory tree bat species, *L*. *cinereus* and *L*. *noctivagans*, and the more common species that included *N*. *humeralis*, *E*. *fuscus*, *L*. *borealis*, and *P*. *subflavus*. We captured *Corynorhinus rafinesquii* in spring and summer.

## Discussion

We found that although bat activity was lower during winter than summer at all sites, bats in the Piedmont had a lower level of winter activity compared to summer activity than bats in Coastal Plain that had more similar levels of activity in summer and winter. We found high species richness on the Coastal Plain in the winter, including species known to hibernate throughout most of their distribution, and others known to be long distance migrants. Long distance migratory bats used the Coastal Plain and Piedmont regions differently. *L*. *cinereus* and *L*. *noctivagans* appeared to use the Piedmont for migration and the Coastal Plain as a wintering ground, whereas *L*. *borealis* appeared to be resident, year round, in both regions. Thus, our results show that bats on the Coastal Plain have a unique winter biology that is important for multiple species’ conservation in the face of winter mortality associated with WNS and collisions with wind turbines during migration. For example, on the Coastal Plain, resident and winter-active species such as *M*. *septentrionalis*, *E*. *fuscus*, and *P*. *subflavus* potentially avoid mortality due to WNS and *L*. *borealis* potentially avoids mortality due to wind-turbine collisions during migration. Therefore, we suggest careful monitoring and protection of Coastal Plain populations.

For a bat to remain resident in an area over winter, temperatures must be warm enough for bats and their insect prey to stay active. Although the average nightly temperature was only ~ 1.5°C higher on the Coastal Plain, it was near the lower temperature limit for flying insects, e.g., [30]. The Coastal Plain may offer more opportunities for winter foraging activity by bats and our bat activity, feeding buzz, and richness results suggest that small differences in average nightly temperature can influence the winter biology of bats. Our study confirmed that temperature positively influences bat activity [7,31–33]; however, this positive relationship did not explain all the variation seen in regions and seasons, especially in the summer. Potential abiotic mediators may be humidity, air pressure, and precipitation.

A comparable level of summer and winter bat activity in the Coastal Plain contradicts typical behavior of temperate bat species which hibernate or migrate during colder parts of the year [2,34]. Not hibernating could mean lower reproductive success for some species since hibernacula are known as important sites for mating of many temperate bat species. Alternatively, bats on the Coastal Plain may not rely on autumn mating swarms for mating, but instead may mate during other times of the year. There is evidence that bats in warmer temperate areas do not copulate until the spring [6]. Regardless, bats that forgo migration to stay resident and active on the Coastal Plain could see reduced mortality from the physiological stresses associated with migration and hibernation in other parts of their range [1].

We saw consistent patterns between seasons, with winter having overall lower activity levels than summer, but with the difference between summer and winter activity levels being less in the Coastal Plain than in the Piedmont. Low levels of winter activity have been found in other studies of bats [33,35]. The odds of recording an echolocation sequence in the winter, compared to the summer, in the North Carolina Coastal Plain was much higher than in the Piedmont and this was true for every Coastal Plain site. For example, activity was almost 38 times higher in summer than winter at Greensboro (Piedmont), while activity between summer and winter at Parker Tract (Coastal Plain) was nearly equal. Importantly, there was still feeding activity at all sites, including Piedmont sites, during the winter and our findings for winter behavior, even at Piedmont sites with low winter activity relative to summer activity, are consistent with previous reports of winter feeding by bats [6,7,36], including a study from the Coastal Plain of North Carolina and Virginia that showed *L*. *borealis* was able to forage during winter [37].

Ratios of summer to winter activity also showed site-specific variability in bat activity. On the Coastal Plain, managed pine forest sites (Parker Tract and Lenoir 1) had lower activity than bottomland hardwood sites. Managed pine forests likely had lower activity because bats favor vertical structure, tree species richness, and large roost trees in open areas which are not commonly found in managed timber lands [38,39]. Lower activity at Lenoir 1 may have also been due to microphone placement in the interior of an unmanaged pine stand where activity is generally lower than on the edge of stands [40]. The Uwharrie National Forest, in the Piedmont, had the highest level of activity out of all six sites likely due to the recording station being located near a bright light that illuminated the site at night. Light sources are known to attract insects at night and this can influence bat activity [41]. Site level differences were not known *a priori*, and are common in studies of bats [31,42]. Because of the inherent differences in sites, site was included as a random effect in the generalized mixed effect models. That allowed for focus on effects of temperature, region, and season.

We were conservative with species identification to ensure calls were identified correctly and are confident about species presence based on acoustic sampling. In the Coastal Plain, year round residents included *M*. *septentrionalis*, *L*. *borealis*, *N*. *humeralis*, and *P*. *subflavus*, whereas year round residents in the Piedmont included *L*. *borealis* and *P*. *subflavus*. There are three species of bats associated with long distance migration that occur in the study area, *L*. *cinereus*, *L*. *noctivagans*, and *L*. *borealis* [1,2]. Of these, *L*. *borealis* was the most common species captured or recorded during each season suggesting that it does not migrate in either the Piedmont or the Coastal Plain. On the other hand, *L*. *cinereus* and *L*. *noctivagans* were only detected intermittently on the Coastal Plain and occurred during different times of the year in the Piedmont and Coastal Plain. *L*. *cinereus* and *L*. *noctivagans* were present almost exclusively (except for 1 recording of L. noctivagans in the spring; S1 Table) during the winter on the Coastal Plain, whereas they were present in the Piedmont during spring and summer and never detected in the Piedmont during the autumn. In particular, in the Piedmont, *L*. *cinereus* had high activity during the spring. Our results suggest that *L*. *cinereus* and *L*. *noctivagans* used Piedmont sites as stopover points along spring migratory routes whereas the Coastal Plain was used as a wintering ground. Previous studies have shown that stopover points are commonly used during bat migration and can be important sites for bats to rest along their migratory pathway [5,43]. Thus, our results suggest bats may use different migratory routes across seasons.

### Wind Turbines

Long distance migratory bat species face a growing threat from encounters with wind turbines along their migratory corridors [17,44]. Currently there are a few smaller wind turbines in the Coastal Plain of North Carolina with most of them on the Outer Banks [45]; however, if a bat is remaining resident it is less likely to experience mortality from wind turbines since mortality primarily occurs during migration. Furthermore, the prime areas for wind energy in the Coastal Plain are offshore and not likely to affect resident bats if ever constructed [46]. Year round activity of *L*. *borealis* on the Coastal Plain suggests that some individuals are not migrating and may not experience mortality from wind facilities. Other long distance migratory bats however, such as *L*. *noctivagans* and *L*. *cinereus*, were never detected in summer but were detected in the winter suggesting that these species are making seasonal migrations which may put them at risk for mortality associated with wind facilities [44].

### WNS

Throughout most of their range, *M*. *septentrionalis* and *P*. *subflavus* are known to make seasonal movements to caves for hibernation [47,48], where there is high mortality from WNS. In contrast, our study shows that these species can remain active year round on the Coastal Plain of North Carolina, where there are no known hibernacula. Recent mist-netting efforts have confirmed our conclusion that *M*. *septentrionalis* is present year round in the Coastal Plain as individuals have been captured during the 2015/2016 winter season (K. Caldwell, North Carolina Wildlife Resources Commission, personal communication). While it is currently unknown whether or not WNS is present on the Coastal Plain, it is not predicted to reach the region until after the year 2050, largely due to its isolation from known areas of occurrence [49]. Thus, individuals remaining resident on the Coastal Plain could suffer less mortality from WNS. However, the viability of persistence and number of individuals within these potential refugial populations is unknown.

Previous research showed that bats displayed different behavior throughout their range [6,50]. This study provides further evidence that populations of bats in the Coastal Plain of North Carolina sustain more consistent year round activity than inland populations. These populations’ ability to sustain higher activity throughout winter could result in less mortality associated with WNS [9] and anthropogenic factors, such as wind facilities found in other parts of the species range [17]. These factors could ultimately lead to populations of bats in the Coastal Plain becoming source or rescue populations for re-colonization of locally extinct or depleted populations. Results from this study suggest that these refugial populations could be valuable for the conservation efforts of some bat species. However, without knowledge on the number and age structure of individuals in these populations, their long term viability is uncertain [51]. Abundance has been shown to be a key factor in the persistence of populations, and if coastal populations are too small or rely on individuals dispersing from the other areas, they will likely not persist [52,53].

## Supporting Information

S1 Table Recordings identified to species through automated acoustic ID programs at each site by season in 2012 and 2013. Years are pooled. Numbers indicate how many files were identified for a given species during a particular season. Seasons are abbreviated S (spring), M (summer), A (autumn), and W (winter). Piedmont sites are Greensboro and the Uwharrie National Forest. Coastal Plain sites are North River, Parker Tract, Lenoir 1, and South River.
(DOCX)

S2 Table Capture data from mist-netting in 2012 and 2013 on the Coastal Plain of North Carolina. The numbers in brackets represent the number of female and male bats captures (female, male). In some cases where the bat escaped before gender could be determined, the number of males and females will not add up to the total.
(DOCX)

S3 Table Coefficient estimates from the best fit model re-leveling the reference groups of region and season. Data were collected in the Coastal Plain and Piedmont regions of North Carolina from 1 September 2012 to 31 August 2014. Best fit model: Activity ~ Temperature * Region * Season − Temperature: Season + (Temperature: Season | Site).
(DOCX)

S4 Table Bat activity and temperature data. (PDF)

S5 Table Winter temperature data. (PDF)
