# Supplementary material for: Winter Activity of Coastal Plain Populations of Bat Species Affected by White-Nose Syndrome and Wind Energy Facilities
Source: PLoS One. 2016 Nov 16;11(11):e0166512. doi: 10.1371/journal.pone.0166512 (PMC5112809; doi:10.1371/journal.pone.0166512)
Supplement: S1 Table — Years are pooled. Numbers indicate how many files were identified for a given species during a particular season. Seasons are abbreviated S (spring), M (summer), A (autumn), and W (winter). Piedmont sites are Greensboro and the Uwharrie National Forest. Coastal Plain sites are North River, Parker Tract, Lenoir 1, and South River. (DOCX) [file pone.0166512.s001.docx]

**S1 Table. Recordings identified to species through automated acoustic ID programs at each site by season in 2012 and 2013.** Years are pooled. Numbers indicate how many files were identified for a given species during a particular season. Seasons are abbreviated S (spring), M (summer), A (autumn), and W (winter). Piedmont sites are Greensboro and the Uwharrie National Forest. Coastal Plain sites are North River, Parker Tract, Lenoir 1, and South River.

| **Site** | ***Perimyotis subflavus*** | | ***Lasiurus borealis*** | | ***Nycticeius humeralis*** | | ***Eptesicus fuscus*** | | ***Lasiurus cinereus*** | | ***Lasionycteris noctivagans*** | | ***Corynorhinus rafinesquii*** | | ***Myotis sepentrionalis*** | | **Total** | |
| --- | --- | --- | --- | --- | --- | --- | --- | --- | --- | --- | --- | --- | --- | --- | --- | --- | --- | --- |
| **Season** | S | M | S | M | S | M | S | M | S | M | S | M | S | M | S | M | S | M |
|  | A | W | A | W | A | W | A | W | A | W | A | W | A | W | A | W | A | W |
| **Greensboro** | 3 | 27 | 4 | 90 | 1 | 20 | 1 | 67 | 0 | 1 | 0 | 107 | 0 | 0 | 0 | 0 | 9 | 312 |
|  | 0 | 0 | 0 | 0 | 0 | 0 | 0 | 0 | 0 | 0 | 0 | 0 | 0 | 0 | 0 | 0 | 0 | 0 |
| **Uwharrie National Forest** | 37 | 167 | 481 | 275 | 4 | 0 | 11 | 0 | 598 | 0 | 134 | 0 | 0 | 0 | 0 | 0 | 1265 | 442 |
|  | 4 | 6 | 94 | 5 | 0 | 0 | 0 | 0 | 0 | 0 | 0 | 0 | 0 | 0 | 0 | 0 | 98 | 12 |
| **North River** | 142 | 35 | 1863 | 895 | 276 | 170 | 28 | 4 | 0 | 0 | 1 | 0 | 0 | 1 | 82 | 5 | 2392 | 1110 |
|  | 1 | 0 | 15 | 14 | 7 | 1 | 0 | 0 | 0 | 0 | 0 | 2 | 0 | 0 | 2 | 4 | 25 | 21 |
| **Parker Tract** | 1 | 3 | 9 | 7 | 0 | 2 | 0 | 0 | 0 | 0 | 0 | 0 | 0 | 1 | 11 | 29 | 19 | 44 |
|  | 1 | 0 | 0 | 0 | 0 | 0 | 0 | 0 | 0 | 3 | 0 | 3 | 0 | 0 | 4 | 0 | 5 | 6 |
| **Lenoir 1** | 0 | 0 | 0 | 0 | 0 | 0 | 0 | 0 | 0 | 0 | 0 | 0 | 0 | 0 | 0 | 0 | 0 | 0 |
|  | 0 | 0 | 0 | 0 | 0 | 0 | 3 | 0 | 0 | 0 | 0 | 0 | 0 | 0 | 0 | 0 | 0 | 3 |
| **South River** | 23 | 59 | 623 | 77 | 12 | 3 | 2 | 1 | 0 | 0 | 0 | 0 | 0 | 0 | 0 | 2 | 669 | 142 |
|  | 3 | 33 | 15 | 531 | 0 | 6 | 0 | 54 | 0 | 2 | 0 | 13 | 0 | 0 | 7 | 0 | 25 | 639 |
