# Supplementary material for: Winter Activity of Coastal Plain Populations of Bat Species Affected by White-Nose Syndrome and Wind Energy Facilities
Source: PLoS One. 2016 Nov 16;11(11):e0166512. doi: 10.1371/journal.pone.0166512 (PMC5112809; doi:10.1371/journal.pone.0166512)
Supplement: S2 Table — The numbers in brackets represent the number of female and male bats captures (female, male). In some cases where the bat escaped before gender could be determined, the number of males and females will not add up to the total. (DOCX) [file pone.0166512.s002.docx]

**S2 Table. Capture data from mist-netting in 2012 and 2013 on the Coastal Plain of North Carolina.** The numbers in brackets represent the number of female and male bats captures (female, male). In some cases where the bat escaped before gender could be determined, the number of males and females will not add up to the total.

| **Summer 2012** **(14 May to 5 Aug)** | | | | | | | | | | |
| --- | --- | --- | --- | --- | --- | --- | --- | --- | --- | --- |
| **Site** | *Perimyotis subflavus* | *Lasiurus borealis* | *Nycticeius humeralis* | *Eptesicus fuscus* | *Lasionycteris noctivagans* | *Lasiurus seminolus* | *Corynorhinus rafinesquii* | *Myotis septentrionalis* | *Myotis austroriparius* | Total |
| **North River** | 8 (3,5) | 55 (44,9) | 88 (27,59) | 18 (7,11) | 0 | 0 | 13 (6,5) | 6 (3,3) | 36 (26,10) | 224 |
| **Parker Tract** | 5 (3,2) | 23 (18,5) | 22 (17,5) | 8 (7,1) | 0 | 0 | 0 | 2 (1,1) | 0 | 60 |
| **Lenoir 1** | 3 (1,2) | 27 (22,5) | 0 | 5 (3,2) | 0 | 3 (1,2) | 0 | 0 | 0 | 38 |
| **South River** | 34 (18,15) | 68 (42,25) | 7 (0,6) | 4 (1,3) | 0 | 4 (1,3) | 7 (1,6) | 0 | 6 (4,2) | 130 |
| **Total** | 50 | 173 | 117 | 35 | 0 | 7 | 20 | 8 | 42 | 452 |
|  |  |  |  |  |  |  |  |  |  |  |
| **Spring 2013 (11 Mar and 12 April)** | | | | | | | | | | |
| **Site** | *Perimyotis subflavus* | *Lasiurus borealis* | *Nycticeius humeralis* | *Eptesicus fuscus* | *Lasionycteris noctivagans* | *Lasiurus seminolus* | *Corynorhinus rafinesquii* | *Myotis septentrionalis* | *Myotis austroriparius* | Total |
| **North River** | 2 (0,2) | 2 (0,1) | 0 | 0 | 1 (0,1) | 0 | 2 (1,1) | 1 (0,1) | 2 (0,2) | 10 |
| **Parker Tract** | N/A | N/A | N/A | N/A | N/A | N/A | N/A | N/A | N/A | N/A |
| **Lenoir 1** | N/A | N/A | N/A | N/A | N/A | N/A | N/A | N/A | N/A | N/A |
| **South River** | 0 | 5 (0,5) | 0 | 0 | 0 | 0 | 0 | 0 | 0 | 5 |
| **Total** | 2 | 7 | 0 | 0 | 1 | 0 | 2 | 1 | 2 | 15 |
|  |  |  |  |  |  |  |  |  |  |  |
| **Winter 2013** | | | | | | | | | | |
| **Site** | *Perimyotis subflavus* | *Lasiurus borealis* | *Nycticeius humeralis* | *Eptesicus fuscus* | *Lasionycteris noctivagans* | *Lasiurus seminolus* | *Corynorhinus rafinesquii* | *Myotis septentrionalis* | *Myotis austroriparius* | Total |
| **North River** | 0 | 2 (0,2) | 5 (0,5) | 0 | 0 | 0 | 0 | 0 | 15 (7,7) | 22 |
| **Parker Tract** | N/A | N/A | N/A | N/A | N/A | N/A | N/A | N/A | N/A | N/A |
| **Lenoir 1** | N/A | N/A | N/A | N/A | N/A | N/A | N/A | N/A | N/A | N/A |
| **South River** | N/A | N/A | N/A | N/A | N/A | N/A | N/A | N/A | N/A | N/A |
| **Total** | 0 | 2 | 5 | 0 | 0 | 0 | 0 | 0 | 15 | 22 |
