# Supplementary material for: Winter Activity of Coastal Plain Populations of Bat Species Affected by White-Nose Syndrome and Wind Energy Facilities
Source: PLoS One. 2016 Nov 16;11(11):e0166512. doi: 10.1371/journal.pone.0166512 (PMC5112809; doi:10.1371/journal.pone.0166512)
Supplement: S3 Table — Data were collected in the Coastal Plain and Piedmont regions of North Carolina from 1 September 2012 to 31 August 2014. Best fit model: Activity ~ Temperature * Region * Season − Temperature: Season + (Temperature: Season | Site). (DOCX) [file pone.0166512.s003.docx]

**S3 Table. Coefficient estimates from the best fit model re-leveling the reference groups of region and season.** Data were collected in the Coastal Plain and Piedmont regions of North Carolina from 1 September 2012 to 31 August 2014. Best fit model: Activity ~ Temperature * Region * Season – Temperature: Season + (Temperature: Season | Site).

|  | Estimate | SE | z | P value |
| --- | --- | --- | --- | --- |
| Intercept_Piedmont and Summer_ | 2.89 | 1.03 | 2.79 | 0.01 |
| Temperature | 0.09 | 0.06 | 1.58 | 0.11 |
| Region_Coastal Plain_ | -0.27 | 1.21 | -0.22 | 0.82 |
| Season_Winter_ | -4.15 | 0.88 | -4.71 | 0.00 |
| Temperature : Region_Coastal Plain_ | -0.05 | 0.07 | -0.76 | 0.45 |
| Region_Coastal Plain_ : Season_Winter_ | 0.29 | 1.02 | 0.29 | 0.77 |
| Temperature : Region_Piedmont_ : Season_Winter_ | 0.23 | 0.09 | 2.42 | 0.02 |
| Temperature : Region_Coastal Plain_ : Season_Winter_ | 0.25 | 0.06 | 3.89 | 0.00 |
|  |  |  |  |  |
| Intercept_Piedmont and Winter_ | -1.26 | 0.60 | -2.09 | 0.04 |
| Temperature | 0.32 | 0.08 | 4.11 | 0.00 |
| Region_Coastal Plain_ | 0.02 | 0.75 | 0.03 | 0.98 |
| Season_Summer_ | 4.15 | 0.88 | 4.71 | 0.00 |
| Temperature : Region_Coastal Plain_ | -0.03 | 0.09 | -0.32 | 0.75 |
| Region_Coastal Plain_ : Season_Summer_ | -0.29 | 1.02 | -0.29 | 0.77 |
| Temperature : Region_Piedmont_ : Season_Summer_ | -0.23 | 0.09 | -2.42 | 0.02 |
| Temperature : Region_Coastal Plain_ : Season_Summer_ | -0.25 | 0.06 | -3.89 | 0.00 |
|  |  |  |  |  |
| Intercept_Coastal Plain and Summer_ | 2.61 | 0.63 | 4.14 | 0.00 |
| Temperature | 0.04 | 0.03 | 1.11 | 0.27 |
| Region_Piedmont_ | 0.27 | 1.21 | 0.22 | 0.82 |
| Season_Winter_ | -3.86 | 0.51 | -7.60 | 0.00 |
| Temperature : Region_Piedmont_ | 0.05 | 0.07 | 0.76 | 0.45 |
| Region_Piedmont_ : Season_Winter_ | -0.29 | 1.02 | -0.29 | 0.77 |
| Temperature : Region_Coastal Plain_ : Season_Winter_ | 0.25 | 0.06 | 3.89 | 0.00 |
| Temperature : Region_Piedmont_ : Season_Winter_ | 0.23 | 0.09 | 2.42 | 0.02 |
|  |  |  |  |  |
| Intercept_Coastal Plain and Winter_ | -1.24 | 0.44 | -2.83 | 0.00 |
| Temperature | 0.29 | 0.05 | 5.28 | 0.00 |
| Region_Piedmont_ | -0.02 | 0.75 | -0.03 | 0.98 |
| Season_Summer_ | 3.86 | 0.51 | 7.60 | 0.00 |
| Temperature : Region_Piedmont_ | 0.03 | 0.09 | 0.32 | 0.75 |
| Region_Piedmont_ : Season_Summer_ | 0.29 | 1.02 | 0.29 | 0.77 |
| Temperature : Region_Coastal Plain_ : Season_Summer_ | -0.25 | 0.06 | -3.89 | 0.00 |
| Temperature : Region_Piedmont_ : Season_Summer_ | -0.23 | 0.09 | -2.42 | 0.02 |
