# Supplementary material for: Winter Activity of Coastal Plain Populations of Bat Species Affected by White-Nose Syndrome and Wind Energy Facilities
Source: PLoS One. 2016 Nov 16;11(11):e0166512. doi: 10.1371/journal.pone.0166512 (PMC5112809; doi:10.1371/journal.pone.0166512)
Supplement: S4 Table — (PDF) [file pone.0166512.s004.pdf]

date	season	temp	celsius	calls	site	region
9/1/2012	fall	74.325	23.51388889	37	greensboro	peidmont
9/2/2012	fall	69.99166667	21.10648148	8	greensboro	peidmont
9/3/2012	fall	72.26666667	22.37037037	20	greensboro	peidmont
9/4/2012	fall	72.86666667	22.7037037	9	greensboro	peidmont
9/5/2012	fall	73.675	23.15277778	31	greensboro	peidmont
9/6/2012	fall	69.89166667	21.05092593	30	greensboro	peidmont
9/7/2012	fall	69.76666667	20.98148148	41	greensboro	peidmont
9/8/2012	fall	65.09166667	18.38425926	58	greensboro	peidmont
9/9/2012	fall	59.14166667	15.0787037	60	greensboro	peidmont
9/10/2012	fall	56.81666667	13.78703704	16	greensboro	peidmont
9/11/2012	fall	56.94166667	13.85648148	15	greensboro	peidmont
9/12/2012	fall	55.95	13.30555556	15	greensboro	peidmont
9/13/2012	fall	58.975	14.98611111	12	greensboro	peidmont
9/14/2012	fall	61.78461538	16.54700855	30	greensboro	peidmont
9/15/2012	fall	63.82307692	17.67948718	18	greensboro	peidmont
9/16/2012	fall	60.96153846	16.08974359	6	greensboro	peidmont
9/17/2012	fall	71.25384615	21.80769231	16	greensboro	peidmont
9/18/2012	fall	64.92307692	18.29059829	24	greensboro	peidmont
9/19/2012	fall	55.19230769	12.88461538	15	greensboro	peidmont
9/20/2012	fall	58.44615385	14.69230769	17	greensboro	peidmont
9/21/2012	fall	65.13846154	18.41025641	19	greensboro	peidmont
9/22/2012	fall	64.10769231	17.83760684	20	greensboro	peidmont
9/23/2012	fall	52.53846154	11.41025641	65	greensboro	peidmont
9/24/2012	fall	47.73846154	8.743589744	4	greensboro	peidmont
9/25/2012	fall	58.93076923	14.96153846	10	greensboro	peidmont
9/26/2012	fall	61.16923077	16.20512821	24	greensboro	peidmont
9/27/2012	fall	61.16153846	16.2008547	10	greensboro	peidmont
9/28/2012	fall	64.83846154	18.24358974	9	greensboro	peidmont
9/29/2012	fall	58.43846154	14.68803419	2	greensboro	peidmont
9/30/2012	fall	59.07692308	15.04273504	6	greensboro	peidmont
10/1/2012	fall	62.36923077	16.87179487	22	greensboro	peidmont
10/2/2012	fall	71.53076923	21.96153846	17	greensboro	peidmont

10/3/2012	fall	68.72307692	20.4017094	11	greensboro	peidmont
10/4/2012	fall	59.54615385	15.3034188	23	greensboro	peidmont
10/5/2012	fall	60.07692308	15.5982906	14	greensboro	peidmont
10/6/2012	fall	56.70769231	13.72649573	16	greensboro	peidmont
10/7/2012	fall	49.20769231	9.55982906	2	greensboro	peidmont
10/8/2012	fall	45.23846154	7.354700855	0	greensboro	peidmont
10/9/2012	fall	53.03076923	11.68376068	4	greensboro	peidmont
10/10/2012	fall	51.83846154	11.02136752	11	greensboro	peidmont
10/11/2012	fall	45.85384615	7.696581197	2	greensboro	peidmont
10/12/2012	fall	48.93076923	9.405982906	16	greensboro	peidmont
10/13/2012	fall	46.83076923	8.239316239	13	greensboro	peidmont
10/14/2012	fall	64.3	17.94444444	19	greensboro	peidmont
10/15/2012	fall	51.33076923	10.73931624	23	greensboro	peidmont
10/16/2012	fall	47.15384615	8.418803419	6	greensboro	peidmont
10/17/2012	fall	50.72307692	10.4017094	5	greensboro	peidmont
10/18/2012	fall	61.24285714	16.24603175	12	greensboro	peidmont
10/19/2012	fall	49.17142857	9.53968254	7	greensboro	peidmont
10/20/2012	fall	48.72857143	9.293650794	10	greensboro	peidmont
10/21/2012	fall	44.38571429	6.880952381	3	greensboro	peidmont
10/22/2012	fall	50.58571429	10.32539683	7	greensboro	peidmont
10/23/2012	fall	55.97857143	13.32142857	9	greensboro	peidmont
10/24/2012	fall	54.07142857	12.26190476	12	greensboro	peidmont
10/25/2012	fall	57.03571429	13.90873016	4	greensboro	peidmont
10/26/2012	fall	61.37142857	16.31746032	44	greensboro	peidmont
10/27/2012	fall	59.4	15.22222222	43	greensboro	peidmont
10/28/2012	fall	50.77333333	10.42962963	7	greensboro	peidmont
10/30/2012	fall	41.28666667	5.159259259	0	greensboro	peidmont
10/31/2012	fall	45.55333333	7.52962963	0	greensboro	peidmont
11/1/2012	fall	42.02666667	5.57037037	0	greensboro	peidmont
11/2/2012	fall	45.58	7.544444444	1	greensboro	peidmont
11/3/2012	fall	45.89285714	7.718253968	3	greensboro	peidmont
11/4/2012	fall	43.67142857	6.484126984	2	greensboro	peidmont
11/5/2012	fall	38.18571429	3.436507937	0	greensboro	peidmont

11/6/2012	fall	37.32142857	2.956349206	0	greensboro	peidmont
11/7/2012	fall	37.63571429	3.130952381	1	greensboro	peidmont
11/8/2012	fall	40.32857143	4.626984127	9	greensboro	peidmont
11/9/2012	fall	41.1	5.055555556	3	greensboro	peidmont
11/10/2012	fall	45.27142857	7.373015873	5	greensboro	peidmont
11/11/2012	fall	56.49285714	13.60714286	5	greensboro	peidmont
11/12/2012	fall	56.07857143	13.37698413	5	greensboro	peidmont
11/13/2012	fall	37.72857143	3.182539683	4	greensboro	peidmont
11/14/2012	fall	38.56428571	3.646825397	0	greensboro	peidmont
11/15/2012	fall	39.68571429	4.26984127	0	greensboro	peidmont
11/16/2012	fall	40.07142857	4.484126984	3	greensboro	peidmont
11/17/2012	fall	45.27857143	7.376984127	5	greensboro	peidmont
11/18/2012	fall	49	9.444444444	4	greensboro	peidmont
11/19/2012	fall	44.67857143	7.043650794	1	greensboro	peidmont
11/20/2012	fall	41.48571429	5.26984127	0	greensboro	peidmont
11/21/2012	fall	42.8	6	3	greensboro	peidmont
11/22/2012	fall	36.82857143	2.682539683	0	greensboro	peidmont
11/23/2012	fall	49.33571429	9.630952381	2	greensboro	peidmont
11/24/2012	fall	29.5	-1.388888889	1	greensboro	peidmont
11/25/2012	fall	38.3	3.5	0	greensboro	peidmont
11/26/2012	fall	47.86428571	8.813492063	1	greensboro	peidmont
11/27/2012	fall	40.7	4.833333333	0	greensboro	peidmont
11/28/2012	fall	32.50714286	0.281746032	1	greensboro	peidmont
11/29/2012	fall	37.79285714	3.218253968	1	greensboro	peidmont
11/30/2012	fall	40.66428571	4.813492063	0	greensboro	peidmont
12/1/2012	Winter	44.52142857	6.956349206	0	greensboro	peidmont
12/2/2012	Winter	55.00714286	12.78174603	1	greensboro	peidmont
12/3/2012	Winter	55.42666667	13.01481481	0	greensboro	peidmont
12/4/2012	Winter	60.19333333	15.66296296	2	greensboro	peidmont
12/5/2012	Winter	46.78666667	8.214814815	4	greensboro	peidmont
12/6/2012	Winter	44.93333333	7.185185185	2	greensboro	peidmont
12/7/2012	Winter	47.93333333	8.851851852	0	greensboro	peidmont
12/8/2012	Winter	54.88	12.71111111	2	greensboro	peidmont

12/9/2012	Winter	59.92	15.51111111	1	greensboro	peidmont
12/10/2012	Winter	59.59333333	15.32962963	1	greensboro	peidmont
12/11/2012	Winter	42.58666667	5.881481481	1	greensboro	peidmont
12/12/2012	Winter	41.48	5.266666667	1	greensboro	peidmont
12/13/2012	Winter	33.80666667	1.003703704	0	greensboro	peidmont
12/14/2012	Winter	37.59333333	3.107407407	0	greensboro	peidmont
12/15/2012	Winter	49.25333333	9.585185185	0	greensboro	peidmont
12/16/2012	Winter	53.32	11.84444444	1	greensboro	peidmont
12/17/2012	Winter	55.00666667	12.78148148	0	greensboro	peidmont
12/18/2012	Winter	42.61333333	5.896296296	0	greensboro	peidmont
12/19/2012	Winter	44.3	6.833333333	0	greensboro	peidmont
12/20/2012	Winter	48.50666667	9.17037037	0	greensboro	peidmont
12/21/2012	Winter	36.39333333	2.440740741	0	greensboro	peidmont
12/22/2012	Winter	35.28666667	1.825925926	0	greensboro	peidmont
12/23/2012	Winter	44.72	7.066666667	0	greensboro	peidmont
12/24/2012	Winter	44.68666667	7.048148148	0	greensboro	peidmont
12/25/2012	Winter	45.22	7.344444444	4	greensboro	peidmont
12/26/2012	Winter	37.14666667	2.859259259	0	greensboro	peidmont
12/27/2012	Winter	33.32666667	0.737037037	0	greensboro	peidmont
12/28/2012	Winter	36.56666667	2.537037037	0	greensboro	peidmont
12/29/2012	Winter	37.96875	3.315972222	0	greensboro	peidmont
12/30/2012	Winter	32.05	0.027777778	0	greensboro	peidmont
12/31/2012	Winter	45.7375	7.631944444	0	greensboro	peidmont
1/1/2013	Winter	46.28125	7.934027778	0	greensboro	peidmont
1/2/2013	Winter	39.81875	4.34375	2	greensboro	peidmont
1/3/2013	Winter	32.80625	0.447916667	0	greensboro	peidmont
1/4/2013	Winter	35.275	1.819444444	0	greensboro	peidmont
1/5/2013	Winter	41.16875	5.09375	0	greensboro	peidmont
1/7/2013	Winter	33.90625	1.059027778	0	greensboro	peidmont
1/8/2013	Winter	43.6875	6.493055556	0	greensboro	peidmont
1/9/2013	Winter	53.16875	11.76041667	1	greensboro	peidmont
1/10/2013	Winter	48.025	8.902777778	2	greensboro	peidmont
1/11/2013	Winter	47.28125	8.489583333	3	greensboro	peidmont

1/12/2013	Winter	56.49375	13.60763889	4	greensboro	peidmont
1/13/2013	Winter	63.3125	17.39583333	3	greensboro	peidmont
1/14/2013	Winter	52.61333333	11.45185185	0	greensboro	peidmont
1/15/2013	Winter	40.11333333	4.507407407	0	greensboro	peidmont
1/16/2013	Winter	49.18666667	9.548148148	0	greensboro	peidmont
1/17/2013	Winter	33.07142857	0.595238095	0	greensboro	peidmont
1/18/2013	Winter	29.24285714	-1.531746032	0	greensboro	peidmont
1/19/2013	Winter	40.13571429	4.51984127	0	greensboro	peidmont
1/20/2013	Winter	38.77857143	3.765873016	0	greensboro	peidmont
1/21/2013	Winter	38.8	3.777777778	0	greensboro	peidmont
1/22/2013	Winter	24.35714286	-4.246031746	0	greensboro	peidmont
1/23/2013	Winter	31.92857143	-0.03968254	0	greensboro	peidmont
1/24/2013	Winter	23.23571429	-4.869047619	0	greensboro	peidmont
1/25/2013	Winter	25.3	-3.722222222	0	greensboro	peidmont
1/26/2013	Winter	29.31428571	-1.492063492	0	greensboro	peidmont
1/27/2013	Winter	34.72142857	1.511904762	0	greensboro	peidmont
1/28/2013	Winter	46.70714286	8.170634921	2	greensboro	peidmont
1/29/2013	Winter	63.24285714	17.35714286	4	greensboro	peidmont
1/30/2013	Winter	56.80714286	13.78174603	0	greensboro	peidmont
1/31/2013	Winter	37.50714286	3.05952381	0	greensboro	peidmont
2/1/2013	Winter	24.26428571	-4.297619048	0	greensboro	peidmont
2/2/2013	Winter	37.09285714	2.829365079	0	greensboro	peidmont
2/3/2013	Winter	34.94285714	1.634920635	0	greensboro	peidmont
2/4/2013	Winter	40.56428571	4.757936508	0	greensboro	peidmont
2/5/2013	Winter	41.09285714	5.051587302	2	greensboro	peidmont
2/6/2013	Winter	41.28571429	5.158730159	7	greensboro	peidmont
2/7/2013	Winter	37.12142857	2.845238095	0	greensboro	peidmont
2/8/2013	Winter	39.27142857	4.03968254	0	greensboro	peidmont
2/9/2013	Winter	33.44285714	0.801587302	2	greensboro	peidmont
2/10/2013	Winter	45.37142857	7.428571429	0	greensboro	peidmont
2/11/2013	Winter	51.62857143	10.9047619	1	greensboro	peidmont
2/12/2013	Winter	47.15	8.416666667	2	greensboro	peidmont
2/13/2013	Winter	38.18571429	3.436507937	1	greensboro	peidmont

2/14/2013	Winter	38.10714286	3.392857143	0	greensboro	peidmont
2/15/2013	Winter	43.88571429	6.603174603	1	greensboro	peidmont
2/16/2013	Winter	29.08571429	-1.619047619	0	greensboro	peidmont
2/17/2013	Winter	23.95	-4.472222222	0	greensboro	peidmont
2/18/2013	Winter	39.70714286	4.281746032	2	greensboro	peidmont
2/19/2013	Winter	38.22142857	3.456349206	0	greensboro	peidmont
2/20/2013	Winter	35.72857143	2.071428571	0	greensboro	peidmont
2/21/2013	Winter	39.79285714	4.329365079	3	greensboro	peidmont
2/22/2013	Winter	34.40714286	1.337301587	0	greensboro	peidmont
2/23/2013	Winter	42.36428571	5.757936508	0	greensboro	peidmont
2/24/2013	Winter	42.82857143	6.015873016	5	greensboro	peidmont
2/25/2013	Winter	34.57857143	1.432539683	1	greensboro	peidmont
2/26/2013	Winter	39.85	4.361111111	0	greensboro	peidmont
2/27/2013	Winter	43.7	6.5	1	greensboro	peidmont
2/28/2013	Winter	39.28571429	4.047619048	0	greensboro	peidmont
3/1/2013	Spring	35.92857143	2.182539683	0	greensboro	peidmont
3/2/2013	Spring	32.91428571	0.507936508	0	greensboro	peidmont
3/3/2013	Spring	29.81428571	-1.214285714	0	greensboro	peidmont
3/4/2013	Spring	40.37857143	4.654761905	1	greensboro	peidmont
3/5/2013	Spring	40.25714286	4.587301587	1	greensboro	peidmont
3/6/2013	Spring	38.29285714	3.496031746	0	greensboro	peidmont
3/7/2013	Spring	36.65714286	2.587301587	0	greensboro	peidmont
3/8/2013	Spring	41.04285714	5.023809524	3	greensboro	peidmont
3/9/2013	Spring	41.80714286	5.448412698	6	greensboro	peidmont
3/10/2013	Spring	48.32857143	9.071428571	33	greensboro	peidmont
3/11/2013	Spring	58.47142857	14.70634921	25	greensboro	peidmont
3/12/2013	Spring	42.44285714	5.801587302	6	greensboro	peidmont
3/13/2013	Spring	34.26428571	1.257936508	1	greensboro	peidmont
3/14/2013	Spring	37.74285714	3.19047619	2	greensboro	peidmont
3/15/2013	Spring	51.03846154	10.57692308	5	greensboro	peidmont
3/18/2013	Spring	38.06923077	3.371794872	0	greensboro	peidmont
3/19/2013	Spring	45.78333333	7.657407407	5	greensboro	peidmont
3/20/2013	Spring	42.175	5.652777778	3	greensboro	peidmont

3/21/2013	Spring	27.5	-2.5	0	greensboro	peidmont
3/22/2013	Spring	39.10833333	3.949074074	7	greensboro	peidmont
3/23/2013	Spring	41.13333333	5.074074074	6	greensboro	peidmont
3/24/2013	Spring	34.35	1.305555556	1	greensboro	peidmont
3/25/2013	Spring	36.25	2.361111111	0	greensboro	peidmont
3/26/2013	Spring	36.625	2.569444444	0	greensboro	peidmont
3/27/2013	Spring	36.44166667	2.467592593	1	greensboro	peidmont
3/28/2013	Spring	35.5	1.944444444	4	greensboro	peidmont
3/29/2013	Spring	45.60833333	7.560185185	4	greensboro	peidmont
3/30/2013	Spring	51.18333333	10.65740741	7	greensboro	peidmont
3/31/2013	Spring	53.4	11.88888889	3	greensboro	peidmont
4/1/2013	Spring	45.45	7.472222222	8	greensboro	peidmont
4/2/2013	Spring	43.69166667	6.49537037	2	greensboro	peidmont
4/3/2013	Spring	44.1	6.722222222	4	greensboro	peidmont
4/4/2013	Spring	36.925	2.736111111	0	greensboro	peidmont
4/5/2013	Spring	47.375	8.541666667	5	greensboro	peidmont
4/6/2013	Spring	48.06666667	8.925925926	20	greensboro	peidmont
4/7/2013	Spring	56.83333333	13.7962963	34	greensboro	peidmont
4/8/2013	Spring	61.85	16.58333333	32	greensboro	peidmont
4/9/2013	Spring	67.16666667	19.53703704	28	greensboro	peidmont
4/10/2013	Spring	66.84166667	19.35648148	52	greensboro	peidmont
4/11/2013	Spring	69.025	20.56944444	35	greensboro	peidmont
4/12/2013	Spring	56.58333333	13.65740741	49	greensboro	peidmont
4/13/2013	Spring	52.99166667	11.66203704	28	greensboro	peidmont
4/14/2013	Spring	59.66666667	15.37037037	32	greensboro	peidmont
4/15/2013	Spring	60.18333333	15.65740741	16	greensboro	peidmont
4/16/2013	Spring	61.625	16.45833333	39	greensboro	peidmont
4/17/2013	Spring	65.76666667	18.75925926	34	greensboro	peidmont
4/18/2013	Spring	66.76666667	19.31481481	46	greensboro	peidmont
4/19/2013	Spring	54.475	12.48611111	4	greensboro	peidmont
4/20/2013	Spring	44.675	7.041666667	14	greensboro	peidmont
4/21/2013	Spring	42.28333333	5.712962963	0	greensboro	peidmont
4/22/2013	Spring	49.875	9.930555556	0	greensboro	peidmont

4/23/2013	Spring	52.98333333	11.65740741	22	greensboro	peidmont
4/24/2013	Spring	59.80833333	15.44907407	21	greensboro	peidmont
4/25/2013	Spring	49.31666667	9.62037037	6	greensboro	peidmont
4/26/2013	Spring	54.74166667	12.63425926	12	greensboro	peidmont
4/27/2013	Spring	56.21666667	13.4537037	18	greensboro	peidmont
4/28/2013	Spring	55.34545455	12.96969697	0	greensboro	peidmont
4/29/2013	Spring	57.34545455	14.08080808	11	greensboro	peidmont
4/30/2013	Spring	56.40909091	13.56060606	10	greensboro	peidmont
5/1/2013	Spring	55.97272727	13.31818182	4	greensboro	peidmont
5/2/2013	Spring	52.26363636	11.25757576	7	greensboro	peidmont
5/3/2013	Spring	51.20909091	10.67171717	3	greensboro	peidmont
5/4/2013	Spring	52.40909091	11.33838384	1	greensboro	peidmont
5/5/2013	Spring	54.86363636	12.7020202	0	greensboro	peidmont
5/6/2013	Spring	57.22727273	14.01515152	3	greensboro	peidmont
5/7/2013	Spring	53.80909091	12.11616162	6	greensboro	peidmont
5/8/2013	Spring	54.84545455	12.69191919	5	greensboro	peidmont
5/9/2013	Spring	60.90909091	16.06060606	43	greensboro	peidmont
5/10/2013	Spring	69.38181818	20.76767677	48	greensboro	peidmont
5/11/2013	Spring	63.87272727	17.70707071	18	greensboro	peidmont
5/12/2013	Spring	51.48181818	10.82323232	7	greensboro	peidmont
5/13/2013	Spring	45.53636364	7.52020202	7	greensboro	peidmont
5/14/2013	Spring	57.93636364	14.40909091	62	greensboro	peidmont
5/15/2013	Spring	69.64545455	20.91414141	58	greensboro	peidmont
5/16/2013	Spring	67.92727273	19.95959596	56	greensboro	peidmont
5/17/2013	Spring	66.76363636	19.31313131	32	greensboro	peidmont
5/18/2013	Spring	64.3	17.94444444	10	greensboro	peidmont
5/19/2013	Spring	67.24545455	19.58080808	18	greensboro	peidmont
5/20/2013	Spring	67.76363636	19.86868687	23	greensboro	peidmont
5/21/2013	Spring	70.4	21.33333333	26	greensboro	peidmont
5/22/2013	Spring	67.13636364	19.52020202	15	greensboro	peidmont
5/23/2013	Spring	64.1	17.83333333	10	greensboro	peidmont
5/24/2013	Spring	49.95454545	9.974747475	0	greensboro	peidmont
5/25/2013	Spring	55.71818182	13.17676768	4	greensboro	peidmont

5/26/2013	Spring	55.52727273	13.07070707	15	greensboro	peidmont
5/27/2013	Spring	64.51818182	18.06565657	53	greensboro	peidmont
5/29/2013	Spring	67.18181818	19.54545455	14	greensboro	peidmont
5/30/2013	Spring	67.24545455	19.58080808	17	greensboro	peidmont
5/31/2013	Spring	71.12	21.73333333	26	greensboro	peidmont
6/1/2013	Summer	71.51	21.95	12	greensboro	peidmont
6/2/2013	Summer	69.08	20.6	19	greensboro	peidmont
6/3/2013	Summer	66.01	18.89444444	12	greensboro	peidmont
6/4/2013	Summer	63.14	17.3	4	greensboro	peidmont
6/5/2013	Summer	66.26	19.03333333	10	greensboro	peidmont
6/6/2013	Summer	68.36	20.2	4	greensboro	peidmont
6/7/2013	Summer	68.08	20.04444444	13	greensboro	peidmont
6/8/2013	Summer	68.07	20.03888889	38	greensboro	peidmont
6/9/2013	Summer	71.57	21.98333333	18	greensboro	peidmont
6/10/2013	Summer	69.7	20.94444444	8	greensboro	peidmont
6/11/2013	Summer	67.92	19.95555556	27	greensboro	peidmont
6/12/2013	Summer	77.37	25.20555556	19	greensboro	peidmont
6/13/2013	Summer	66.04	18.91111111	6	greensboro	peidmont
6/14/2013	Summer	61.23	16.23888889	5	greensboro	peidmont
6/15/2013	Summer	68.56	20.31111111	14	greensboro	peidmont
6/16/2013	Summer	72.61	22.56111111	14	greensboro	peidmont
6/17/2013	Summer	71.18	21.76666667	8	greensboro	peidmont
6/18/2013	Summer	67.25	19.58333333	15	greensboro	peidmont
6/19/2013	Summer	66.04	18.91111111	14	greensboro	peidmont
6/20/2013	Summer	64.9	18.27777778	6	greensboro	peidmont
6/21/2013	Summer	65.61	18.67222222	7	greensboro	peidmont
6/22/2013	Summer	67.95	19.97222222	12	greensboro	peidmont
6/24/2013	Summer	71.56	21.97777778	12	greensboro	peidmont
6/25/2013	Summer	71.55	21.97222222	10	greensboro	peidmont
6/27/2013	Summer	73.07	22.81666667	22	greensboro	peidmont
6/28/2013	Summer	70.16	21.2	2	greensboro	peidmont
6/29/2013	Summer	74.56	23.64444444	7	greensboro	peidmont
6/30/2013	Summer	69.16	20.64444444	1	greensboro	peidmont

7/1/2013	Summer	72.44	22.46666667	5	greensboro	peidmont
7/2/2013	Summer	72.18	22.32222222	1	greensboro	peidmont
7/3/2013	Summer	73.48	23.04444444	21	greensboro	peidmont
7/4/2013	Summer	71.79	22.10555556	33	greensboro	peidmont
7/5/2013	Summer	71.35	21.86111111	17	greensboro	peidmont
7/6/2013	Summer	73.24	22.91111111	22	greensboro	peidmont
7/7/2013	Summer	71.86	22.14444444	2	greensboro	peidmont
7/8/2013	Summer	73.32	22.95555556	38	greensboro	peidmont
7/9/2013	Summer	72.67	22.59444444	9	greensboro	peidmont
7/10/2013	Summer	72.35	22.41666667	3	greensboro	peidmont
7/11/2013	Summer	71.9	22.16666667	33	greensboro	peidmont
7/12/2013	Summer	68.55	20.30555556	19	greensboro	peidmont
7/13/2013	Summer	73.08	22.82222222	13	greensboro	peidmont
7/14/2013	Summer	71.33	21.85	33	greensboro	peidmont
7/15/2013	Summer	71.31	21.83888889	42	greensboro	peidmont
7/16/2013	Summer	77.39	25.21666667	29	greensboro	peidmont
7/17/2013	Summer	75.99	24.43888889	42	greensboro	peidmont
7/18/2013	Summer	74.98	23.87777778	29	greensboro	peidmont
7/19/2013	Summer	75.83	24.35	23	greensboro	peidmont
7/20/2013	Summer	73.7	23.16666667	28	greensboro	peidmont
7/21/2013	Summer	71.41	21.89444444	15	greensboro	peidmont
7/22/2013	Summer	74.25	23.47222222	24	greensboro	peidmont
7/23/2013	Summer	72.37	22.42777778	37	greensboro	peidmont
7/24/2013	Summer	70.96	21.64444444	46	greensboro	peidmont
7/25/2013	Summer	66.28	19.04444444	32	greensboro	peidmont
7/26/2013	Summer	68.88	20.48888889	44	greensboro	peidmont
7/27/2013	Summer	68.67272727	20.37373737	12	greensboro	peidmont
7/28/2013	Summer	69.98181818	21.1010101	73	greensboro	peidmont
7/29/2013	Summer	66.8	19.33333333	63	greensboro	peidmont
7/30/2013	Summer	71.33636364	21.85353535	50	greensboro	peidmont
7/31/2013	Summer	70.77272727	21.54040404	8	greensboro	peidmont
8/1/2013	Summer	72.61818182	22.56565657	87	greensboro	peidmont
8/2/2013	Summer	71.52727273	21.95959596	58	greensboro	peidmont

8/3/2013	Summer	73.02727273	22.79292929	88	greensboro	peidmont
8/4/2013	Summer	68.36666667	20.2037037	62	greensboro	peidmont
8/6/2013	Summer	69.24166667	20.68981481	34	greensboro	peidmont
8/7/2013	Summer	72.08333333	22.26851852	81	greensboro	peidmont
8/8/2013	Summer	74.4	23.55555556	60	greensboro	peidmont
8/9/2013	Summer	74.625	23.68055556	70	greensboro	peidmont
8/10/2013	Summer	71.90833333	22.1712963	73	greensboro	peidmont
8/11/2013	Summer	73.50833333	23.06018519	170	greensboro	peidmont
8/12/2013	Summer	72.78333333	22.65740741	74	greensboro	peidmont
8/13/2013	Summer	71.91666667	22.17592593	119	greensboro	peidmont
8/14/2013	Summer	61.875	16.59722222	49	greensboro	peidmont
8/15/2013	Summer	58.975	14.98611111	26	greensboro	peidmont
8/16/2013	Summer	61.64166667	16.46759259	59	greensboro	peidmont
8/17/2013	Summer	65.575	18.65277778	2	greensboro	peidmont
8/18/2013	Summer	65.90833333	18.83796296	10	greensboro	peidmont
8/19/2013	Summer	66.025	18.90277778	2	greensboro	peidmont
8/20/2013	Summer	70.625	21.45833333	40	greensboro	peidmont
8/21/2013	Summer	72.325	22.40277778	16	greensboro	peidmont
8/22/2013	Summer	70.48333333	21.37962963	62	greensboro	peidmont
8/23/2013	Summer	65.40833333	18.56018519	108	greensboro	peidmont
8/24/2013	Summer	62.99166667	17.21759259	46	greensboro	peidmont
8/25/2013	Summer	58.65833333	14.81018519	31	greensboro	peidmont
8/26/2013	Summer	66.53333333	19.18518519	50	greensboro	peidmont
9/27/2013	Fall	55.77692308	13.20940171	18	greensboro	peidmont
9/28/2013	Fall	56.59230769	13.66239316	30	greensboro	peidmont
9/29/2013	Fall	51.93076923	11.07264957	7	greensboro	peidmont
9/30/2013	Fall	55.82307692	13.23504274	13	greensboro	peidmont
10/1/2013	Fall	60.23846154	15.68803419	109	greensboro	peidmont
10/2/2013	Fall	61.71538462	16.50854701	265	greensboro	peidmont
10/3/2013	Fall	65.52307692	18.62393162	20	greensboro	peidmont
10/4/2013	Fall	65.02307692	18.34615385	14	greensboro	peidmont
10/5/2013	Fall	66.52307692	19.17948718	19	greensboro	peidmont
10/6/2013	Fall	70.69230769	21.4957265	11	greensboro	peidmont

10/7/2013	Fall	60.11538462	15.61965812	143	greensboro	peidmont
10/8/2013	Fall	54.76153846	12.64529915	16	greensboro	peidmont
10/10/2013	Fall	61.33076923	16.29487179	28	greensboro	peidmont
10/11/2013	Fall	61.83076923	16.57264957	22	greensboro	peidmont
10/12/2013	Fall	61.83076923	16.57264957	4	greensboro	peidmont
10/13/2013	Fall	59.94615385	15.52564103	4	greensboro	peidmont
10/14/2013	Fall	57.04615385	13.91452991	10	greensboro	peidmont
10/15/2013	Fall	61.27692308	16.26495726	6	greensboro	peidmont
10/16/2013	Fall	62.43846154	16.91025641	6	greensboro	peidmont
10/17/2013	Fall	61.10769231	16.17094017	5	greensboro	peidmont
10/18/2013	Fall	54.54285714	12.52380952	7	greensboro	peidmont
10/19/2013	Fall	52.07857143	11.1547619	5	greensboro	peidmont
10/20/2013	Fall	41.76428571	5.424603175	0	greensboro	peidmont
10/21/2013	Fall	54.15	12.30555556	6	greensboro	peidmont
10/22/2013	Fall	57.19285714	13.99603175	6	greensboro	peidmont
10/23/2013	Fall	41.22142857	5.123015873	4	greensboro	peidmont
10/24/2013	Fall	43.17857143	6.21031746	0	greensboro	peidmont
10/25/2013	Fall	34.35714286	1.30952381	1	greensboro	peidmont
10/26/2013	Fall	49.22857143	9.571428571	1	greensboro	peidmont
10/27/2013	Fall	47.29285714	8.496031746	3	greensboro	peidmont
10/28/2013	Fall	54.46666667	12.48148148	5	greensboro	peidmont
10/29/2013	Fall	54.59333333	12.55185185	4	greensboro	peidmont
10/30/2013	Fall	61.44666667	16.35925926	28	greensboro	peidmont
10/31/2013	Fall	67.9	19.94444444	9	greensboro	peidmont
11/1/2013	Fall	57.81333333	14.34074074	9	greensboro	peidmont
11/2/2013	Fall	49.76428571	9.869047619	9	greensboro	peidmont
11/3/2013	Fall	44.46	6.922222222	3	greensboro	peidmont
11/4/2013	Fall	40.55333333	4.751851852	0	greensboro	peidmont
11/5/2013	Fall	50.02	10.01111111	0	greensboro	peidmont
11/6/2013	Fall	57.12	13.95555556	5	greensboro	peidmont
11/8/2013	Fall	36.19333333	2.32962963	0	greensboro	peidmont
11/9/2013	Fall	43.86666667	6.592592593	0	greensboro	peidmont
11/10/2013	Fall	49.34666667	9.637037037	9	greensboro	peidmont

11/11/2013	Fall	45.04	7.244444444	0	greensboro	peidmont
11/12/2013	Fall	31.96666667	-0.018518519	1	greensboro	peidmont
11/13/2013	Fall	30.05333333	-1.081481481	0	greensboro	peidmont
11/14/2013	Fall	39.18	3.988888889	0	greensboro	peidmont
11/15/2013	Fall	51.09333333	10.60740741	2	greensboro	peidmont
11/16/2013	Fall	54.76	12.64444444	0	greensboro	peidmont
11/17/2013	Fall	63.16	17.31111111	5	greensboro	peidmont
11/19/2013	Fall	41.92666667	5.514814815	2	greensboro	peidmont
11/20/2013	Fall	41.7	5.388888889	1	greensboro	peidmont
11/21/2013	Fall	50.08	10.04444444	0	greensboro	peidmont
11/22/2013	Fall	60.61333333	15.8962963	2	greensboro	peidmont
11/23/2013	Fall	40.69333333	4.82962963	0	greensboro	peidmont
11/24/2013	Fall	26.21333333	-3.214814815	0	greensboro	peidmont
11/25/2013	Fall	36.36	2.422222222	0	greensboro	peidmont
11/26/2013	Fall	43.62666667	6.459259259	0	greensboro	peidmont
11/27/2013	Fall	28.57333333	-1.903703704	1	greensboro	peidmont
11/28/2013	Fall	29.58	-1.344444444	0	greensboro	peidmont
11/29/2013	Fall	33.77333333	0.985185185	0	greensboro	peidmont
11/30/2013	Fall	31.08666667	-0.507407407	0	greensboro	peidmont
12/1/2013	Winter	38.45333333	3.585185185	1	greensboro	peidmont
12/2/2013	Winter	44.37333333	6.874074074	3	greensboro	peidmont
12/3/2013	Winter	50.16	10.08888889	2	greensboro	peidmont
12/4/2013	Winter	57.92666667	14.4037037	2	greensboro	peidmont
12/5/2013	Winter	64.84666667	18.24814815	1	greensboro	peidmont
12/7/2013	Winter	41.54	5.3	2	greensboro	peidmont
12/8/2013	Winter	32.51333333	0.285185185	0	greensboro	peidmont
12/9/2013	Winter	43.10666667	6.17037037	0	greensboro	peidmont
12/10/2013	Winter	33.21333333	0.674074074	0	greensboro	peidmont
12/11/2013	Winter	37.66666667	3.148148148	0	greensboro	peidmont
12/12/2013	Winter	31.34	-0.366666667	0	greensboro	peidmont
12/13/2013	Winter	39.59333333	4.218518519	0	greensboro	peidmont
12/14/2013	Winter	37.08	2.822222222	0	greensboro	peidmont
12/15/2013	Winter	35.50666667	1.948148148	1	greensboro	peidmont

12/16/2013	Winter	43.31333333	6.285185185	0	greensboro	peidmont
12/17/2013	Winter	45.82	7.677777778	1	greensboro	peidmont
12/18/2013	Winter	35.6	2	0	greensboro	peidmont
12/19/2013	Winter	48.72666667	9.292592593	0	greensboro	peidmont
12/20/2013	Winter	55.12	12.84444444	1	greensboro	peidmont
12/22/2013	Winter	62.78666667	17.1037037	6	greensboro	peidmont
12/23/2013	Winter	43.82666667	6.57037037	1	greensboro	peidmont
12/24/2013	Winter	29.17333333	-1.57037037	0	greensboro	peidmont
12/25/2013	Winter	27.07333333	-2.737037037	0	greensboro	peidmont
12/26/2013	Winter	36.38666667	2.437037037	0	greensboro	peidmont
12/27/2013	Winter	33.44666667	0.803703704	0	greensboro	peidmont
12/28/2013	Winter	45.07333333	7.262962963	2	greensboro	peidmont
12/29/2013	Winter	44.91875	7.177083333	0	greensboro	peidmont
12/30/2013	Winter	40.6375	4.798611111	0	greensboro	peidmont
12/31/2013	Winter	36.9375	2.743055556	0	greensboro	peidmont
1/1/2014	Winter	38.41875	3.565972222	0	greensboro	peidmont
1/2/2014	Winter	34.1875	1.215277778	0	greensboro	peidmont
1/3/2014	Winter	21.3625	-5.909722222	0	greensboro	peidmont
1/4/2014	Winter	33.16875	0.649305556	0	greensboro	peidmont
1/5/2014	Winter	41.6125	5.340277778	0	greensboro	peidmont
1/13/2014	Winter	48.05625	8.920138889	0	greensboro	peidmont
1/15/2014	Winter	37.58	3.1	0	greensboro	peidmont
1/16/2014	Winter	34.31333333	1.285185185	0	greensboro	peidmont
1/17/2014	Winter	38.77857143	3.765873016	0	greensboro	peidmont
1/18/2014	Winter	31.35714286	-0.357142857	0	greensboro	peidmont
1/19/2014	Winter	37.82857143	3.238095238	0	greensboro	peidmont
1/20/2014	Winter	40.35	4.638888889	0	greensboro	peidmont
1/21/2014	Winter	26.33571429	-3.146825397	0	greensboro	peidmont
1/22/2014	Winter	21.35714286	-5.912698413	0	greensboro	peidmont
1/23/2014	Winter	18.88571429	-7.285714286	0	greensboro	peidmont
1/24/2014	Winter	19.47857143	-6.956349206	0	greensboro	peidmont
1/25/2014	Winter	31.6	-0.222222222	0	greensboro	peidmont
1/26/2014	Winter	44.19285714	6.773809524	0	greensboro	peidmont

1/27/2014	Winter	32.5	0.277777778	0	greensboro	peidmont
1/28/2014	Winter	17.56428571	-8.01984127	0	greensboro	peidmont
1/29/2014	Winter	13.69285714	-10.17063492	0	greensboro	peidmont
1/30/2014	Winter	21.45	-5.861111111	0	greensboro	peidmont
1/31/2014	Winter	33.27857143	0.71031746	0	greensboro	peidmont
2/1/2014	Winter	46.88571429	8.26984127	0	greensboro	peidmont
2/2/2014	Winter	54.14285714	12.3015873	3	greensboro	peidmont
2/3/2014	Winter	38.7	3.722222222	1	greensboro	peidmont
2/4/2014	Winter	36.9	2.722222222	0	greensboro	peidmont
2/5/2014	Winter	37.77142857	3.206349206	0	greensboro	peidmont
2/6/2014	Winter	35.85	2.138888889	0	greensboro	peidmont
2/7/2014	Winter	36.88571429	2.714285714	0	greensboro	peidmont
2/8/2014	Winter	38.07142857	3.373015873	0	greensboro	peidmont
2/9/2014	Winter	42.55714286	5.865079365	1	greensboro	peidmont
2/10/2014	Winter	32.22142857	0.123015873	0	greensboro	peidmont
2/11/2014	Winter	25.67142857	-3.515873016	0	greensboro	peidmont
2/12/2014	Winter	24.8	-4	0	greensboro	peidmont
2/13/2014	Winter	33.99285714	1.107142857	0	greensboro	peidmont
2/14/2014	Winter	41.27857143	5.154761905	0	greensboro	peidmont
2/15/2014	Winter	30.75	-0.694444444	0	greensboro	peidmont
2/16/2014	Winter	37.05714286	2.80952381	0	greensboro	peidmont
2/17/2014	Winter	37.17857143	2.876984127	0	greensboro	peidmont
2/18/2014	Winter	48.03571429	8.908730159	3	greensboro	peidmont
2/19/2014	Winter	54.52857143	12.51587302	1	greensboro	peidmont
2/20/2014	Winter	61.12857143	16.18253968	0	greensboro	peidmont
2/21/2014	Winter	42.05714286	5.587301587	0	greensboro	peidmont
2/22/2014	Winter	45.61428571	7.563492063	5	greensboro	peidmont
2/23/2014	Winter	50.07857143	10.04365079	0	greensboro	peidmont
2/24/2014	Winter	40.72857143	4.849206349	0	greensboro	peidmont
2/25/2014	Winter	39.21428571	4.007936508	0	greensboro	peidmont
2/26/2014	Winter	30.14285714	-1.031746032	0	greensboro	peidmont
2/27/2014	Winter	33.92857143	1.071428571	0	greensboro	peidmont
2/28/2014	Winter	30.97857143	-0.567460317	0	greensboro	peidmont

3/1/2014	Spring	39.45	4.138888889	0	greensboro	peidmont
3/2/2014	Spring	59.44285714	15.24603175	7	greensboro	peidmont
3/3/2014	Spring	19.64285714	-6.865079365	0	greensboro	peidmont
3/4/2014	Spring	29.30714286	-1.496031746	0	greensboro	peidmont
3/5/2014	Spring	34.3	1.277777778	0	greensboro	peidmont
3/6/2014	Spring	30.75	-0.694444444	1	greensboro	peidmont
3/10/2014	Spring	51.94285714	11.07936508	4	greensboro	peidmont
3/11/2014	Spring	60.77857143	15.98809524	5	greensboro	peidmont
3/12/2014	Spring	37.31428571	2.952380952	0	greensboro	peidmont
3/13/2014	Spring	31.16428571	-0.464285714	0	greensboro	peidmont
3/14/2014	Spring	49.38461538	9.658119658	0	greensboro	peidmont
3/15/2014	Spring	50.24615385	10.13675214	2	greensboro	peidmont
3/16/2014	Spring	33.90769231	1.05982906	0	greensboro	peidmont
3/17/2014	Spring	28.34615385	-2.02991453	0	greensboro	peidmont
3/18/2014	Spring	35.09230769	1.717948718	0	greensboro	peidmont
3/19/2014	Spring	41.84615385	5.47008547	1	greensboro	peidmont
3/20/2014	Spring	42.35	5.75	2	greensboro	peidmont
3/21/2014	Spring	51.06666667	10.59259259	4	greensboro	peidmont
3/22/2014	Spring	52.25833333	11.25462963	4	greensboro	peidmont
3/23/2014	Spring	35.9	2.166666667	0	greensboro	peidmont
3/24/2014	Spring	38.29166667	3.49537037	0	greensboro	peidmont
3/25/2014	Spring	30.71666667	-0.712962963	0	greensboro	peidmont
3/26/2014	Spring	31.125	-0.486111111	0	greensboro	peidmont
3/27/2014	Spring	47.85833333	8.810185185	6	greensboro	peidmont
3/28/2014	Spring	55.85833333	13.25462963	2	greensboro	peidmont
3/29/2014	Spring	47.39166667	8.550925926	1	greensboro	peidmont
3/30/2014	Spring	41.5	5.277777778	0	greensboro	peidmont
3/31/2014	Spring	50.08333333	10.0462963	6	greensboro	peidmont
4/1/2014	Spring	58.34166667	14.63425926	9	greensboro	peidmont
4/3/2014	Spring	61.975	16.65277778	13	greensboro	peidmont
4/6/2014	Spring	49.73333333	9.851851852	0	greensboro	peidmont
4/7/2014	Spring	58.86666667	14.92592593	0	greensboro	peidmont
4/8/2014	Spring	53.41666667	11.89814815	4	greensboro	peidmont

4/9/2014	Spring	47.74166667	8.74537037	5	greensboro	peidmont
4/10/2014	Spring	57.975	14.43055556	12	greensboro	peidmont
4/13/2014	Spring	65.10833333	18.39351852	39	greensboro	peidmont
4/14/2014	Spring	65.44166667	18.5787037	31	greensboro	peidmont
4/15/2014	Spring	34.93333333	1.62962963	0	greensboro	peidmont
4/16/2014	Spring	38.33333333	3.518518519	0	greensboro	peidmont
4/17/2014	Spring	44.34166667	6.856481481	3	greensboro	peidmont
4/18/2014	Spring	48.075	8.930555556	1	greensboro	peidmont
4/19/2014	Spring	52.675	11.48611111	10	greensboro	peidmont
4/20/2014	Spring	48.51666667	9.175925926	7	greensboro	peidmont
4/21/2014	Spring	58.80833333	14.89351852	18	greensboro	peidmont
4/22/2014	Spring	58.43333333	14.68518519	21	greensboro	peidmont
4/24/2014	Spring	55.7	13.16666667	11	greensboro	peidmont
4/25/2014	Spring	56.93333333	13.85185185	0	greensboro	peidmont
4/26/2014	Spring	60.19166667	15.66203704	20	greensboro	peidmont
4/27/2014	Spring	64.60833333	18.11574074	16	greensboro	peidmont
4/28/2014	Spring	52.45	11.36111111	2	greensboro	peidmont
4/29/2014	Spring	53.4	11.88888889	1	greensboro	peidmont
4/30/2014	Spring	68.16666667	20.09259259	9	greensboro	peidmont
5/1/2014	Spring	56.50833333	13.61574074	18	greensboro	peidmont
5/2/2014	Spring	55.74166667	13.18981481	7	greensboro	peidmont
5/3/2014	Spring	59.25	15.13888889	12	greensboro	peidmont
5/4/2014	Spring	64.55454545	18.08585859	19	greensboro	peidmont
5/7/2014	Spring	63.57272727	17.54040404	24	greensboro	peidmont
5/8/2014	Spring	68.25454545	20.14141414	21	greensboro	peidmont
5/9/2014	Spring	65.24545455	18.46969697	13	greensboro	peidmont
5/10/2014	Spring	61.57272727	16.42929293	15	greensboro	peidmont
5/11/2014	Spring	66.09090909	18.93939394	9	greensboro	peidmont
5/12/2014	Spring	69.62727273	20.9040404	20	greensboro	peidmont
5/13/2014	Spring	72.93636364	22.74242424	28	greensboro	peidmont
5/16/2014	Spring	50.25454545	10.14141414	3	greensboro	peidmont
5/17/2014	Spring	57.09090909	13.93939394	6	greensboro	peidmont
5/18/2014	Spring	53.05454545	11.6969697	2	greensboro	peidmont

5/19/2014	Spring	55.39090909	12.99494949	1	greensboro	peidmont
5/20/2014	Spring	65.70909091	18.72727273	32	greensboro	peidmont
5/21/2014	Spring	71.56363636	21.97979798	16	greensboro	peidmont
5/22/2014	Spring	71.2	21.77777778	16	greensboro	peidmont
5/23/2014	Spring	64.41	18.00555556	10	greensboro	peidmont
5/24/2014	Spring	62.42	16.9	7	greensboro	peidmont
5/25/2014	Spring	64.68	18.15555556	18	greensboro	peidmont
5/26/2014	Spring	68.68	20.37777778	9	greensboro	peidmont
5/27/2014	Spring	67.76	19.86666667	21	greensboro	peidmont
5/28/2014	Spring	71.78	22.1	17	greensboro	peidmont
5/29/2014	Spring	67.45	19.69444444	3	greensboro	peidmont
5/31/2014	Spring	65.13	18.40555556	16	greensboro	peidmont
6/1/2014	Summer	56.59	13.66111111	3	greensboro	peidmont
6/2/2014	Summer	61.41	16.33888889	4	greensboro	peidmont
6/3/2014	Summer	69.45	20.80555556	24	greensboro	peidmont
6/4/2014	Summer	75.96	24.42222222	17	greensboro	peidmont
6/5/2014	Summer	67.54	19.74444444	0	greensboro	peidmont
6/6/2014	Summer	64.93	18.29444444	20	greensboro	peidmont
6/7/2014	Summer	68.9	20.5	25	greensboro	peidmont
6/8/2014	Summer	68.57	20.31666667	9	greensboro	peidmont
6/9/2014	Summer	67.64	19.8	13	greensboro	peidmont
6/10/2014	Summer	70.39	21.32777778	13	greensboro	peidmont
6/11/2014	Summer	68.31	20.17222222	16	greensboro	peidmont
6/12/2014	Summer	68.7	20.38888889	14	greensboro	peidmont
6/13/2014	Summer	70.44	21.35555556	14	greensboro	peidmont
6/14/2014	Summer	61.22	16.23333333	4	greensboro	peidmont
6/15/2014	Summer	69.75	20.97222222	5	greensboro	peidmont
6/16/2014	Summer	71.2	21.77777778	15	greensboro	peidmont
6/17/2014	Summer	75.24	24.02222222	18	greensboro	peidmont
6/18/2014	Summer	75.17	23.98333333	12	greensboro	peidmont
6/19/2014	Summer	70.84	21.57777778	11	greensboro	peidmont
6/20/2014	Summer	71.15	21.75	11	greensboro	peidmont
6/21/2014	Summer	71.1	21.72222222	16	greensboro	peidmont

6/22/2014	Summer	69.48	20.82222222	5	greensboro	peidmont
6/23/2014	Summer	69.85	21.02777778	18	greensboro	peidmont
6/24/2014	Summer	72.53	22.51666667	11	greensboro	peidmont
6/25/2014	Summer	72	22.22222222	15	greensboro	peidmont
6/26/2014	Summer	71.86	22.14444444	11	greensboro	peidmont
6/27/2014	Summer	70.23	21.23888889	5	greensboro	peidmont
6/28/2014	Summer	68.72	20.4	8	greensboro	peidmont
6/29/2014	Summer	67.03	19.46111111	9	greensboro	peidmont
6/30/2014	Summer	73	22.77777778	12	greensboro	peidmont
7/1/2014	Summer	77.06	25.03333333	38	greensboro	peidmont
7/2/2014	Summer	78.43	25.79444444	6	greensboro	peidmont
7/3/2014	Summer	71.69	22.05	0	greensboro	peidmont
7/4/2014	Summer	65.05	18.36111111	0	greensboro	peidmont
7/5/2014	Summer	61.16	16.2	0	greensboro	peidmont
7/6/2014	Summer	71.34	21.85555556	19	greensboro	peidmont
7/7/2014	Summer	75.65	24.25	18	greensboro	peidmont
7/8/2014	Summer	71.7	22.05555556	30	greensboro	peidmont
7/9/2014	Summer	70.27	21.26111111	9	greensboro	peidmont
7/10/2014	Summer	70.95	21.63888889	11	greensboro	peidmont
7/11/2014	Summer	68.52	20.28888889	35	greensboro	peidmont
7/12/2014	Summer	73.13	22.85	23	greensboro	peidmont
7/13/2014	Summer	75.31	24.06111111	18	greensboro	peidmont
7/14/2014	Summer	77.25	25.13888889	9	greensboro	peidmont
7/15/2014	Summer	68.78	20.43333333	13	greensboro	peidmont
7/16/2014	Summer	63.99	17.77222222	19	greensboro	peidmont
7/17/2014	Summer	65.14	18.41111111	20	greensboro	peidmont
7/18/2014	Summer	68.36	20.2	20	greensboro	peidmont
7/19/2014	Summer	65.79	18.77222222	11	greensboro	peidmont
7/20/2014	Summer	69.37	20.76111111	20	greensboro	peidmont
7/21/2014	Summer	70.01111111	21.11728395	31	greensboro	peidmont
7/22/2014	Summer	69.8	21	68	greensboro	peidmont
7/23/2014	Summer	74.79	23.77222222	41	greensboro	peidmont
7/24/2014	Summer	67.47	19.70555556	20	greensboro	peidmont

7/25/2014	Summer	69.74	20.96666667	48	greensboro	peidmont
7/26/2014	Summer	73.15	22.86111111	41	greensboro	peidmont
7/27/2014	Summer	77.03636364	25.02020202	0	greensboro	peidmont
7/28/2014	Summer	67.07272727	19.48484848	0	greensboro	peidmont
7/29/2014	Summer	65.00909091	18.33838384	39	greensboro	peidmont
7/30/2014	Summer	65	18.33333333	49	greensboro	peidmont
7/31/2014	Summer	67.07272727	19.48484848	16	greensboro	peidmont
8/1/2014	Summer	62.73636364	17.07575758	7	greensboro	peidmont
8/2/2014	Summer	65.63636364	18.68686869	34	greensboro	peidmont
8/3/2014	Summer	68.68181818	20.37878788	66	greensboro	peidmont
8/4/2014	Summer	67.14545455	19.52525253	96	greensboro	peidmont
8/5/2014	Summer	72.88461538	22.71367521	58	greensboro	peidmont
8/6/2014	Summer	71.25384615	21.80769231	66	greensboro	peidmont
8/7/2014	Summer	68.67692308	20.37606838	68	greensboro	peidmont
8/8/2014	Summer	68.74615385	20.41452991	33	greensboro	peidmont
8/9/2014	Summer	65.73076923	18.73931624	3	greensboro	peidmont
8/10/2014	Summer	70.33076923	21.29487179	24	greensboro	peidmont
8/11/2014	Summer	69.90769231	21.05982906	15	greensboro	peidmont
8/12/2014	Summer	70.02307692	21.12393162	0	greensboro	peidmont
8/13/2014	Summer	64.78461538	18.21367521	37	greensboro	peidmont
8/14/2014	Summer	65.78461538	18.76923077	2	greensboro	peidmont
8/15/2014	Summer	69.66153846	20.92307692	6	greensboro	peidmont
8/16/2014	Summer	70.67692308	21.48717949	4	greensboro	peidmont
8/17/2014	Summer	72.03076923	22.23931624	7	greensboro	peidmont
8/18/2014	Summer	70.16153846	21.2008547	49	greensboro	peidmont
8/19/2014	Summer	71.52307692	21.95726496	61	greensboro	peidmont
8/20/2014	Summer	73.43846154	23.02136752	28	greensboro	peidmont
8/22/2014	Summer	75.22307692	24.01282051	5	greensboro	peidmont
8/23/2014	Summer	72.32307692	22.4017094	2	greensboro	peidmont
8/24/2014	Summer	65.13076923	18.40598291	13	greensboro	peidmont
8/25/2014	Summer	63.07692308	17.26495726	7	greensboro	peidmont
8/26/2014	Summer	64.01538462	17.78632479	4	greensboro	peidmont
8/27/2014	Summer	67.74615385	19.85897436	0	greensboro	peidmont

8/28/2014	Summer	74.37692308	23.54273504	4	greensboro	peidmont
8/29/2014	Summer	73.45384615	23.02991453	3	greensboro	peidmont
8/30/2014	Summer	74.11538462	23.3974359	33	greensboro	peidmont
8/31/2014	Summer	74.33076923	23.51709402	19	greensboro	peidmont
9/22/2012	fall	69.02307692	20.56837607	31	lenoir	Coast
9/23/2012	fall	56.28461538	13.49145299	2	lenoir	Coast
9/24/2012	fall	51.62307692	10.9017094	1	lenoir	Coast
9/25/2012	fall	58.74615385	14.85897436	1	lenoir	Coast
9/26/2012	fall	62.09230769	16.71794872	1	lenoir	Coast
9/27/2012	fall	65.14615385	18.41452991	6	lenoir	Coast
9/28/2012	fall	68.89230769	20.4957265	1	lenoir	Coast
9/29/2012	fall	61.43846154	16.35470085	0	lenoir	Coast
9/30/2012	fall	64.32307692	17.95726496	0	lenoir	Coast
10/1/2012	fall	72.75384615	22.64102564	1	lenoir	Coast
10/2/2012	fall	75.9	24.38888889	0	lenoir	Coast
10/3/2012	fall	72.65384615	22.58547009	0	lenoir	Coast
10/4/2012	fall	65.48461538	18.6025641	1	lenoir	Coast
10/5/2012	fall	61.90769231	16.61538462	1	lenoir	Coast
10/6/2012	fall	66.16923077	18.98290598	2	lenoir	Coast
10/7/2012	fall	54.25384615	12.36324786	1	lenoir	Coast
10/8/2012	fall	52.13076923	11.18376068	0	lenoir	Coast
10/9/2012	fall	55.91538462	13.28632479	0	lenoir	Coast
10/10/2012	fall	53.15384615	11.75213675	0	lenoir	Coast
10/11/2012	fall	47.68461538	8.713675214	0	lenoir	Coast
10/12/2012	fall	51.95384615	11.08547009	1	lenoir	Coast
10/13/2012	fall	49.43846154	9.688034188	0	lenoir	Coast
10/14/2012	fall	62.52307692	16.95726496	2	lenoir	Coast
12/1/2012	Winter	42.25	5.694444444	0	lenoir	Coast
12/2/2012	Winter	53.46428571	11.92460317	0	lenoir	Coast
12/3/2012	Winter	53.95714286	12.1984127	47	lenoir	Coast
12/4/2012	Winter	59.83333333	15.46296296	0	lenoir	Coast
12/5/2012	Winter	50.88666667	10.49259259	0	lenoir	Coast
12/6/2012	Winter	45.83333333	7.685185185	0	lenoir	Coast

12/7/2012	Winter	60.53333333	15.85185185	4	lenoir	Coast
12/8/2012	Winter	53.46	11.92222222	2	lenoir	Coast
12/9/2012	Winter	64.20666667	17.89259259	0	lenoir	Coast
12/10/2012	Winter	64.28666667	17.93703704	2	lenoir	Coast
12/11/2012	Winter	50.75333333	10.41851852	0	lenoir	Coast
12/12/2012	Winter	40.86	4.922222222	0	lenoir	Coast
12/13/2012	Winter	40.58	4.766666667	0	lenoir	Coast
12/14/2012	Winter	36.31333333	2.396296296	0	lenoir	Coast
12/15/2012	Winter	50.08	10.04444444	0	lenoir	Coast
12/16/2012	Winter	57.72	14.28888889	0	lenoir	Coast
12/17/2012	Winter	58.11333333	14.50740741	0	lenoir	Coast
12/18/2012	Winter	45	7.222222222	0	lenoir	Coast
12/19/2012	Winter	44.49333333	6.940740741	0	lenoir	Coast
12/20/2012	Winter	57.78	14.32222222	0	lenoir	Coast
12/21/2012	Winter	39.15333333	3.974074074	0	lenoir	Coast
12/22/2012	Winter	35.53333333	1.962962963	0	lenoir	Coast
12/23/2012	Winter	44.87333333	7.151851852	0	lenoir	Coast
12/24/2012	Winter	48.52	9.177777778	0	lenoir	Coast
12/25/2012	Winter	48.59333333	9.218518519	0	lenoir	Coast
12/26/2012	Winter	49.95333333	9.974074074	0	lenoir	Coast
12/27/2012	Winter	36.58666667	2.548148148	0	lenoir	Coast
12/28/2012	Winter	41.24	5.133333333	0	lenoir	Coast
12/29/2012	Winter	42.12	5.622222222	0	lenoir	Coast
12/30/2012	Winter	31.51333333	-0.27037037	0	lenoir	Coast
12/31/2012	Winter	47.06666667	8.37037037	0	lenoir	Coast
1/1/2013	Winter	51.54	10.85555556	0	lenoir	Coast
1/2/2013	Winter	40.49333333	4.718518519	0	lenoir	Coast
1/3/2013	Winter	33.36666667	0.759259259	0	lenoir	Coast
1/13/2013	Winter	61.95333333	16.64074074	0	lenoir	Coast
1/14/2013	Winter	62.62666667	17.01481481	0	lenoir	Coast
1/15/2013	Winter	53.21333333	11.78518519	0	lenoir	Coast
1/16/2013	Winter	52.01333333	11.11851852	0	lenoir	Coast
1/17/2013	Winter	39.46	4.144444444	0	lenoir	Coast

1/18/2013	Winter	32.78666667	0.437037037	0	lenoir	Coast
1/19/2013	Winter	45.40666667	7.448148148	0	lenoir	Coast
1/20/2013	Winter	40.68	4.822222222	1	lenoir	Coast
1/21/2013	Winter	46.01333333	7.785185185	0	lenoir	Coast
1/22/2013	Winter	26.88	-2.844444444	0	lenoir	Coast
1/23/2013	Winter	33.94666667	1.081481481	0	lenoir	Coast
1/24/2013	Winter	23.65	-4.638888889	0	lenoir	Coast
1/25/2013	Winter	27.87142857	-2.293650794	0	lenoir	Coast
1/26/2013	Winter	28.95	-1.694444444	0	lenoir	Coast
1/27/2013	Winter	30	-1.111111111	0	lenoir	Coast
1/28/2013	Winter	49.77857143	9.876984127	0	lenoir	Coast
1/29/2013	Winter	60.62142857	15.90079365	2	lenoir	Coast
1/30/2013	Winter	63.21428571	17.34126984	0	lenoir	Coast
1/31/2013	Winter	41.42142857	5.234126984	1	lenoir	Coast
2/1/2013	Winter	26.57857143	-3.011904762	1	lenoir	Coast
2/2/2013	Winter	39.94285714	4.412698413	0	lenoir	Coast
2/3/2013	Winter	32.96666667	0.537037037	0	lenoir	Coast
2/4/2013	Winter	41.41428571	5.23015873	0	lenoir	Coast
2/5/2013	Winter	41.84285714	5.468253968	4	lenoir	Coast
2/6/2013	Winter	42.75	5.972222222	5	lenoir	Coast
2/7/2013	Winter	48.04285714	8.912698413	0	lenoir	Coast
2/8/2013	Winter	43.85714286	6.587301587	0	lenoir	Coast
2/9/2013	Winter	32.25714286	0.142857143	1	lenoir	Coast
2/10/2013	Winter	50.22142857	10.12301587	2	lenoir	Coast
2/11/2013	Winter	60.15	15.63888889	6	lenoir	Coast
2/12/2013	Winter	50.29285714	10.16269841	6	lenoir	Coast
2/13/2013	Winter	47.67857143	8.71031746	0	lenoir	Coast
2/14/2013	Winter	37.38571429	2.992063492	0	lenoir	Coast
2/15/2013	Winter	43.61428571	6.452380952	0	lenoir	Coast
2/16/2013	Winter	32.17142857	0.095238095	0	lenoir	Coast
2/17/2013	Winter	26.11428571	-3.26984127	0	lenoir	Coast
2/18/2013	Winter	38.12142857	3.400793651	0	lenoir	Coast
2/19/2013	Winter	46.25714286	7.920634921	4	lenoir	Coast

2/20/2013	Winter	35.92142857	2.178571429	0	lenoir	Coast
2/21/2013	Winter	40.53571429	4.742063492	0	lenoir	Coast
2/22/2013	Winter	41.23571429	5.130952381	0	lenoir	Coast
2/23/2013	Winter	45.25	7.361111111	0	lenoir	Coast
2/24/2013	Winter	42.74285714	5.968253968	0	lenoir	Coast
2/25/2013	Winter	39.25	4.027777778	0	lenoir	Coast
2/26/2013	Winter	57.25714286	14.03174603	5	lenoir	Coast
3/12/2013	Spring	52.55384615	11.41880342	2	lenoir	Coast
3/13/2013	Spring	40.93846154	4.965811966	0	lenoir	Coast
3/14/2013	Spring	34.11538462	1.175213675	0	lenoir	Coast
3/15/2013	Spring	53.33846154	11.85470085	2	lenoir	Coast
3/16/2013	Spring	62.86153846	17.14529915	2	lenoir	Coast
3/17/2013	Spring	43.97692308	6.653846154	2	lenoir	Coast
3/18/2013	Spring	49.43846154	9.688034188	1	lenoir	Coast
3/19/2013	Spring	51.05384615	10.58547009	5	lenoir	Coast
3/20/2013	Spring	40.03076923	4.461538462	0	lenoir	Coast
3/21/2013	Spring	31.68461538	-0.175213675	0	lenoir	Coast
3/22/2013	Spring	39.50769231	4.170940171	0	lenoir	Coast
3/23/2013	Spring	40.23076923	4.572649573	0	lenoir	Coast
3/24/2013	Spring	39.48461538	4.158119658	0	lenoir	Coast
3/25/2013	Spring	39.70769231	4.282051282	0	lenoir	Coast
3/26/2013	Spring	39.6	4.222222222	0	lenoir	Coast
3/27/2013	Spring	39.33846154	4.076923077	0	lenoir	Coast
3/28/2013	Spring	38.15384615	3.418803419	0	lenoir	Coast
3/29/2013	Spring	44.43846154	6.91025641	4	lenoir	Coast
3/30/2013	Spring	50.825	10.45833333	1	lenoir	Coast
3/31/2013	Spring	58.76666667	14.87037037	1	lenoir	Coast
4/1/2013	Spring	51.81666667	11.00925926	1	lenoir	Coast
4/2/2013	Spring	45.54166667	7.523148148	0	lenoir	Coast
4/3/2013	Spring	44.64166667	7.023148148	0	lenoir	Coast
4/4/2013	Spring	44.76666667	7.092592593	0	lenoir	Coast
4/5/2013	Spring	43.35833333	6.310185185	14	lenoir	Coast
4/6/2013	Spring	40.39166667	4.662037037	4	lenoir	Coast

4/7/2013	Spring	55.15	12.86111111	3	lenoir	Coast
4/8/2013	Spring	61.81666667	16.56481481	1	lenoir	Coast
4/9/2013	Spring	65.33333333	18.51851852	2	lenoir	Coast
4/10/2013	Spring	65.73333333	18.74074074	1	lenoir	Coast
4/11/2013	Spring	70.39166667	21.3287037	3	lenoir	Coast
4/12/2013	Spring	64.75833333	18.19907407	1	lenoir	Coast
4/13/2013	Spring	53.30	11.83333333	0	lenoir	Coast
5/7/2013	Spring	56.49090909	13.60606061	5	lenoir	Coast
5/8/2013	Spring	62.94545455	17.19191919	5	lenoir	Coast
5/9/2013	Spring	62.32727273	16.84848485	7	lenoir	Coast
5/10/2013	Spring	70.7	21.5	12	lenoir	Coast
5/11/2013	Spring	71.19090909	21.77272727	1	lenoir	Coast
5/12/2013	Spring	59.19090909	15.10606061	10	lenoir	Coast
5/13/2013	Spring	50.49090909	10.27272727	1	lenoir	Coast
5/14/2013	Spring	58.00909091	14.44949495	5	lenoir	Coast
5/15/2013	Spring	70.14545455	21.19191919	1	lenoir	Coast
5/16/2013	Spring	71.47272727	21.92929293	2	lenoir	Coast
5/17/2013	Spring	68.51818182	20.28787879	6	lenoir	Coast
5/18/2013	Spring	67.15454545	19.53030303	10	lenoir	Coast
5/19/2013	Spring	70.42727273	21.34848485	5	lenoir	Coast
5/20/2013	Spring	69.41818182	20.78787879	4	lenoir	Coast
5/21/2013	Spring	69.45454545	20.80808081	31	lenoir	Coast
5/22/2013	Spring	69.8	21	9	lenoir	Coast
5/23/2013	Spring	66.71818182	19.28787879	2	lenoir	Coast
5/24/2013	Spring	53.4	11.88888889	0	lenoir	Coast
5/25/2013	Spring	53.57272727	11.98484848	1	lenoir	Coast
5/26/2013	Spring	53.42727273	11.90404041	0	lenoir	Coast
5/27/2013	Spring	61.37272727	16.31818182	4	lenoir	Coast
5/28/2013	Spring	67.26363636	19.59090909	6	lenoir	Coast
5/29/2013	Spring	66.08181818	18.93434343	1	lenoir	Coast
5/30/2013	Spring	70.72727273	21.51515152	4	lenoir	Coast
5/31/2013	Spring	71.53636364	21.96464647	1	lenoir	Coast
6/1/2013	Summer	71.09090909	21.71717172	0	lenoir	Coast

6/2/2013	Summer	74.00909091	23.33838384	7	lenoir	Coast
6/3/2013	Summer	69.60909091	20.89393939	0	lenoir	Coast
6/4/2013	Summer	65.64545455	18.69191919	0	lenoir	Coast
6/5/2013	Summer	67.96363636	19.97979798	1	lenoir	Coast
6/6/2013	Summer	71.27272727	21.81818182	1	lenoir	Coast
6/7/2013	Summer	71.76363636	22.09090909	0	lenoir	Coast
6/8/2013	Summer	73.25454545	22.91919192	0	lenoir	Coast
6/9/2013	Summer	74.08181818	23.37878788	1	lenoir	Coast
6/10/2013	Summer	71.28181818	21.82323232	0	lenoir	Coast
6/11/2013	Summer	69.6	20.88888889	0	lenoir	Coast
6/12/2013	Summer	77.26363636	25.14646464	1	lenoir	Coast
6/13/2013	Summer	70.35454545	21.30808081	1	lenoir	Coast
6/14/2013	Summer	60.41818182	15.78787879	0	lenoir	Coast
6/15/2013	Summer	69.47272727	20.81818182	0	lenoir	Coast
6/16/2013	Summer	72.69090909	22.60606061	0	lenoir	Coast
6/17/2013	Summer	72.98181818	22.76767677	5	lenoir	Coast
6/18/2013	Summer	72.34545455	22.41414142	1	lenoir	Coast
6/24/2013	Summer	73.3	22.94444444	2	lenoir	Coast
6/25/2013	Summer	71.97272727	22.20707071	4	lenoir	Coast
6/26/2013	Summer	71.58181818	21.98989899	6	lenoir	Coast
6/27/2013	Summer	73.79090909	23.21717172	0	lenoir	Coast
6/28/2013	Summer	73.93636364	23.2979798	1	lenoir	Coast
6/29/2013	Summer	74.48181818	23.6010101	2	lenoir	Coast
6/30/2013	Summer	76.21818182	24.56565657	6	lenoir	Coast
7/24/2013	Summer	72.90909091	22.72727273	6	lenoir	Coast
7/25/2013	Summer	69.22727273	20.68181818	9	lenoir	Coast
7/26/2013	Summer	68.68181818	20.37878788	6	lenoir	Coast
7/27/2013	Summer	74.30909091	23.50505051	14	lenoir	Coast
7/28/2013	Summer	76.8	24.88888889	8	lenoir	Coast
7/29/2013	Summer	72.73636364	22.63131313	5	lenoir	Coast
9/2/2013	Fall	75.59166667	24.21759259	2	lenoir	Coast
9/3/2013	Fall	68.94166667	20.52314815	1	lenoir	Coast
9/4/2013	Fall	73.5	23.05555556	0	lenoir	Coast

9/5/2013	Fall	68.425	20.23611111	0	lenoir	Coast
9/6/2013	Fall	64.725	18.18055556	2	lenoir	Coast
9/7/2013	Fall	64.98461538	18.32478632	0	lenoir	Coast
9/8/2013	Fall	68.15384615	20.08547009	0	lenoir	Coast
9/9/2013	Fall	70.33846154	21.2991453	0	lenoir	Coast
9/10/2013	Fall	70.59230769	21.44017094	3	lenoir	Coast
9/11/2013	Fall	71.11538462	21.73076923	1	lenoir	Coast
9/12/2013	Fall	74.56923077	23.64957265	1	lenoir	Coast
9/13/2013	Fall	68.40769231	20.22649573	1	lenoir	Coast
9/14/2013	Fall	60.88461538	16.04700855	0	lenoir	Coast
9/15/2013	Fall	65.22307692	18.45726496	0	lenoir	Coast
9/16/2013	Fall	67.36923077	19.64957265	0	lenoir	Coast
9/17/2013	Fall	57.23076923	14.01709402	0	lenoir	Coast
9/18/2013	Fall	55.72307692	13.17948718	1	lenoir	Coast
9/19/2013	Fall	61.73846154	16.52136752	0	lenoir	Coast
9/20/2013	Fall	65.32307692	18.51282051	0	lenoir	Coast
9/21/2013	Fall	70.83846154	21.57692308	1	lenoir	Coast
9/22/2013	Fall	61.72307692	16.51282051	1	lenoir	Coast
9/23/2013	Fall	55.99230769	13.32905983	0	lenoir	Coast
9/24/2013	Fall	57.1	13.94444444	0	lenoir	Coast
9/25/2013	Fall	61.19230769	16.21794872	0	lenoir	Coast
9/26/2013	Fall	61.19230769	16.21794872	0	lenoir	Coast
9/27/2013	Fall	60.45384615	15.80769231	0	lenoir	Coast
9/28/2013	Fall	59.48461538	15.26923077	0	lenoir	Coast
9/29/2013	Fall	59.29230769	15.16239316	0	lenoir	Coast
9/30/2013	Fall	55.23846154	12.91025641	0	lenoir	Coast
10/1/2013	Fall	62.15384615	16.75213675	0	lenoir	Coast
10/2/2013	Fall	63.93846154	17.74358974	0	lenoir	Coast
10/3/2013	Fall	67.78461538	19.88034188	0	lenoir	Coast
10/4/2013	Fall	68.39230769	20.21794872	1	lenoir	Coast
10/5/2013	Fall	69.00769231	20.55982906	1	lenoir	Coast
10/6/2013	Fall	71.77692308	22.0982906	0	lenoir	Coast
10/7/2013	Fall	67.6	19.77777778	0	lenoir	Coast

10/8/2013	Fall	57.48461538	14.15811966	1	lenoir	Coast
10/9/2013	Fall	58.10769231	14.5042735	0	lenoir	Coast
10/10/2013	Fall	58.96153846	14.97863248	1	lenoir	Coast
10/11/2013	Fall	62.87692308	17.15384615	0	lenoir	Coast
10/12/2013	Fall	66.36923077	19.09401709	4	lenoir	Coast
10/13/2013	Fall	67.46153846	19.7008547	0	lenoir	Coast
10/14/2013	Fall	61.00769231	16.11538462	0	lenoir	Coast
10/15/2013	Fall	64.66923077	18.14957265	1	lenoir	Coast
10/16/2013	Fall	61.51538462	16.3974359	0	lenoir	Coast
10/17/2013	Fall	68.31538462	20.17521368	0	lenoir	Coast
10/18/2013	Fall	64.34615385	17.97008547	0	lenoir	Coast
10/19/2013	Fall	64.92307692	18.29059829	0	lenoir	Coast
10/20/2013	Fall	50.07142857	10.03968254	0	lenoir	Coast
10/21/2013	Fall	55.42142857	13.01190476	0	lenoir	Coast
10/22/2013	Fall	59.99285714	15.5515873	0	lenoir	Coast
10/23/2013	Fall	50.08571429	10.04761905	0	lenoir	Coast
10/24/2013	Fall	45.85714286	7.698412698	0	lenoir	Coast
10/25/2013	Fall	37.47142857	3.03968254	0	lenoir	Coast
10/26/2013	Fall	44.92142857	7.178571429	0	lenoir	Coast
10/27/2013	Fall	47.67142857	8.706349206	0	lenoir	Coast
10/28/2013	Fall	54.25	12.36111111	1	lenoir	Coast
10/29/2013	Fall	55.16428571	12.86904762	0	lenoir	Coast
10/30/2013	Fall	59.8	15.44444444	0	lenoir	Coast
10/31/2013	Fall	70.08666667	21.15925926	0	lenoir	Coast
11/1/2013	Fall	68.28	20.15555556	0	lenoir	Coast
11/2/2013	Fall	55.32857143	12.96031746	1	lenoir	Coast
11/13/2013	Fall	30.48666667	-0.840740741	0	lenoir	Coast
11/14/2013	Fall	36.86	2.7	0	lenoir	Coast
11/15/2013	Fall	58.30666667	14.61481481	0	lenoir	Coast
11/16/2013	Fall	56.17333333	13.42962963	0	lenoir	Coast
11/17/2013	Fall	69.03333333	20.57407407	0	lenoir	Coast
11/18/2013	Fall	56.26666667	13.48148148	0	lenoir	Coast
11/19/2013	Fall	42.84666667	6.025925926	0	lenoir	Coast

11/20/2013	Fall	47.61333333	8.674074074	0	lenoir	Coast
11/21/2013	Fall	54.94	12.74444444	0	lenoir	Coast
11/22/2013	Fall	63.78666667	17.65925926	1	lenoir	Coast
11/23/2013	Fall	46.88666667	8.27037037	0	lenoir	Coast
11/24/2013	Fall	27.71333333	-2.381481481	0	lenoir	Coast
11/25/2013	Fall	34.8	1.555555556	0	lenoir	Coast
11/26/2013	Fall	66.16	18.97777778	0	lenoir	Coast
11/27/2013	Fall	34.45333333	1.362962963	0	lenoir	Coast
11/28/2013	Fall	30.77333333	-0.681481481	0	lenoir	Coast
11/29/2013	Fall	37.18666667	2.881481481	0	lenoir	Coast
11/30/2013	Fall	43.56	6.422222222	0	lenoir	Coast
12/1/2013	Winter	42.24666667	5.692592593	0	lenoir	Coast
12/2/2013	Winter	40.05333333	4.474074074	0	lenoir	Coast
12/3/2013	Winter	52.58666667	11.43703704	0	lenoir	Coast
12/4/2013	Winter	57.80666667	14.33703704	0	lenoir	Coast
12/7/2013	Winter	43.12666667	6.181481481	0	lenoir	Coast
12/8/2013	Winter	38.04666667	3.359259259	0	lenoir	Coast
12/9/2013	Winter	52.23333333	11.24074074	0	lenoir	Coast
12/10/2013	Winter	35.16	1.755555556	0	lenoir	Coast
12/11/2013	Winter	35.56	1.977777778	0	lenoir	Coast
12/12/2013	Winter	29.64	-1.311111111	0	lenoir	Coast
12/13/2013	Winter	38.67333333	3.707407407	0	lenoir	Coast
12/14/2013	Winter	56.76	13.75555556	0	lenoir	Coast
12/15/2013	Winter	40.5	4.722222222	0	lenoir	Coast
12/16/2013	Winter	41.37333333	5.207407407	0	lenoir	Coast
12/19/2013	Winter	45.39333333	7.440740741	0	lenoir	Coast
12/20/2013	Winter	56.71333333	13.72962963	1	lenoir	Coast
12/23/2013	Winter	54.18	12.32222222	0	lenoir	Coast
12/24/2013	Winter	35.66666667	2.037037037	0	lenoir	Coast
12/25/2013	Winter	34.2	1.222222222	0	lenoir	Coast
12/26/2013	Winter	34.67333333	1.485185185	0	lenoir	Coast
12/27/2013	Winter	33.38666667	0.77037037	0	lenoir	Coast
12/28/2013	Winter	49.54666667	9.748148148	1	lenoir	Coast

12/29/2013	Winter	51.5	10.83333333	0	lenoir	Coast
12/30/2013	Winter	46.11333333	7.840740741	0	lenoir	Coast
12/31/2013	Winter	38.52	3.622222222	0	lenoir	Coast
1/1/2014	Winter	46.11333333	7.840740741	0	lenoir	Coast
1/2/2014	Winter	45.65333333	7.585185185	0	lenoir	Coast
1/3/2014	Winter	26.69333333	-2.948148148	0	lenoir	Coast
1/4/2014	Winter	39.15333333	3.974074074	0	lenoir	Coast
1/5/2014	Winter	63.06666667	17.25925926	0	lenoir	Coast
1/6/2014	Winter	29.3	-1.5	0	lenoir	Coast
1/7/2014	Winter	21	-6.111111111	0	lenoir	Coast
1/8/2014	Winter	28.13333333	-2.148148148	0	lenoir	Coast
1/9/2014	Winter	43.88	6.6	0	lenoir	Coast
1/10/2014	Winter	63.26	17.36666667	5	lenoir	Coast
1/11/2014	Winter	55.8	13.22222222	0	lenoir	Coast
1/12/2014	Winter	41.45333333	5.251851852	0	lenoir	Coast
1/13/2014	Winter	55.24	12.91111111	1	lenoir	Coast
1/14/2014	Winter	41.37333333	5.207407407	0	lenoir	Coast
1/15/2014	Winter	44.1	6.722222222	0	lenoir	Coast
1/16/2014	Winter	32.90666667	0.503703704	0	lenoir	Coast
1/17/2014	Winter	47.14666667	8.414814815	0	lenoir	Coast
1/18/2014	Winter	31.18	-0.455555556	0	lenoir	Coast
1/19/2014	Winter	40.57333333	4.762962963	0	lenoir	Coast
1/20/2014	Winter	45.92	7.733333333	0	lenoir	Coast
1/21/2014	Winter	33.24666667	0.692592593	0	lenoir	Coast
1/22/2014	Winter	22.31333333	-5.381481481	0	lenoir	Coast
1/23/2014	Winter	26.97333333	-2.792592593	0	lenoir	Coast
1/24/2014	Winter	19.6	-6.888888889	0	lenoir	Coast
1/25/2014	Winter	36.95	2.75	0	lenoir	Coast
1/26/2014	Winter	43.24285714	6.246031746	0	lenoir	Coast
1/27/2014	Winter	42.61428571	5.896825397	0	lenoir	Coast
2/25/2014	Winter	39.40714286	4.115079365	0	lenoir	Coast
2/26/2014	Winter	31.97857143	-0.011904762	0	lenoir	Coast
2/27/2014	Winter	35.49285714	1.94047619	0	lenoir	Coast

2/28/2014	Winter	32.93571429	0.51984127	0	lenoir	Coast
3/1/2014	Spring	36.7	2.611111111	0	lenoir	Coast
3/2/2014	Spring	56.98461538	13.88034188	0	lenoir	Coast
3/3/2014	Spring	24.2	-4.333333333	0	lenoir	Coast
3/4/2014	Spring	31.88571429	-0.063492063	0	lenoir	Coast
3/5/2014	Spring	35.96	2.2	0	lenoir	Coast
3/6/2014	Spring	38.54666667	3.637037037	0	lenoir	Coast
3/7/2014	Spring	37.24	2.911111111	0	lenoir	Coast
3/8/2014	Spring	50.51875	10.28819444	0	lenoir	Coast
4/1/2014	Spring	54.55	12.52777778	0	lenoir	Coast
4/2/2014	Spring	62.05833333	16.69907407	1	lenoir	Coast
4/3/2014	Spring	64.625	18.125	1	lenoir	Coast
4/4/2014	Spring	70.37272727	21.31818182	1	lenoir	Coast
4/5/2014	Spring	53.66666667	12.03703704	2	lenoir	Coast
4/6/2014	Spring	53.14545455	11.74747475	0	lenoir	Coast
4/7/2014	Spring	66.28	19.04444444	1	lenoir	Coast
4/8/2014	Spring	53.52727273	11.95959596	0	lenoir	Coast
4/9/2014	Spring	44.71818182	7.065656566	0	lenoir	Coast
4/10/2014	Spring	57.65454545	14.25252525	0	lenoir	Coast
4/11/2014	Spring	61.54545455	16.41414141	1	lenoir	Coast
4/12/2014	Spring	61.16666667	16.2037037	0	lenoir	Coast
4/13/2014	Spring	62.98181818	17.21212121	1	lenoir	Coast
4/14/2014	Spring	70	21.11111111	3	lenoir	Coast
4/15/2014	Spring	43.11666667	6.175925926	0	lenoir	Coast
4/16/2014	Spring	40.06666667	4.481481481	0	lenoir	Coast
4/17/2014	Spring	44.45	6.916666667	0	lenoir	Coast
4/18/2014	Spring	49.51818182	9.732323232	0	lenoir	Coast
4/19/2014	Spring	49.55454545	9.752525253	0	lenoir	Coast
4/20/2014	Spring	50.45833333	10.25462963	0	lenoir	Coast
4/21/2014	Spring	48.63333333	9.240740741	1	lenoir	Coast
4/22/2014	Spring	65.38333333	18.5462963	0	lenoir	Coast
4/24/2014	Spring	53.59090909	11.99494949	22	lenoir	Coast
4/25/2014	Spring	61.85555556	16.58641975	6	lenoir	Coast

4/26/2014	Spring	60.4	15.77777778	30	lenoir	Coast
4/27/2014	Spring	57.84545455	14.35858586	14	lenoir	Coast
4/28/2014	Spring	58.62727273	14.79292929	19	lenoir	Coast
4/29/2014	Spring	70.9	21.61111111	20	lenoir	Coast
4/30/2014	Spring	69.22	20.67777778	36	lenoir	Coast
5/1/2014	Spring	66.78181818	19.32323232	1	lenoir	Coast
5/2/2014	Spring	54.87	12.70555556	7	lenoir	Coast
5/3/2014	Spring	57.13	13.96111111	0	lenoir	Coast
5/4/2014	Spring	63.08	17.26666667	16	lenoir	Coast
5/5/2014	Spring	62.53636364	16.96464646	0	lenoir	Coast
5/6/2014	Spring	64.83636364	18.24242424	20	lenoir	Coast
5/7/2014	Spring	65.37	18.53888889	10	lenoir	Coast
5/8/2014	Spring	69.51	20.83888889	0	lenoir	Coast
5/9/2014	Spring	72.48	22.48888889	33	lenoir	Coast
5/10/2014	Spring	70.71	21.50555556	17	lenoir	Coast
5/11/2014	Spring	67.93636364	19.96464646	31	lenoir	Coast
5/12/2014	Spring	71.38181818	21.87878788	0	lenoir	Coast
5/13/2014	Spring	71.85454545	22.14141414	80	lenoir	Coast
5/14/2014	Spring	69.89090909	21.05050505	0	lenoir	Coast
5/15/2014	Spring	72.01	22.22777778	30	lenoir	Coast
5/16/2014	Spring	57.18	13.98888889	0	lenoir	Coast
5/17/2014	Spring	56.31	13.50555556	41	lenoir	Coast
5/18/2014	Spring	51.83636364	11.02020202	3	lenoir	Coast
5/19/2014	Spring	52.03636364	11.13131313	1	lenoir	Coast
5/20/2014	Spring	64.69090909	18.16161616	17	lenoir	Coast
5/21/2014	Spring	71.40909091	21.89393939	0	lenoir	Coast
5/22/2014	Spring	71.73	22.07222222	3	lenoir	Coast
5/23/2014	Spring	67.12	19.51111111	4	lenoir	Coast
5/24/2014	Spring	59.14	15.07777778	5	lenoir	Coast
5/25/2014	Spring	64.72727273	18.18181818	11	lenoir	Coast
5/26/2014	Spring	71.95454545	22.1969697	5	lenoir	Coast
5/27/2014	Spring	72.15454545	22.30808081	0	lenoir	Coast
5/28/2014	Spring	72.50909091	22.50505051	2	lenoir	Coast

5/29/2014	Spring	67.66363636	19.81313131	0	lenoir	Coast
5/30/2014	Spring	64.88	18.26666667	0	lenoir	Coast
5/31/2014	Spring	57.97	14.42777778	0	lenoir	Coast
6/1/2014	Summer	54.38181818	12.43434343	0	lenoir	Coast
6/2/2014	Summer	63.40909091	17.44949495	1	lenoir	Coast
6/3/2014	Summer	72.12727273	22.29292929	3	lenoir	Coast
6/6/2014	Summer	67.01818182	19.45454545	0	lenoir	Coast
6/7/2014	Summer	66.05454545	18.91919192	0	lenoir	Coast
6/8/2014	Summer	70.5	21.38888889	0	lenoir	Coast
6/9/2014	Summer	73.16363636	22.86868687	0	lenoir	Coast
6/10/2014	Summer	78.39090909	25.77272727	2	lenoir	Coast
6/11/2014	Summer	75.48181818	24.15656566	1	lenoir	Coast
6/12/2014	Summer	69.8	21	0	lenoir	Coast
6/13/2014	Summer	71.27272727	21.81818182	0	lenoir	Coast
6/14/2014	Summer	71.75454545	22.08585859	0	lenoir	Coast
6/15/2014	Summer	70.9	21.61111111	0	lenoir	Coast
6/16/2014	Summer	74.82727273	23.79292929	1	lenoir	Coast
6/17/2014	Summer	75.56363636	24.2020202	3	lenoir	Coast
6/18/2014	Summer	78.25454545	25.6969697	0	lenoir	Coast
6/19/2014	Summer	71.77272727	22.0959596	0	lenoir	Coast
6/20/2014	Summer	72.14545455	22.3030303	2	lenoir	Coast
6/21/2014	Summer	71.05454545	21.6969697	1	lenoir	Coast
6/22/2014	Summer	69.59090909	20.88383838	1	lenoir	Coast
6/23/2014	Summer	69.96363636	21.09090909	2	lenoir	Coast
6/24/2014	Summer	74.61818182	23.67676768	0	lenoir	Coast
6/25/2014	Summer	78.00909091	25.56060606	2	lenoir	Coast
6/26/2014	Summer	75.70909091	24.28282828	2	lenoir	Coast
6/27/2014	Summer	71.98181818	22.21212121	4	lenoir	Coast
6/28/2014	Summer	67.4	19.66666667	5	lenoir	Coast
6/29/2014	Summer	67.6	19.77777778	3	lenoir	Coast
6/30/2014	Summer	71.33636364	21.85353535	3	lenoir	Coast
7/1/2014	Summer	76.44545455	24.69191919	1	lenoir	Coast
7/2/2014	Summer	78.42727273	25.79292929	1	lenoir	Coast

7/3/2014	Summer	74.51818182	23.62121212	1	lenoir	Coast
7/4/2014	Summer	70.22727273	21.23737374	1	lenoir	Coast
7/5/2014	Summer	64.79090909	18.21717172	3	lenoir	Coast
7/6/2014	Summer	70.78181818	21.54545455	0	lenoir	Coast
7/7/2014	Summer	76.79090909	24.88383838	4	lenoir	Coast
7/8/2014	Summer	78.56363636	25.86868687	6	lenoir	Coast
7/9/2014	Summer	78.06363636	25.59090909	3	lenoir	Coast
7/10/2014	Summer	71.69090909	22.05050505	0	lenoir	Coast
7/11/2014	Summer	72.2	22.33333333	0	lenoir	Coast
7/12/2014	Summer	73.3	22.94444444	6	lenoir	Coast
7/13/2014	Summer	75.99090909	24.43939394	15	lenoir	Coast
7/14/2014	Summer	78.81818182	26.01010101	6	lenoir	Coast
7/15/2014	Summer	73.47272727	23.04040404	4	lenoir	Coast
7/16/2014	Summer	68.80909091	20.44949495	8	lenoir	Coast
7/17/2014	Summer	68.65454545	20.36363636	4	lenoir	Coast
7/18/2014	Summer	67.56363636	19.75757576	5	lenoir	Coast
7/19/2014	Summer	71.72727273	22.07070707	41	lenoir	Coast
7/20/2014	Summer	74.55454545	23.64141414	31	lenoir	Coast
7/21/2014	Summer	73.14545455	22.85858586	99	lenoir	Coast
7/22/2014	Summer	75.57272727	24.20707071	139	lenoir	Coast
7/23/2014	Summer	77.22727273	25.12626263	32	lenoir	Coast
7/24/2014	Summer	68.70909091	20.39393939	8	lenoir	Coast
7/25/2014	Summer	73.29090909	22.93939394	118	lenoir	Coast
7/26/2014	Summer	77.09090909	25.05050505	75	lenoir	Coast
7/27/2014	Summer	80.47272727	26.92929293	13	lenoir	Coast
7/28/2014	Summer	70.76363636	21.53535354	8	lenoir	Coast
7/29/2014	Summer	66.75454545	19.30808081	35	lenoir	Coast
7/30/2014	Summer	69.33636364	20.74242424	65	lenoir	Coast
7/31/2014	Summer	71.8	22.11111111	45	lenoir	Coast
8/1/2014	Summer	71.52727273	21.95959596	20	lenoir	Coast
8/2/2014	Summer	72.19090909	22.32828283	20	lenoir	Coast
8/3/2014	Summer	70.96363636	21.64646465	18	lenoir	Coast
8/4/2014	Summer	72.47272727	22.48484848	9	lenoir	Coast

8/5/2014	Summer	70.80909091	21.56060606	18	lenoir	Coast
8/6/2014	Summer	72.23636364	22.35353535	23	lenoir	Coast
8/7/2014	Summer	70.19090909	21.21717172	8	lenoir	Coast
8/8/2014	Summer	72.20909091	22.33838384	4	lenoir	Coast
8/9/2014	Summer	68.85454545	20.47474747	11	lenoir	Coast
8/10/2014	Summer	71.77272727	22.0959596	6	lenoir	Coast
8/11/2014	Summer	72.49090909	22.49494949	12	lenoir	Coast
8/12/2014	Summer	73.64545455	23.13636364	20	lenoir	Coast
8/13/2014	Summer	66.23636364	19.02020202	3	lenoir	Coast
8/14/2014	Summer	64.44545455	18.02525253	15	lenoir	Coast
8/15/2014	Summer	71.24166667	21.80092593	23	lenoir	Coast
9/30/2012	fall	65.31538462	18.50854701	16	North River	Coast
10/1/2012	fall	73.98461538	23.32478632	9	North River	Coast
10/2/2012	fall	77.8	25.44444444	11	North River	Coast
10/3/2012	fall	75.97692308	24.43162393	8	North River	Coast
10/4/2012	fall	69.23076923	20.68376068	18	North River	Coast
10/5/2012	fall	64.84615385	18.24786325	12	North River	Coast
10/6/2012	fall	69.88461538	21.04700854	19	North River	Coast
10/7/2012	fall	59.29230769	15.16239316	226	North River	Coast
10/8/2012	fall	62.49230769	16.94017094	38	North River	Coast
10/9/2012	fall	61.81538462	16.56410257	16	North River	Coast
10/10/2012	fall	59.71538462	15.3974359	38	North River	Coast
10/11/2012	fall	50.3	10.16666667	15	North River	Coast
10/12/2012	fall	57.56153846	14.2008547	103	North River	Coast
10/13/2012	fall	52.70769231	11.50427351	17	North River	Coast
10/14/2012	fall	66.48461538	19.15811966	38	North River	Coast
10/15/2012	fall	63.77692308	17.65384616	58	North River	Coast
10/16/2012	fall	50.6	10.33333333	33	North River	Coast
10/17/2012	fall	59.16153846	15.08974359	18	North River	Coast
10/18/2012	fall	70.93846154	21.63247863	21	North River	Coast
10/19/2012	fall	62.03076923	16.68376068	25	North River	Coast
10/20/2012	fall	58.04285714	14.46825397	61	North River	Coast
10/21/2012	fall	50.41428571	10.23015873	8	North River	Coast

10/22/2012	fall	52.95	11.63888889	9	North River	Coast
12/1/2012	Winter	42.49285714	5.829365078	3	North River	Coast
12/2/2012	Winter	56.31428571	13.50793651	17	North River	Coast
12/3/2012	Winter	54.73333333	12.62962963	31	North River	Coast
12/4/2012	Winter	61.54	16.41111111	192	North River	Coast
12/5/2012	Winter	48.54666667	9.192592594	12	North River	Coast
12/6/2012	Winter	44.80666667	7.114814817	0	North River	Coast
12/7/2012	Winter	63.19333333	17.32962963	33	North River	Coast
12/8/2012	Winter	54.5	12.5	173	North River	Coast
12/9/2012	Winter	64.21333333	17.89629629	131	North River	Coast
12/10/2012	Winter	66.06	18.92222222	161	North River	Coast
12/11/2012	Winter	52.36	11.31111111	1	North River	Coast
12/12/2012	Winter	48.49333333	9.162962961	5	North River	Coast
12/13/2012	Winter	44.82666667	7.125925928	1	North River	Coast
12/14/2012	Winter	39.44666667	4.137037039	1	North River	Coast
12/15/2012	Winter	52.48	11.37777778	5	North River	Coast
12/16/2012	Winter	59.11333333	15.06296296	25	North River	Coast
12/17/2012	Winter	60.36	15.75555556	27	North River	Coast
12/18/2012	Winter	48.46666667	9.14814815	5	North River	Coast
12/19/2012	Winter	48.17333333	8.985185183	1	North River	Coast
12/20/2012	Winter	60.7	15.94444444	13	North River	Coast
12/21/2012	Winter	40.60666667	4.781481483	0	North River	Coast
12/22/2012	Winter	38.70666667	3.725925928	1	North River	Coast
12/23/2012	Winter	47.22	8.455555556	1	North River	Coast
12/24/2012	Winter	51.58666667	10.88148148	73	North River	Coast
12/25/2012	Winter	49.46666667	9.703703706	1	North River	Coast
12/26/2012	Winter	53.72666667	12.07037037	54	North River	Coast
12/27/2012	Winter	39.38	4.1	0	North River	Coast
12/28/2012	Winter	44.23333333	6.796296294	0	North River	Coast
12/29/2012	Winter	42.58	5.877777778	1	North River	Coast
12/30/2012	Winter	34.93333333	1.629629628	0	North River	Coast
12/31/2012	Winter	48.21333333	9.007407406	1	North River	Coast
1/1/2013	Winter	47.96	8.866666667	1	North River	Coast

1/2/2013	Winter	41.06	5.033333333	0	North River	Coast
1/3/2013	Winter	34.12	1.177777778	0	North River	Coast
1/4/2013	Winter	39.02	3.9	0	North River	Coast
1/5/2013	Winter	43.01333333	6.118518517	0	North River	Coast
1/6/2013	Winter	43.71333333	6.507407406	3	North River	Coast
1/7/2013	Winter	36.58666667	2.54814815	0	North River	Coast
1/8/2013	Winter	44.94	7.188888889	7	North River	Coast
1/12/2013	Winter	55.2	12.88888889	28	North River	Coast
1/13/2013	Winter	60.36	15.75555556	73	North River	Coast
1/14/2013	Winter	58.45333333	14.69629629	112	North River	Coast
1/15/2013	Winter	52.10666667	11.17037037	2	North River	Coast
1/16/2013	Winter	53.15333333	11.75185185	2	North River	Coast
1/17/2013	Winter	41.38	5.211111111	0	North River	Coast
1/18/2013	Winter	33.84666667	1.025925928	0	North River	Coast
1/19/2013	Winter	48.28666667	9.04814815	1	North River	Coast
1/20/2013	Winter	46.6	8.111111111	15	North River	Coast
1/21/2013	Winter	45.05333333	7.25185185	3	North River	Coast
1/22/2013	Winter	27.69333333	-2.392592594	1	North River	Coast
1/23/2013	Winter	34.46666667	1.370370372	0	North River	Coast
1/24/2013	Winter	25.48	-3.622222222	0	North River	Coast
1/25/2013	Winter	29.82666667	-1.207407406	0	North River	Coast
1/26/2013	Winter	31.32142857	-0.376984128	0	North River	Coast
1/27/2013	Winter	33.58571429	0.880952383	0	North River	Coast
1/28/2013	Winter	52.19285714	11.21825397	0	North River	Coast
1/29/2013	Winter	60.31428571	15.73015873	17	North River	Coast
1/30/2013	Winter	66.74285714	19.3015873	32	North River	Coast
1/31/2013	Winter	42.22142857	5.678571428	3	North River	Coast
2/1/2013	Winter	27.22857143	-2.65079365	3	North River	Coast
2/2/2013	Winter	40.05	4.472222222	0	North River	Coast
2/3/2013	Winter	36.64285714	2.579365078	1	North River	Coast
2/4/2013	Winter	43.67857143	6.488095239	0	North River	Coast
2/5/2013	Winter	42.92142857	6.067460317	6	North River	Coast
2/6/2013	Winter	43.92142857	6.623015872	5	North River	Coast

2/7/2013	Winter	51.69285714	10.94047619	2	North River	Coast
2/8/2013	Winter	43.97142857	6.65079365	2	North River	Coast
2/9/2013	Winter	38.85	3.805555556	0	North River	Coast
2/10/2013	Winter	49.43571429	9.686507939	0	North River	Coast
2/11/2013	Winter	61.91428571	16.61904762	101	North River	Coast
2/12/2013	Winter	51.96428571	11.09126984	93	North River	Coast
2/13/2013	Winter	48.10714286	8.9484127	3	North River	Coast
2/14/2013	Winter	39.42857143	4.126984128	1	North River	Coast
2/15/2013	Winter	45.50714286	7.503968256	9	North River	Coast
2/16/2013	Winter	34.56428571	1.424603172	0	North River	Coast
2/17/2013	Winter	26.17142857	-3.238095239	0	North River	Coast
2/18/2013	Winter	40.70714286	4.837301589	0	North River	Coast
2/19/2013	Winter	48.79285714	9.329365078	7	North River	Coast
2/20/2013	Winter	37.71428571	3.174603172	0	North River	Coast
2/21/2013	Winter	40.52857143	4.738095239	0	North River	Coast
2/22/2013	Winter	49.50714286	9.726190478	1	North River	Coast
2/23/2013	Winter	47.1	8.388888889	0	North River	Coast
2/24/2013	Winter	41.47857143	5.265873017	2	North River	Coast
2/25/2013	Winter	41	5	3	North River	Coast
2/26/2013	Winter	60.2	15.66666667	96	North River	Coast
2/27/2013	Winter	49.35714286	9.642857144	76	North River	Coast
2/28/2013	Winter	40.63571429	4.79761905	0	North River	Coast
3/1/2013	Spring	44.23571429	6.79761905	0	North River	Coast
3/2/2013	Spring	37.50714286	3.059523811	1	North River	Coast
3/3/2013	Spring	37.52857143	3.071428572	0	North River	Coast
3/4/2013	Spring	38.075	3.375	0	North River	Coast
3/5/2013	Spring	49.8	9.888888889	0	North River	Coast
3/6/2013	Spring	42.77142857	5.984126983	0	North River	Coast
3/7/2013	Spring	42.12142857	5.623015872	3	North River	Coast
3/8/2013	Spring	44.25714286	6.809523811	0	North River	Coast
3/12/2013	Spring	49.86923077	9.927350428	67	North River	Coast
3/13/2013	Spring	39.74615385	4.303418806	5	North River	Coast
3/14/2013	Spring	35.97692308	2.209401711	0	North River	Coast

3/15/2013	Spring	50.40769231	10.22649573	4	North River	Coast
3/16/2013	Spring	52.52307692	11.4017094	71	North River	Coast
3/17/2013	Spring	41.66153846	5.367521367	1	North River	Coast
3/18/2013	Spring	48.53846154	9.188034189	15	North River	Coast
3/19/2013	Spring	46.39230769	7.995726494	34	North River	Coast
3/20/2013	Spring	38.02307692	3.346153844	2	North River	Coast
3/21/2013	Spring	27.24615385	-2.641025639	3	North River	Coast
3/22/2013	Spring	36.3	2.388888889	0	North River	Coast
3/23/2013	Spring	39.15384615	3.974358972	0	North River	Coast
3/24/2013	Spring	39.09230769	3.940170939	1	North River	Coast
3/27/2013	Spring	37.30769231	2.94871795	3	North River	Coast
3/28/2013	Spring	38.16923077	3.427350428	2	North River	Coast
3/29/2013	Spring	40.66666667	4.814814817	4	North River	Coast
3/30/2013	Spring	49.5	9.722222222	26	North River	Coast
3/31/2013	Spring	57.39166667	14.10648148	47	North River	Coast
4/1/2013	Spring	50.675	10.375	62	North River	Coast
4/2/2013	Spring	44.01666667	6.675925926	5	North River	Coast
4/3/2013	Spring	42.775	5.986111111	5	North River	Coast
4/4/2013	Spring	46.78181818	8.212121212	1	North River	Coast
4/5/2013	Spring	43.90833333	6.615740741	6	North River	Coast
4/6/2013	Spring	41.85833333	5.476851852	0	North River	Coast
4/7/2013	Spring	56	13.33333333	150	North River	Coast
4/8/2013	Spring	62.28333333	16.82407407	329	North River	Coast
4/9/2013	Spring	65.40833333	18.56018519	286	North River	Coast
4/10/2013	Spring	65.99166667	18.88425926	361	North River	Coast
4/11/2013	Spring	69.88333333	21.0462963	414	North River	Coast
4/12/2013	Spring	64.25833333	17.9212963	373	North River	Coast
4/13/2013	Spring	52.56666667	11.42592593	86	North River	Coast
4/14/2013	Spring	60.40909091	15.78282828	741	North River	Coast
4/15/2013	Spring	62.26666667	16.81481481	533	North River	Coast
4/16/2013	Spring	59.575	15.31944444	203	North River	Coast
4/17/2013	Spring	64.575	18.09722222	156	North River	Coast
4/18/2013	Spring	66.55833333	19.19907407	130	North River	Coast

4/19/2013	Spring	65.30833333	18.50462963	148	North River	Coast
4/20/2013	Spring	50.09166667	10.05092593	19	North River	Coast
4/21/2013	Spring	46.84545455	8.247474747	3	North River	Coast
4/22/2013	Spring	50.9	10.5	3	North River	Coast
4/23/2013	Spring	45.99090909	7.772727273	23	North River	Coast
4/24/2013	Spring	63.70909091	17.61616162	148	North River	Coast
4/25/2013	Spring	45.34545455	7.414141414	28	North River	Coast
4/26/2013	Spring	49.70909091	9.838383838	153	North River	Coast
4/27/2013	Spring	51.19090909	10.66161616	313	North River	Coast
4/28/2013	Spring	60.69090909	15.93939394	236	North River	Coast
4/29/2013	Spring	58.7	14.83333333	243	North River	Coast
4/30/2013	Spring	59.08181818	15.04545455	387	North River	Coast
5/1/2013	Spring	56.96363636	13.86868687	287	North River	Coast
5/2/2013	Spring	53.02727273	11.68181818	424	North River	Coast
5/3/2013	Spring	52.2	11.22222222	154	North River	Coast
5/4/2013	Spring	51.52727273	10.84848485	59	North River	Coast
5/5/2013	Spring	57.97272727	14.42929293	481	North River	Coast
5/6/2013	Spring	63.45454545	17.47474747	628	North River	Coast
5/7/2013	Spring	57.69090909	14.27272727	363	North River	Coast
5/8/2013	Spring	62.89090909	17.16161616	511	North River	Coast
5/9/2013	Spring	63.9	17.72222222	256	North River	Coast
5/10/2013	Spring	70.55454545	21.41919192	446	North River	Coast
5/11/2013	Spring	69.58181818	20.87878788	411	North River	Coast
5/12/2013	Spring	58.43636364	14.68686869	309	North River	Coast
5/13/2013	Spring	47.81818182	8.787878788	51	North River	Coast
5/14/2013	Spring	61.4	16.33333333	542	North River	Coast
5/15/2013	Spring	70.56363636	21.42424242	757	North River	Coast
5/16/2013	Spring	69.93636364	21.07575758	433	North River	Coast
5/17/2013	Spring	66.89090909	19.38383838	382	North River	Coast
5/18/2013	Spring	67.7	19.83333333	224	North River	Coast
5/19/2013	Spring	70.69090909	21.49494949	193	North River	Coast
5/20/2013	Spring	70.31818182	21.28787879	109	North River	Coast
5/21/2013	Spring	71.77272727	22.0959596	149	North River	Coast

5/22/2013	Spring	72.80909091	22.67171717	96	North River	Coast
5/23/2013	Spring	68.66363636	20.36868687	46	North River	Coast
5/24/2013	Spring	50.07272727	10.04040404	43	North River	Coast
5/25/2013	Spring	49.80909091	9.893939394	27	North River	Coast
5/26/2013	Spring	54.11818182	12.28787879	105	North River	Coast
5/27/2013	Spring	62.96363636	17.2020202	163	North River	Coast
5/28/2013	Spring	68.63636364	20.35353535	237	North River	Coast
5/29/2013	Spring	69.34545455	20.74747475	432	North River	Coast
5/30/2013	Spring	70.79090909	21.55050505	243	North River	Coast
5/31/2013	Spring	71.80909091	22.11616162	184	North River	Coast
6/1/2013	Summer	72.89090909	22.71717172	216	North River	Coast
6/2/2013	Summer	74.6	23.66666667	199	North River	Coast
6/3/2013	Summer	69.40909091	20.78282828	106	North River	Coast
6/4/2013	Summer	57.28181818	14.04545455	120	North River	Coast
6/5/2013	Summer	59.55454545	15.30808081	102	North River	Coast
6/6/2013	Summer	71.20909091	21.78282828	168	North River	Coast
6/7/2013	Summer	71.67272727	22.04040404	416	North River	Coast
6/21/2013	Summer	66.97272727	19.42929293	173	North River	Coast
6/22/2013	Summer	73.23636364	22.90909091	153	North River	Coast
6/23/2013	Summer	73.32727273	22.95959596	152	North River	Coast
6/24/2013	Summer	73.74545455	23.19191919	150	North River	Coast
6/25/2013	Summer	73.46363636	23.03535354	155	North River	Coast
6/26/2013	Summer	73.56363636	23.09090909	301	North River	Coast
6/27/2013	Summer	76.9	24.94444444	325	North River	Coast
6/28/2013	Summer	72.66363636	22.59090909	207	North River	Coast
6/29/2013	Summer	76.00909091	24.44949495	189	North River	Coast
6/30/2013	Summer	77.58181818	25.32323232	264	North River	Coast
7/2/2013	Summer	75.02727273	23.9040404	61	North River	Coast
7/3/2013	Summer	75.51818182	24.17676768	299	North River	Coast
7/4/2013	Summer	74.43636364	23.57575758	241	North River	Coast
7/6/2013	Summer	74	23.33333333	124	North River	Coast
7/7/2013	Summer	75.90909091	24.39393939	114	North River	Coast
7/8/2013	Summer	75.70909091	24.28282828	120	North River	Coast

7/9/2013	Summer	74.93636364	23.85353535	160	North River	Coast
7/10/2013	Summer	76.47272727	24.70707071	104	North River	Coast
7/11/2013	Summer	72.36363636	22.42424242	107	North River	Coast
7/12/2013	Summer	71.4	21.88888889	88	North River	Coast
7/13/2013	Summer	76.1	24.5	82	North River	Coast
7/14/2013	Summer	72.27272727	22.37373737	97	North River	Coast
7/15/2013	Summer	74.83333333	23.7962963	197	North River	Coast
7/16/2013	Summer	72.45454545	22.47474747	214	North River	Coast
7/18/2013	Summer	76.44545455	24.69191919	127	North River	Coast
7/19/2013	Summer	77.72727273	25.4040404	231	North River	Coast
7/20/2013	Summer	76.91818182	24.95454545	298	North River	Coast
7/21/2013	Summer	77.2	25.11111111	210	North River	Coast
7/22/2013	Summer	77	25	224	North River	Coast
7/23/2013	Summer	75.51818182	24.17676768	101	North River	Coast
7/24/2013	Summer	72.16363636	22.31313131	118	North River	Coast
7/25/2013	Summer	67.8	19.88888889	280	North River	Coast
7/26/2013	Summer	65.29090909	18.49494949	198	North River	Coast
7/27/2013	Summer	70.58181818	21.43434343	208	North River	Coast
7/28/2013	Summer	73.5	23.05555556	221	North River	Coast
7/29/2013	Summer	69.01818182	20.56565657	112	North River	Coast
7/30/2013	Summer	67.95454545	19.97474747	163	North River	Coast
7/31/2013	Summer	73.44545455	23.02525253	109	North River	Coast
8/1/2013	Summer	70.9	21.61111111	114	North River	Coast
8/2/2013	Summer	75.32727273	24.07070707	55	North River	Coast
8/3/2013	Summer	70.32727273	21.29292929	39	North River	Coast
8/4/2013	Summer	61.26363636	16.25757576	71	North River	Coast
8/5/2013	Summer	64.8	18.22222222	89	North River	Coast
8/6/2013	Summer	70.92727273	21.62626263	124	North River	Coast
8/7/2013	Summer	72.89090909	22.71717172	47	North River	Coast
8/8/2013	Summer	76.36	24.64444444	39	North River	Coast
8/9/2013	Summer	78.2	25.66666667	46	North River	Coast
8/10/2013	Summer	74.36	23.53333333	35	North River	Coast
8/11/2013	Summer	72.98181818	22.76767677	51	North River	Coast

8/12/2013	Summer	75.79090909	24.32828283	49	North River	Coast
8/13/2013	Summer	74.27272727	23.48484848	74	North River	Coast
8/14/2013	Summer	59.90909091	15.50505051	158	North River	Coast
8/15/2013	Summer	66.59090909	19.21717172	72	North River	Coast
8/16/2013	Summer	68.84545455	20.46969697	181	North River	Coast
8/17/2013	Summer	72.95454545	22.75252525	100	North River	Coast
8/18/2013	Summer	69.91818182	21.06565657	175	North River	Coast
8/19/2013	Summer	70.11818182	21.17676768	53	North River	Coast
8/20/2013	Summer	70.70909091	21.50505051	17	North River	Coast
8/21/2013	Summer	73.88181818	23.26767677	45	North River	Coast
8/22/2013	Summer	73.35454545	22.97474747	31	North River	Coast
8/23/2013	Summer	69.15	20.63888889	64	North River	Coast
8/24/2013	Summer	62.16666667	16.75925926	262	North River	Coast
8/25/2013	Summer	56.08333333	13.37962963	220	North River	Coast
8/26/2013	Summer	61.76666667	16.53703704	172	North River	Coast
8/27/2013	Summer	72.74166667	22.63425926	32	North River	Coast
8/28/2013	Summer	74.1	23.38888889	2	North River	Coast
8/29/2013	Summer	66.65	19.25	94	North River	Coast
8/30/2013	Summer	67.59166667	19.77314815	31	North River	Coast
8/31/2013	Summer	74.49166667	23.60648148	52	North River	Coast
9/2/2013	Fall	74.96666667	23.87037037	19	North River	Coast
9/3/2013	Fall	67.90833333	19.94907407	7	North River	Coast
9/4/2013	Fall	67.375	19.65277778	15	North River	Coast
9/5/2013	Fall	69.18461538	20.65811966	18	North River	Coast
9/6/2013	Fall	58.30769231	14.61538462	116	North River	Coast
9/7/2013	Fall	60.28461538	15.71367521	106	North River	Coast
9/8/2013	Fall	66.89230769	19.38461538	45	North River	Coast
9/9/2013	Fall	68.23846154	20.13247863	62	North River	Coast
9/10/2013	Fall	69.54615385	20.85897436	56	North River	Coast
9/11/2013	Fall	73.08333333	22.82407407	53	North River	Coast
9/12/2013	Fall	74.17692308	23.43162393	17	North River	Coast
9/13/2013	Fall	65.14615385	18.41452991	19	North River	Coast
9/14/2013	Fall	54.39230769	12.44017094	34	North River	Coast

9/15/2013	Fall	61.71538462	16.50854701	52	North River	Coast
9/16/2013	Fall	64.26153846	17.92307692	34	North River	Coast
9/17/2013	Fall	54.65384615	12.58547009	43	North River	Coast
9/18/2013	Fall	52.63846154	11.46581197	11	North River	Coast
9/19/2013	Fall	59.59230769	15.32905983	51	North River	Coast
9/20/2013	Fall	56.84615385	13.8034188	74	North River	Coast
9/21/2013	Fall	69.93076923	21.07264957	24	North River	Coast
9/22/2013	Fall	61.18461538	16.21367521	13	North River	Coast
9/23/2013	Fall	47.91538462	8.841880342	24	North River	Coast
9/24/2013	Fall	51.08461538	10.6025641	31	North River	Coast
9/25/2013	Fall	56.88461538	13.82478632	34	North River	Coast
9/26/2013	Fall	58.96153846	14.97863248	79	North River	Coast
9/27/2013	Fall	57.46153846	14.14529915	39	North River	Coast
9/28/2013	Fall	59.13846154	15.07692308	23	North River	Coast
9/29/2013	Fall	55.51538462	13.06410256	40	North River	Coast
9/30/2013	Fall	52.61538462	11.45299145	23	North River	Coast
10/1/2013	Fall	60.16923077	15.64957265	54	North River	Coast
10/2/2013	Fall	60.79230769	15.9957265	13	North River	Coast
10/10/2013	Fall	64.62307692	18.12393162	25	North River	Coast
10/11/2013	Fall	62.72307692	17.06837607	10	North River	Coast
10/12/2013	Fall	66.62307692	19.23504274	48	North River	Coast
10/13/2013	Fall	66.80769231	19.33760684	160	North River	Coast
10/14/2013	Fall	62.56153846	16.97863248	28	North River	Coast
10/15/2013	Fall	65.83846154	18.7991453	122	North River	Coast
10/16/2013	Fall	60.22142857	15.67857143	40	North River	Coast
10/17/2013	Fall	67.41428571	19.67460317	41	North River	Coast
10/18/2013	Fall	60.37142857	15.76190476	28	North River	Coast
10/19/2013	Fall	63.19285714	17.32936508	40	North River	Coast
10/20/2013	Fall	43.13571429	6.186507937	5	North River	Coast
10/21/2013	Fall	51.95714286	11.08730159	31	North River	Coast
10/22/2013	Fall	59.07857143	15.04365079	16	North River	Coast
10/23/2013	Fall	44.17142857	6.761904762	25	North River	Coast
10/24/2013	Fall	40.9	4.944444444	6	North River	Coast

10/25/2013	Fall	33.25384615	0.696581197	2	North River	Coast
10/26/2013	Fall	42.57857143	5.876984127	16	North River	Coast
10/27/2013	Fall	42.46428571	5.813492063	44	North River	Coast
10/28/2013	Fall	49.57142857	9.761904762	44	North River	Coast
10/29/2013	Fall	49.32857143	9.626984127	24	North River	Coast
10/30/2013	Fall	57.15	13.97222222	88	North River	Coast
10/31/2013	Fall	69.55	20.86111111	116	North River	Coast
11/1/2013	Fall	67.15	19.52777778	33	North River	Coast
11/2/2013	Fall	53.57692308	11.98717949	46	North River	Coast
11/10/2013	Fall	46.12666667	7.848148148	17	North River	Coast
11/11/2013	Fall	42.02	5.566666667	1	North River	Coast
11/12/2013	Fall	35.08	1.711111111	3	North River	Coast
11/13/2013	Fall	27.00666667	-2.774074074	1	North River	Coast
11/14/2013	Fall	35.93333333	2.185185185	0	North River	Coast
11/15/2013	Fall	53.54666667	11.97037037	59	North River	Coast
11/16/2013	Fall	52.78666667	11.54814815	14	North River	Coast
11/17/2013	Fall	65	18.33333333	46	North River	Coast
11/18/2013	Fall	50.52666667	10.29259259	29	North River	Coast
11/19/2013	Fall	39.81333333	4.340740741	3	North River	Coast
11/20/2013	Fall	49.72	9.844444444	2	North River	Coast
11/21/2013	Fall	49.99333333	9.996296296	22	North River	Coast
11/22/2013	Fall	62.68	17.04444444	45	North River	Coast
11/23/2013	Fall	39.87333333	4.374074074	1	North River	Coast
11/24/2013	Fall	26.47333333	-3.07037037	11	North River	Coast
11/25/2013	Fall	31.58666667	-0.22962963	0	North River	Coast
11/26/2013	Fall	63.78	17.65555556	4	North River	Coast
11/28/2013	Fall	27.29333333	-2.614814815	1	North River	Coast
11/29/2013	Fall	34.24666667	1.248148148	0	North River	Coast
11/30/2013	Fall	40.13333333	4.518518519	1	North River	Coast
12/1/2013	Winter	35.81333333	2.118518519	1	North River	Coast
12/2/2013	Winter	35.64666667	2.025925926	0	North River	Coast
12/3/2013	Winter	48.56666667	9.203703704	1	North River	Coast
12/4/2013	Winter	55.26	12.92222222	49	North River	Coast

12/5/2013	Winter	65.05333333	18.36296296	17	North River	Coast
12/6/2013	Winter	61.46	16.36666667	10	North River	Coast
12/7/2013	Winter	41.17333333	5.096296296	0	North River	Coast
12/11/2013	Winter	33.5	0.833333333	0	North River	Coast
12/12/2013	Winter	27.20666667	-2.662962963	1	North River	Coast
12/14/2013	Winter	54.84666667	12.69259259	0	North River	Coast
12/15/2013	Winter	38.14666667	3.414814815	1	North River	Coast
12/16/2013	Winter	37.71333333	3.174074074	0	North River	Coast
12/17/2013	Winter	43.15333333	6.196296296	1	North River	Coast
12/19/2013	Winter	44.57333333	6.985185185	1	North River	Coast
12/20/2013	Winter	56.29333333	13.4962963	27	North River	Coast
12/21/2013	Winter	64.97333333	18.31851852	6	North River	Coast
12/22/2013	Winter	69.22	20.67777778	79	North River	Coast
12/23/2013	Winter	48.83333333	9.351851852	4	North River	Coast
12/24/2013	Winter	31.86666667	-0.074074074	1	North River	Coast
12/25/2013	Winter	29.17333333	-1.57037037	0	North River	Coast
12/26/2013	Winter	30.06	-1.077777778	0	North River	Coast
12/27/2013	Winter	29.92666667	-1.151851852	0	North River	Coast
12/28/2013	Winter	45.82	7.677777778	0	North River	Coast
12/29/2013	Winter	47.92	8.844444444	2	North River	Coast
12/30/2013	Winter	43.44666667	6.359259259	3	North River	Coast
12/31/2013	Winter	33.74666667	0.97037037	0	North River	Coast
1/1/2014	Winter	43.02666667	6.125925926	0	North River	Coast
1/2/2014	Winter	42.27333333	5.707407407	0	North River	Coast
1/3/2014	Winter	23.19333333	-4.892592593	0	North River	Coast
1/4/2014	Winter	36.27333333	2.374074074	0	North River	Coast
1/5/2014	Winter	61.72666667	16.51481481	0	North River	Coast
1/6/2014	Winter	26.81333333	-2.881481481	0	North River	Coast
1/7/2014	Winter	16.62	-8.544444444	0	North River	Coast
1/8/2014	Winter	24.26	-4.3	0	North River	Coast
1/9/2014	Winter	36.34	2.411111111	0	North River	Coast
1/10/2014	Winter	58.99333333	14.9962963	0	North River	Coast
1/11/2014	Winter	55.03333333	12.7962963	2	North River	Coast

1/12/2014	Winter	38.34	3.522222222	0	North River	Coast
1/13/2014	Winter	55.46	13.03333333	4	North River	Coast
1/14/2014	Winter	42.31333333	5.72962963	0	North River	Coast
1/15/2014	Winter	42.75333333	5.974074074	0	North River	Coast
1/16/2014	Winter	31.18	-0.455555556	0	North River	Coast
1/17/2014	Winter	45.64666667	7.581481481	0	North River	Coast
1/18/2014	Winter	29.59333333	-1.337037037	0	North River	Coast
1/19/2014	Winter	38.2	3.444444444	0	North River	Coast
1/20/2014	Winter	42.05333333	5.585185185	1	North River	Coast
1/21/2014	Winter	26.54	-3.033333333	0	North River	Coast
1/22/2014	Winter	11.32666667	-11.48518519	0	North River	Coast
1/23/2014	Winter	24.66	-4.077777778	0	North River	Coast
1/24/2014	Winter	17.55333333	-8.025925926	0	North River	Coast
1/25/2014	Winter	33.23333333	0.685185185	0	North River	Coast
1/26/2014	Winter	41.18	5.1	0	North River	Coast
1/27/2014	Winter	35.72666667	2.07037037	4	North River	Coast
1/28/2014	Winter	19.5	-6.944444444	0	North River	Coast
1/29/2014	Winter	14.14666667	-9.918518519	0	North River	Coast
1/30/2014	Winter	32.78	0.433333333	0	North River	Coast
1/31/2014	Winter	29.65	-1.305555556	0	North River	Coast
2/1/2014	Winter	42.12142857	5.623015873	0	North River	Coast
2/2/2014	Winter	47.77857143	8.765873016	0	North River	Coast
2/3/2014	Winter	37.89285714	3.273809524	0	North River	Coast
2/4/2014	Winter	40.55714286	4.753968254	0	North River	Coast
2/6/2014	Winter	31.66428571	-0.186507937	0	North River	Coast
2/7/2014	Winter	35.35	1.861111111	0	North River	Coast
2/9/2014	Winter	39.91428571	4.396825397	0	North River	Coast
2/10/2014	Winter	31.49285714	-0.281746032	0	North River	Coast
2/11/2014	Winter	28.32857143	-2.03968254	0	North River	Coast
2/12/2014	Winter	46.27142857	7.928571429	0	North River	Coast
2/13/2014	Winter	36.82857143	2.682539683	0	North River	Coast
2/15/2014	Winter	32.4	0.222222222	0	North River	Coast
2/16/2014	Winter	36.7	2.611111111	0	North River	Coast

2/17/2014	Winter	36.5	2.5	0	North River	Coast
2/18/2014	Winter	46.57857143	8.099206349	0	North River	Coast
2/22/2014	Winter	42	5.555555556	0	North River	Coast
2/23/2014	Winter	50.06428571	10.03571429	11	North River	Coast
2/25/2014	Winter	37.54285714	3.079365079	0	North River	Coast
2/26/2014	Winter	32.86428571	0.48015873	0	North River	Coast
2/27/2014	Winter	35.29285714	1.829365079	0	North River	Coast
2/28/2014	Winter	31.35	-0.361111111	0	North River	Coast
3/1/2014	Spring	38.63571429	3.686507937	0	North River	Coast
3/2/2014	Spring	52.01428571	11.11904762	25	North River	Coast
3/3/2014	Spring	24.82857143	-3.984126984	0	North River	Coast
3/4/2014	Spring	31.68571429	-0.174603175	0	North River	Coast
3/5/2014	Spring	36.35714286	2.420634921	0	North River	Coast
3/6/2014	Spring	42	5.555555556	0	North River	Coast
3/7/2014	Spring	38.27142857	3.484126984	0	North River	Coast
3/8/2014	Spring	50.17333333	10.0962963	1	North River	Coast
3/31/2014	Spring	41.71666667	5.398148148	5	North River	Coast
4/1/2014	Spring	50.75833333	10.4212963	2	North River	Coast
4/2/2014	Spring	51.79166667	10.99537037	26	North River	Coast
4/3/2014	Spring	56.51666667	13.62037037	21	North River	Coast
4/4/2014	Spring	67.40833333	19.6712963	55	North River	Coast
4/5/2014	Spring	49.48333333	9.712962963	39	North River	Coast
4/6/2014	Spring	51.06666667	10.59259259	5	North River	Coast
4/7/2014	Spring	67.94166667	19.96759259	64	North River	Coast
4/8/2014	Spring	56.675	13.70833333	119	North River	Coast
4/9/2014	Spring	50.76666667	10.42592593	9	North River	Coast
4/10/2014	Spring	59.59166667	15.3287037	68	North River	Coast
4/11/2014	Spring	63.44166667	17.46759259	208	North River	Coast
4/12/2014	Spring	60.86666667	16.03703704	345	North River	Coast
4/13/2014	Spring	64.89166667	18.27314815	248	North River	Coast
4/14/2014	Spring	68.53333333	20.2962963	101	North River	Coast
4/15/2014	Spring	43.41666667	6.342592593	7	North River	Coast
4/16/2014	Spring	43.11666667	6.175925926	1	North River	Coast

4/17/2014	Spring	45.35	7.416666667	0	North River	Coast
4/18/2014	Spring	47.35833333	8.532407407	48	North River	Coast
4/19/2014	Spring	52.14166667	11.18981481	99	North River	Coast
4/20/2014	Spring	48.20833333	9.00462963	0	North River	Coast
4/21/2014	Spring	47.6	8.666666667	80	North River	Coast
4/22/2014	Spring	62.64545455	17.02525253	218	North River	Coast
4/24/2014	Spring	51.34545455	10.74747475	61	North River	Coast
4/25/2014	Spring	59.13636364	15.07575758	68	North River	Coast
4/27/2014	Spring	54.19090909	12.32828283	161	North River	Coast
4/28/2014	Spring	54.80909091	12.67171717	32	North River	Coast
4/29/2014	Spring	66.40909091	19.11616162	41	North River	Coast
4/30/2014	Spring	70.15454545	21.1969697	24	North River	Coast
5/1/2014	Spring	67.59090909	19.77272727	86	North River	Coast
5/2/2014	Spring	58.25454545	14.58585859	62	North River	Coast
5/3/2014	Spring	60.34545455	15.74747475	35	North River	Coast
5/4/2014	Spring	62.1	16.72222222	173	North River	Coast
5/5/2014	Spring	56.96363636	13.86868687	55	North River	Coast
5/6/2014	Spring	58.73636364	14.85353535	66	North River	Coast
5/7/2014	Spring	68.88181818	20.48989899	152	North River	Coast
5/8/2014	Spring	69.55454545	20.86363636	66	North River	Coast
5/9/2014	Spring	72.83636364	22.68686869	94	North River	Coast
5/10/2014	Spring	69.56363636	20.86868687	61	North River	Coast
5/11/2014	Spring	67.23636364	19.57575758	15	North River	Coast
5/12/2014	Spring	71.46363636	21.92424242	15	North River	Coast
5/13/2014	Spring	73.11818182	22.84343434	2	North River	Coast
5/14/2014	Spring	73.06363636	22.81313131	15	North River	Coast
5/15/2014	Spring	75.22727273	24.01515152	99	North River	Coast
5/16/2014	Spring	59.07272727	15.04040404	8	North River	Coast
5/17/2014	Spring	54.03636364	12.24242424	35	North River	Coast
5/18/2014	Spring	54.15454545	12.30808081	31	North River	Coast
5/19/2014	Spring	54.38181818	12.43434343	8	North River	Coast
5/20/2014	Spring	64.78181818	18.21212121	184	North River	Coast
5/21/2014	Spring	71.19	21.77222222	30	North River	Coast

5/22/2014	Spring	68.09	20.05	17	North River	Coast
5/23/2014	Spring	63.7	17.61111111	88	North River	Coast
5/24/2014	Spring	58.61818182	14.78787879	20	North River	Coast
5/25/2014	Spring	65.2	18.44444444	59	North River	Coast
5/26/2014	Spring	71.91818182	22.17676768	345	North River	Coast
5/27/2014	Spring	73.51818182	23.06565657	28	North River	Coast
5/28/2014	Spring	69.99090909	21.10606061	33	North River	Coast
5/29/2014	Spring	64.40909091	18.00505051	32	North River	Coast
5/30/2014	Spring	58.18181818	14.54545455	16	North River	Coast
5/31/2014	Spring	60.95454545	16.08585859	46	North River	Coast
6/1/2014	Summer	53.95454545	12.1969697	12	North River	Coast
6/2/2014	Summer	61.63636364	16.46464646	30	North River	Coast
6/3/2014	Summer	71.17272727	21.76262626	12	North River	Coast
6/4/2014	Summer	76.43636364	24.68686869	13	North River	Coast
6/6/2014	Summer	65.06363636	18.36868687	19	North River	Coast
6/7/2014	Summer	61.12727273	16.18181818	9	North River	Coast
6/8/2014	Summer	69.76363636	20.97979798	40	North River	Coast
6/9/2014	Summer	74.19090909	23.43939394	105	North River	Coast
6/10/2014	Summer	78.01818182	25.56565657	1	North River	Coast
6/11/2014	Summer	75.30909091	24.06060606	4	North River	Coast
6/12/2014	Summer	69.87272727	21.04040404	7	North River	Coast
6/13/2014	Summer	70.23636364	21.24242424	11	North River	Coast
6/14/2014	Summer	68.48181818	20.26767677	10	North River	Coast
6/15/2014	Summer	67.61818182	19.78787879	37	North River	Coast
6/16/2014	Summer	75.34545455	24.08080808	7	North River	Coast
6/17/2014	Summer	76.43636364	24.68686869	15	North River	Coast
6/18/2014	Summer	78.9	26.05555556	12	North River	Coast
6/19/2014	Summer	70.85	21.58333333	1	North River	Coast
6/20/2014	Summer	73.62	23.12222222	1	North River	Coast
6/21/2014	Summer	74.54	23.63333333	1	North River	Coast
6/22/2014	Summer	70.15	21.19444444	54	North River	Coast
6/23/2014	Summer	70.44	21.35555556	11	North River	Coast
6/24/2014	Summer	75.98	24.43333333	3	North River	Coast

6/25/2014	Summer	77.02	25.01111111	15	North River	Coast
6/26/2014	Summer	74.68	23.71111111	6	North River	Coast
6/27/2014	Summer	74.65	23.69444444	13	North River	Coast
6/28/2014	Summer	66.62	19.23333333	40	North River	Coast
6/29/2014	Summer	66.37	19.09444444	14	North River	Coast
6/30/2014	Summer	73	22.77777778	13	North River	Coast
7/1/2014	Summer	77.73	25.40555556	47	North River	Coast
7/2/2014	Summer	79.34	26.3	32	North River	Coast
7/3/2014	Summer	76.6	24.77777778	27	North River	Coast
7/4/2014	Summer	71.32	21.84444444	19	North River	Coast
7/5/2014	Summer	62.82	17.12222222	11	North River	Coast
7/6/2014	Summer	70.5	21.38888889	31	North River	Coast
7/7/2014	Summer	76.69	24.82777778	14	North River	Coast
7/8/2014	Summer	79.25	26.25	38	North River	Coast
7/9/2014	Summer	73	22.77777778	7	North River	Coast
7/10/2014	Summer	72.21	22.33888889	2	North River	Coast
7/11/2014	Summer	70.93	21.62777778	1	North River	Coast
7/12/2014	Summer	69.3	20.72222222	6	North River	Coast
7/13/2014	Summer	76.8	24.88888889	23	North River	Coast
7/14/2014	Summer	79.30909091	26.28282828	14	North River	Coast
7/15/2014	Summer	74.67272727	23.70707071	82	North River	Coast
7/16/2014	Summer	68.80909091	20.44949495	0	North River	Coast
7/17/2014	Summer	69.65454545	20.91919192	21	North River	Coast
7/18/2014	Summer	71.84545455	22.13636364	107	North River	Coast
7/28/2014	Summer	70.38181818	21.32323232	129	North River	Coast
7/29/2014	Summer	66.65454545	19.25252525	51	North River	Coast
7/31/2014	Summer	73.95454545	23.30808081	126	North River	Coast
8/1/2014	Summer	74.33636364	23.52020202	80	North River	Coast
8/2/2014	Summer	73.23636364	22.90909091	92	North River	Coast
8/3/2014	Summer	70.21818182	21.23232323	100	North River	Coast
8/4/2014	Summer	73.49090909	23.05050505	31	North River	Coast
8/5/2014	Summer	68.94545455	20.52525253	90	North River	Coast
8/6/2014	Summer	72.81818182	22.67676768	47	North River	Coast

8/7/2014	Summer	68.98181818	20.54545455	18	North River	Coast
8/8/2014	Summer	68.90909091	20.50505051	4	North River	Coast
8/9/2014	Summer	71.92727273	22.18181818	13	North River	Coast
8/10/2014	Summer	70.31818182	21.28787879	21	North River	Coast
8/11/2014	Summer	75.60909091	24.22727273	48	North River	Coast
8/12/2014	Summer	74.51818182	23.62121212	10	North River	Coast
8/13/2014	Summer	69.11818182	20.62121212	30	North River	Coast
8/14/2014	Summer	66.6	19.22222222	15	North River	Coast
8/15/2014	Summer	72.19090909	22.32828283	41	North River	Coast
8/16/2014	Summer	72.99166667	22.77314815	17	North River	Coast
8/17/2014	Summer	72.68333333	22.60185185	19	North River	Coast
8/18/2014	Summer	73.04166667	22.80092593	7	North River	Coast
8/19/2014	Summer	74.675	23.70833333	8	North River	Coast
8/20/2014	Summer	74.475	23.59722222	12	North River	Coast
8/21/2014	Summer	76.31666667	24.62037037	5	North River	Coast
8/22/2014	Summer	75.19166667	23.99537037	18	North River	Coast
8/23/2014	Summer	73.44166667	23.02314815	47	North River	Coast
8/24/2014	Summer	67.90833333	19.94907407	119	North River	Coast
8/25/2014	Summer	68.40833333	20.22685185	70	North River	Coast
8/26/2014	Summer	70.21666667	21.23148148	70	North River	Coast
8/27/2014	Summer	68.00833333	20.00462963	23	North River	Coast
8/28/2014	Summer	75.19166667	23.99537037	48	North River	Coast
8/29/2014	Summer	73.91666667	23.28703704	33	North River	Coast
8/30/2014	Summer	74.99166667	23.88425926	22	North River	Coast
8/31/2014	Summer	77.88333333	25.49074074	38	North River	Coast
9/22/2012	fall	69.07692308	20.5982906	3	parker	Coast
9/23/2012	fall	55.1	12.83333333	4	parker	Coast
9/24/2012	fall	50.28461538	10.15811966	0	parker	Coast
9/25/2012	fall	57.91538462	14.3974359	3	parker	Coast
9/26/2012	fall	62.33846154	16.85470085	7	parker	Coast
9/27/2012	fall	63.34615385	17.41452991	1	parker	Coast
9/28/2012	fall	68.98461538	20.54700855	1	parker	Coast
9/29/2012	fall	60.86153846	16.03418803	1	parker	Coast

9/30/2012	fall	63.89230769	17.71794872	1	parker	Coast
10/1/2012	fall	70.80769231	21.55982906	1	parker	Coast
10/2/2012	fall	74.13076923	23.40598291	0	parker	Coast
10/3/2012	fall	72.98461538	22.76923077	1	parker	Coast
10/4/2012	fall	66.61538462	19.23076923	3	parker	Coast
10/5/2012	fall	62.90769231	17.17094017	3	parker	Coast
10/6/2012	fall	66.60769231	19.22649573	2	parker	Coast
10/7/2012	fall	56.02307692	13.34615385	8	parker	Coast
10/8/2012	fall	57.37692308	14.0982906	21	parker	Coast
10/9/2012	fall	57.87692308	14.37606838	31	parker	Coast
10/10/2012	fall	54.93846154	12.74358974	2	parker	Coast
10/11/2012	fall	45.24615385	7.358974359	5	parker	Coast
10/12/2012	fall	52.99230769	11.66239316	31	parker	Coast
10/13/2012	fall	47.92307692	8.846153846	17	parker	Coast
10/14/2012	fall	60.53846154	15.85470085	4	parker	Coast
10/15/2012	fall	60.85384615	16.02991453	4	parker	Coast
10/16/2012	fall	47.02307692	8.346153846	4	parker	Coast
10/17/2012	fall	51.33571429	10.74206349	29	parker	Coast
10/18/2012	fall	66.82857143	19.34920635	0	parker	Coast
10/19/2012	fall	57.82857143	14.34920635	0	parker	Coast
10/20/2012	fall	52.32142857	11.28968254	3	parker	Coast
10/21/2012	fall	44.37142857	6.873015873	28	parker	Coast
10/22/2012	fall	46.53571429	8.075396825	56	parker	Coast
10/23/2012	fall	56.75	13.75	41	parker	Coast
10/24/2012	fall	57.59285714	14.21825397	0	parker	Coast
10/25/2012	fall	65.14285714	18.41269841	4	parker	Coast
10/26/2012	fall	66.47857143	19.1547619	5	parker	Coast
12/4/2012	Winter	59.02	15.01111111	11	parker	Coast
12/5/2012	Winter	47.27333333	8.485185185	15	parker	Coast
12/6/2012	Winter	42.5	5.833333333	0	parker	Coast
12/7/2012	Winter	61.65333333	16.47407407	23	parker	Coast
12/8/2012	Winter	53.84666667	12.13703704	0	parker	Coast
12/9/2012	Winter	61.90666667	16.61481481	8	parker	Coast

12/10/2012	Winter	62.48666667	16.93703704	8	parker	Coast
12/11/2012	Winter	49.69333333	9.82962963	5	parker	Coast
12/12/2012	Winter	43.90666667	6.614814815	0	parker	Coast
12/13/2012	Winter	41.54	5.3	0	parker	Coast
12/14/2012	Winter	35.88	2.155555556	0	parker	Coast
12/15/2012	Winter	49.57333333	9.762962963	0	parker	Coast
12/16/2012	Winter	57.64	14.24444444	3	parker	Coast
12/17/2012	Winter	57.84666667	14.35925926	5	parker	Coast
12/18/2012	Winter	45.62666667	7.57037037	0	parker	Coast
12/19/2012	Winter	43.88	6.6	0	parker	Coast
12/20/2012	Winter	58.55333333	14.75185185	5	parker	Coast
12/21/2012	Winter	39.44666667	4.137037037	2	parker	Coast
12/22/2012	Winter	33.06666667	0.592592593	0	parker	Coast
12/23/2012	Winter	42.57333333	5.874074074	0	parker	Coast
12/24/2012	Winter	49.11333333	9.507407407	2	parker	Coast
12/25/2012	Winter	47.82	8.788888889	17	parker	Coast
12/26/2012	Winter	51.45333333	10.80740741	2	parker	Coast
12/27/2012	Winter	37.43333333	3.018518519	0	parker	Coast
12/28/2012	Winter	41.75333333	5.418518519	0	parker	Coast
12/29/2012	Winter	41.77333333	5.42962963	0	parker	Coast
12/30/2012	Winter	31.90666667	-0.051851852	1	parker	Coast
12/31/2012	Winter	46.38666667	7.992592593	0	parker	Coast
1/1/2013	Winter	46.39333333	7.996296296	0	parker	Coast
1/2/2013	Winter	39.77333333	4.318518519	0	parker	Coast
1/3/2013	Winter	32.22	0.122222222	0	parker	Coast
1/4/2013	Winter	36.4	2.444444444	0	parker	Coast
1/5/2013	Winter	39.80666667	4.337037037	0	parker	Coast
1/6/2013	Winter	41.02666667	5.014814815	2	parker	Coast
1/7/2013	Winter	33.63333333	0.907407407	6	parker	Coast
1/8/2013	Winter	42.78	5.988888889	23	parker	Coast
1/9/2013	Winter	52.47333333	11.37407407	27	parker	Coast
1/10/2013	Winter	43.60666667	6.448148148	9	parker	Coast
1/11/2013	Winter	60.16666667	15.64814815	27	parker	Coast

1/12/2013	Winter	57.06666667	13.92592593	8	parker	Coast
1/13/2013	Winter	58.75333333	14.86296296	31	parker	Coast
1/14/2013	Winter	57.98666667	14.43703704	27	parker	Coast
1/15/2013	Winter	49.93333333	9.962962963	0	parker	Coast
1/16/2013	Winter	51.94	11.07777778	1	parker	Coast
1/17/2013	Winter	39.02666667	3.903703704	0	parker	Coast
1/18/2013	Winter	30.74	-0.7	0	parker	Coast
1/19/2013	Winter	44.51333333	6.951851852	0	parker	Coast
1/20/2013	Winter	40.27333333	4.596296296	15	parker	Coast
1/21/2013	Winter	44.64666667	7.025925926	10	parker	Coast
1/22/2013	Winter	25.86666667	-3.407407407	1	parker	Coast
1/23/2013	Winter	31.58	-0.233333333	0	parker	Coast
1/24/2013	Winter	24.98666667	-3.896296296	0	parker	Coast
1/25/2013	Winter	29.71333333	-1.27037037	0	parker	Coast
1/26/2013	Winter	29.62666667	-1.318518519	0	parker	Coast
1/27/2013	Winter	30.74666667	-0.696296296	0	parker	Coast
1/28/2013	Winter	50.74666667	10.41481481	28	parker	Coast
1/29/2013	Winter	58.63571429	14.79761905	8	parker	Coast
1/30/2013	Winter	64.34285714	17.96825397	5	parker	Coast
1/31/2013	Winter	39.74285714	4.301587302	3	parker	Coast
2/1/2013	Winter	25.76428571	-3.464285714	0	parker	Coast
2/2/2013	Winter	36.89285714	2.718253968	0	parker	Coast
2/3/2013	Winter	36.27142857	2.373015873	7	parker	Coast
2/4/2013	Winter	40.12857143	4.515873016	2	parker	Coast
2/5/2013	Winter	39.35714286	4.087301587	28	parker	Coast
2/6/2013	Winter	41.68571429	5.380952381	13	parker	Coast
2/7/2013	Winter	48.85714286	9.365079365	6	parker	Coast
2/8/2013	Winter	42.49285714	5.829365079	2	parker	Coast
2/9/2013	Winter	31.55	-0.25	2	parker	Coast
2/10/2013	Winter	47.46428571	8.591269841	133	parker	Coast
2/11/2013	Winter	60.42142857	15.78968254	29	parker	Coast
2/12/2013	Winter	49.42857143	9.682539683	11	parker	Coast
2/13/2013	Winter	46.78571429	8.214285714	2	parker	Coast

2/14/2013	Winter	36.49285714	2.496031746	3	parker	Coast
2/15/2013	Winter	40.67857143	4.821428571	4	parker	Coast
2/16/2013	Winter	32.32857143	0.182539683	2	parker	Coast
2/17/2013	Winter	25.62142857	-3.543650794	0	parker	Coast
2/18/2013	Winter	34.46428571	1.369047619	0	parker	Coast
2/19/2013	Winter	47.65714286	8.698412698	22	parker	Coast
2/20/2013	Winter	36.02142857	2.234126984	4	parker	Coast
2/21/2013	Winter	38.79285714	3.773809524	0	parker	Coast
2/22/2013	Winter	44.75	7.083333333	3	parker	Coast
2/23/2013	Winter	45.00714286	7.226190476	3	parker	Coast
2/24/2013	Winter	40.21428571	4.563492063	8	parker	Coast
2/25/2013	Winter	39.20714286	4.003968254	0	parker	Coast
2/26/2013	Winter	58.05	14.47222222	4	parker	Coast
2/27/2013	Winter	45.28571429	7.380952381	0	parker	Coast
2/28/2013	Winter	45.28571429	7.380952381	1	parker	Coast
3/1/2013	Spring	40.15	4.527777778	0	parker	Coast
3/2/2013	Spring	41.82857143	5.46031746	0	parker	Coast
3/3/2013	Spring	34.92142857	1.623015873	0	parker	Coast
3/4/2013	Spring	33.18571429	0.658730159	0	parker	Coast
3/5/2013	Spring	32.46428571	0.257936508	11	parker	Coast
3/12/2013	Spring	53.10769231	11.72649573	4	parker	Coast
3/13/2013	Spring	40.46153846	4.700854701	0	parker	Coast
3/14/2013	Spring	35.5	1.944444444	0	parker	Coast
3/15/2013	Spring	50.55384615	10.30769231	7	parker	Coast
3/16/2013	Spring	56.60769231	13.67094017	9	parker	Coast
3/17/2013	Spring	42.96153846	6.08974359	0	parker	Coast
3/18/2013	Spring	50.01538462	10.00854701	5	parker	Coast
3/19/2013	Spring	49.27692308	9.598290598	6	parker	Coast
3/20/2013	Spring	36.25384615	2.363247863	1	parker	Coast
3/21/2013	Spring	31.17692308	-0.457264957	0	parker	Coast
3/22/2013	Spring	33.26923077	0.705128205	0	parker	Coast
3/23/2013	Spring	37.07692308	2.820512821	4	parker	Coast
3/24/2013	Spring	39.73846154	4.299145299	0	parker	Coast

3/25/2013	Spring	38.95384615	3.863247863	0	parker	Coast
3/26/2013	Spring	38.96923077	3.871794872	0	parker	Coast
3/27/2013	Spring	39.81538462	4.341880342	0	parker	Coast
3/28/2013	Spring	35.75384615	2.085470085	0	parker	Coast
3/29/2013	Spring	41.05384615	5.02991453	4	parker	Coast
3/30/2013	Spring	48.64615385	9.247863248	4	parker	Coast
3/31/2013	Spring	58.54615385	14.74786325	3	parker	Coast
4/1/2013	Spring	51.28461538	10.71367521	7	parker	Coast
4/2/2013	Spring	42.24615385	5.692307692	2	parker	Coast
4/3/2013	Spring	42.94166667	6.078703704	8	parker	Coast
4/4/2013	Spring	47.5	8.611111111	0	parker	Coast
4/5/2013	Spring	41.75833333	5.421296296	2	parker	Coast
4/6/2013	Spring	37.50833333	3.060185185	0	parker	Coast
4/7/2013	Spring	54.23333333	12.35185185	15	parker	Coast
4/8/2013	Spring	61.23333333	16.24074074	15	parker	Coast
4/9/2013	Spring	64.125	17.84722222	8	parker	Coast
4/10/2013	Spring	65.35	18.52777778	4	parker	Coast
4/11/2013	Spring	69.59166667	20.88425926	9	parker	Coast
4/12/2013	Spring	64.21666667	17.89814815	11	parker	Coast
4/13/2013	Spring	50.09166667	10.05092593	5	parker	Coast
4/14/2013	Spring	59.86666667	15.48148148	6	parker	Coast
4/15/2013	Spring	61.75833333	16.53240741	1	parker	Coast
4/16/2013	Spring	57.375	14.09722222	6	parker	Coast
4/17/2013	Spring	61.95833333	16.64351852	21	parker	Coast
4/18/2013	Spring	65.29090909	18.49494949	9	parker	Coast
4/19/2013	Spring	65.6	18.66666667	2	parker	Coast
4/20/2013	Spring	44.97272727	7.207070707	0	parker	Coast
4/21/2013	Spring	44.79090909	7.106060606	0	parker	Coast
4/22/2013	Spring	50.44545455	10.24747475	0	parker	Coast
4/23/2013	Spring	45.17272727	7.318181818	0	parker	Coast
4/24/2013	Spring	62.70909091	17.06060606	16	parker	Coast
4/25/2013	Spring	42.50909091	5.838383838	0	parker	Coast
4/26/2013	Spring	48.5	9.166666667	1	parker	Coast

4/27/2013	Spring	48.48181818	9.156565657	3	parker	Coast
4/28/2013	Spring	60.99090909	16.10606061	2	parker	Coast
4/29/2013	Spring	58.96363636	14.97979798	2	parker	Coast
4/30/2013	Spring	60.56363636	15.86868687	9	parker	Coast
5/1/2013	Spring	58.68181818	14.82323232	30	parker	Coast
5/2/2013	Spring	54.2	12.33333333	14	parker	Coast
5/3/2013	Spring	53.22727273	11.79292929	4	parker	Coast
5/4/2013	Spring	52.25454545	11.25252525	3	parker	Coast
5/5/2013	Spring	59.56363636	15.31313131	7	parker	Coast
6/21/2013	Summer	65.80909091	18.78282828	8	parker	Coast
6/22/2013	Summer	72.42727273	22.45959596	5	parker	Coast
6/23/2013	Summer	73.60909091	23.11616162	9	parker	Coast
6/24/2013	Summer	72.21818182	22.34343434	6	parker	Coast
6/25/2013	Summer	74.32727273	23.51515152	7	parker	Coast
6/26/2013	Summer	73.24545455	22.91414141	8	parker	Coast
6/27/2013	Summer	75.38181818	24.1010101	14	parker	Coast
6/28/2013	Summer	73.09090909	22.82828283	17	parker	Coast
6/29/2013	Summer	75.56363636	24.2020202	8	parker	Coast
6/30/2013	Summer	77	25	6	parker	Coast
7/1/2013	Summer	74.43636364	23.57575758	6	parker	Coast
7/2/2013	Summer	75.3	24.05555556	7	parker	Coast
7/3/2013	Summer	75.02727273	23.9040404	3	parker	Coast
7/4/2013	Summer	74.76363636	23.75757576	8	parker	Coast
7/5/2013	Summer	74.10909091	23.39393939	6	parker	Coast
7/6/2013	Summer	74.26363636	23.47979798	9	parker	Coast
7/7/2013	Summer	75.94545455	24.41414141	12	parker	Coast
7/8/2013	Summer	75.74545455	24.3030303	9	parker	Coast
7/9/2013	Summer	74.66363636	23.7020202	17	parker	Coast
7/10/2013	Summer	75.98181818	24.43434343	34	parker	Coast
7/11/2013	Summer	73.65454545	23.14141414	16	parker	Coast
7/12/2013	Summer	72.88181818	22.71212121	28	parker	Coast
7/13/2013	Summer	75.91818182	24.3989899	20	parker	Coast
7/14/2013	Summer	74.39090909	23.55050505	17	parker	Coast

7/15/2013	Summer	75.85454545	24.36363636	8	parker	Coast
7/16/2013	Summer	75.6	24.22222222	11	parker	Coast
7/17/2013	Summer	75.31818182	24.06565657	16	parker	Coast
7/18/2013	Summer	77.35454545	25.1969697	15	parker	Coast
7/19/2013	Summer	78.02727273	25.57070707	6	parker	Coast
7/20/2013	Summer	76.85454545	24.91919192	14	parker	Coast
7/21/2013	Summer	77.06363636	25.03535354	4	parker	Coast
7/22/2013	Summer	77.12727273	25.07070707	5	parker	Coast
7/23/2013	Summer	76.92727273	24.95959596	11	parker	Coast
7/24/2013	Summer	74.71818182	23.73232323	12	parker	Coast
7/25/2013	Summer	71.27272727	21.81818182	18	parker	Coast
7/26/2013	Summer	68.03636364	20.02020202	16	parker	Coast
7/27/2013	Summer	72.46363636	22.47979798	13	parker	Coast
7/28/2013	Summer	75.32727273	24.07070707	11	parker	Coast
7/29/2013	Summer	72.81818182	22.67676768	6	parker	Coast
7/30/2013	Summer	70.19090909	21.21717172	11	parker	Coast
7/31/2013	Summer	73.83636364	23.24242424	20	parker	Coast
8/1/2013	Summer	75.04545455	23.91414141	12	parker	Coast
8/2/2013	Summer	76.61818182	24.78787879	15	parker	Coast
8/3/2013	Summer	72.66363636	22.59090909	10	parker	Coast
8/4/2013	Summer	64.01818182	17.78787879	8	parker	Coast
8/5/2013	Summer	67.43636364	19.68686869	6	parker	Coast
8/6/2013	Summer	68.98181818	20.54545455	11	parker	Coast
8/7/2013	Summer	73.05454545	22.80808081	10	parker	Coast
8/8/2013	Summer	76.67272727	24.81818182	16	parker	Coast
8/9/2013	Summer	78.76363636	25.97979798	6	parker	Coast
8/10/2013	Summer	74.34545455	23.52525253	16	parker	Coast
8/11/2013	Summer	74.98181818	23.87878788	13	parker	Coast
8/12/2013	Summer	78.00909091	25.56060606	16	parker	Coast
8/13/2013	Summer	75.40909091	24.11616162	2	parker	Coast
8/14/2013	Summer	66.60909091	19.22727273	3	parker	Coast
8/15/2013	Summer	69.28181818	20.71212121	8	parker	Coast
8/16/2013	Summer	69.47272727	20.81818182	12	parker	Coast

8/17/2013	Summer	73.47272727	23.04040404	6	parker	Coast
8/18/2013	Summer	73.64545455	23.13636364	13	parker	Coast
8/19/2013	Summer	72.62727273	22.57070707	9	parker	Coast
8/20/2013	Summer	72.56666667	22.53703704	12	parker	Coast
8/21/2013	Summer	73.50833333	23.06018519	12	parker	Coast
8/22/2013	Summer	73.25833333	22.9212963	17	parker	Coast
8/23/2013	Summer	70.00833333	21.11574074	11	parker	Coast
8/24/2013	Summer	68.525	20.29166667	10	parker	Coast
8/25/2013	Summer	59.24166667	15.13425926	9	parker	Coast
8/26/2013	Summer	64.01666667	17.78703704	7	parker	Coast
8/27/2013	Summer	72.075	22.26388889	1	parker	Coast
8/28/2013	Summer	76.09166667	24.49537037	9	parker	Coast
8/29/2013	Summer	68.95833333	20.53240741	18	parker	Coast
8/30/2013	Summer	68.43333333	20.24074074	17	parker	Coast
8/31/2013	Summer	74.30833333	23.50462963	11	parker	Coast
9/1/2013	Fall	72.525	22.51388889	8	parker	Coast
9/2/2013	Fall	75.075	23.93055556	15	parker	Coast
9/3/2013	Fall	68.275	20.15277778	14	parker	Coast
10/9/2013	Fall	64.71538462	18.17521368	3	parker	Coast
10/10/2013	Fall	62.10769231	16.72649573	6	parker	Coast
10/11/2013	Fall	62.68461538	17.04700855	5	parker	Coast
10/12/2013	Fall	67.18461538	19.54700855	11	parker	Coast
10/13/2013	Fall	68.03846154	20.02136752	7	parker	Coast
10/14/2013	Fall	63.06153846	17.25641026	6	parker	Coast
10/15/2013	Fall	66.37692308	19.0982906	11	parker	Coast
10/16/2013	Fall	61.93076923	16.62820513	13	parker	Coast
10/17/2013	Fall	68.23076923	20.12820513	13	parker	Coast
10/18/2013	Fall	63.32142857	17.40079365	3	parker	Coast
10/19/2013	Fall	64.65	18.13888889	8	parker	Coast
10/20/2013	Fall	47.70714286	8.726190476	6	parker	Coast
10/21/2013	Fall	54.96428571	12.75793651	3	parker	Coast
10/22/2013	Fall	59.68571429	15.38095238	1	parker	Coast
10/23/2013	Fall	47.79285714	8.773809524	8	parker	Coast

10/24/2013	Fall	44.42142857	6.900793651	5	parker	Coast
10/25/2013	Fall	35.47692308	1.931623932	0	parker	Coast
10/26/2013	Fall	43.23571429	6.242063492	2	parker	Coast
10/27/2013	Fall	44.85714286	7.142857143	2	parker	Coast
10/28/2013	Fall	50.88571429	10.49206349	368	parker	Coast
10/29/2013	Fall	51.12857143	10.62698413	70	parker	Coast
10/30/2013	Fall	57.88571429	14.38095238	25	parker	Coast
10/31/2013	Fall	69.76428571	20.98015873	21	parker	Coast
11/1/2013	Fall	68.92666667	20.51481481	7	parker	Coast
11/2/2013	Fall	56.15714286	13.42063492	22	parker	Coast
11/14/2013	Fall	35.71333333	2.062962963	0	parker	Coast
11/15/2013	Fall	56.96666667	13.87037037	0	parker	Coast
11/16/2013	Fall	55.62	13.12222222	4	parker	Coast
11/17/2013	Fall	67.08666667	19.49259259	5	parker	Coast
11/18/2013	Fall	52.75333333	11.52962963	6	parker	Coast
11/19/2013	Fall	44.66666667	7.037037037	0	parker	Coast
11/20/2013	Fall	49.88	9.933333333	0	parker	Coast
11/21/2013	Fall	51.08	10.6	0	parker	Coast
11/22/2013	Fall	64.06666667	17.81481481	4	parker	Coast
11/23/2013	Fall	44.18666667	6.77037037	0	parker	Coast
11/24/2013	Fall	29.49333333	-1.392592593	0	parker	Coast
11/25/2013	Fall	33.17333333	0.651851852	0	parker	Coast
11/26/2013	Fall	65.05333333	18.36296296	0	parker	Coast
12/19/2013	Winter	45.2	7.333333333	0	parker	Coast
12/20/2013	Winter	58.26	14.58888889	4	parker	Coast
12/23/2013	Winter	53.34666667	11.85925926	0	parker	Coast
12/24/2013	Winter	34.98	1.655555556	1	parker	Coast
12/25/2013	Winter	34.22	1.233333333	1	parker	Coast
12/26/2013	Winter	33.10666667	0.614814815	0	parker	Coast
12/27/2013	Winter	31.76	-0.133333333	0	parker	Coast
12/28/2013	Winter	47.55333333	8.640740741	7	parker	Coast
12/29/2013	Winter	52.18	11.21111111	8	parker	Coast
12/30/2013	Winter	45.5	7.5	4	parker	Coast

12/31/2013	Winter	36.04	2.244444444	0	parker	Coast
1/1/2014	Winter	45.37333333	7.42962963	0	parker	Coast
1/2/2014	Winter	45.52	7.511111111	0	parker	Coast
1/3/2014	Winter	25.98666667	-3.340740741	0	parker	Coast
1/4/2014	Winter	38.64	3.688888889	0	parker	Coast
1/5/2014	Winter	62.96	17.2	22	parker	Coast
1/6/2014	Winter	28.71875	-1.822916667	0	parker	Coast
1/7/2014	Winter	17.88	-7.844444444	0	parker	Coast
1/8/2014	Winter	26.26	-3.188888889	0	parker	Coast
1/9/2014	Winter	41.58666667	5.325925926	0	parker	Coast
1/10/2014	Winter	60.72	15.95555556	4	parker	Coast
1/11/2014	Winter	56.44666667	13.58148148	2	parker	Coast
1/12/2014	Winter	40.74	4.855555556	3	parker	Coast
1/13/2014	Winter	55.35333333	12.97407407	5	parker	Coast
1/14/2014	Winter	43.60666667	6.448148148	0	parker	Coast
1/15/2014	Winter	43.86666667	6.592592593	0	parker	Coast
1/16/2014	Winter	30.99333333	-0.559259259	1	parker	Coast
1/17/2014	Winter	46.22	7.9	0	parker	Coast
1/18/2014	Winter	29.76	-1.244444444	0	parker	Coast
1/19/2014	Winter	39.45333333	4.140740741	0	parker	Coast
1/20/2014	Winter	35.46	1.922222222	0	parker	Coast
2/26/2014	Winter	32.27857143	0.154761905	0	parker	Coast
2/27/2014	Winter	33.20714286	0.670634921	0	parker	Coast
2/28/2014	Winter	32.45714286	0.253968254	0	parker	Coast
3/1/2014	Spring	33.04285714	0.579365079	0	parker	Coast
3/2/2014	Spring	51.55	10.86111111	0	parker	Coast
3/3/2014	Spring	26.1	-3.277777778	0	parker	Coast
3/4/2014	Spring	31.13571429	-0.48015873	0	parker	Coast
3/5/2014	Spring	36.58571429	2.547619048	0	parker	Coast
3/6/2014	Spring	42.06428571	5.591269841	1	parker	Coast
3/7/2014	Spring	37.98461538	3.324786325	0	parker	Coast
3/8/2014	Spring	47.55	8.638888889	0	parker	Coast
3/30/2014	Spring	45.24615385	7.358974359	1	parker	Coast

6/6/2014	Summer	64.88181818	18.26767677	4	parker	Coast
6/7/2014	Summer	60.95454545	16.08585859	4	parker	Coast
6/8/2014	Summer	68.12727273	20.07070707	8	parker	Coast
6/9/2014	Summer	74.93636364	23.85353535	8	parker	Coast
6/10/2014	Summer	76.83636364	24.90909091	12	parker	Coast
6/11/2014	Summer	72.8	22.66666667	11	parker	Coast
6/12/2014	Summer	70.68181818	21.48989899	7	parker	Coast
6/13/2014	Summer	68.88181818	20.48989899	4	parker	Coast
6/14/2014	Summer	69.77272727	20.98484848	13	parker	Coast
6/15/2014	Summer	68.37272727	20.20707071	12	parker	Coast
6/16/2014	Summer	75.54545455	24.19191919	37	parker	Coast
6/17/2014	Summer	76.17272727	24.54040404	12	parker	Coast
6/18/2014	Summer	78.67272727	25.92929293	13	parker	Coast
6/19/2014	Summer	72.52727273	22.51515152	11	parker	Coast
6/20/2014	Summer	74.79090909	23.77272727	7	parker	Coast
6/21/2014	Summer	72.10909091	22.28282828	44	parker	Coast
6/22/2014	Summer	70.29090909	21.27272727	26	parker	Coast
6/23/2014	Summer	67.82727273	19.9040404	25	parker	Coast
6/24/2014	Summer	75.61818182	24.23232323	11	parker	Coast
6/25/2014	Summer	77.34545455	25.19191919	12	parker	Coast
6/26/2014	Summer	76.93636364	24.96464646	4	parker	Coast
6/27/2014	Summer	74.49090909	23.60606061	4	parker	Coast
6/28/2014	Summer	66.25454545	19.03030303	2	parker	Coast
6/29/2014	Summer	66.06363636	18.92424242	6	parker	Coast
6/30/2014	Summer	71.68181818	22.04545455	13	parker	Coast
7/1/2014	Summer	77.06363636	25.03535354	15	parker	Coast
7/2/2014	Summer	78.60909091	25.89393939	9	parker	Coast
7/3/2014	Summer	76.10909091	24.50505051	18	parker	Coast
7/4/2014	Summer	71.4	21.88888889	10	parker	Coast
7/5/2014	Summer	63.43636364	17.46464646	12	parker	Coast
7/6/2014	Summer	69.9	21.05555556	10	parker	Coast
7/7/2014	Summer	76.6	24.77777778	5	parker	Coast
7/28/2014	Summer	69.76363636	20.97979798	9	parker	Coast

7/29/2014	Summer	65.5	18.61111111	6	parker	Coast
7/30/2014	Summer	65.92727273	18.84848485	5	parker	Coast
7/31/2014	Summer	71.07272727	21.70707071	5	parker	Coast
8/1/2014	Summer	73.08181818	22.82323232	5	parker	Coast
8/2/2014	Summer	71.99090909	22.21717172	3	parker	Coast
8/3/2014	Summer	68.94545455	20.52525253	8	parker	Coast
8/4/2014	Summer	72.8	22.66666667	7	parker	Coast
8/5/2014	Summer	69.55454545	20.86363636	6	parker	Coast
8/6/2014	Summer	71.54545455	21.96969697	3	parker	Coast
8/7/2014	Summer	67.13636364	19.52020202	8	parker	Coast
8/8/2014	Summer	70.5	21.38888889	11	parker	Coast
8/9/2014	Summer	69.79090909	20.99494949	5	parker	Coast
8/10/2014	Summer	67.71818182	19.84343434	4	parker	Coast
8/11/2014	Summer	72.28181818	22.37878788	2	parker	Coast
8/12/2014	Summer	72.84545455	22.69191919	5	parker	Coast
8/13/2014	Summer	66.42727273	19.12626263	13	parker	Coast
8/14/2014	Summer	63.53636364	17.52020202	8	parker	Coast
8/15/2014	Summer	68.94545455	20.52525253	11	parker	Coast
8/16/2014	Summer	70.16363636	21.2020202	19	parker	Coast
8/17/2014	Summer	70.82727273	21.57070707	20	parker	Coast
8/18/2014	Summer	71.24545455	21.8030303	7	parker	Coast
8/19/2014	Summer	73.39090909	22.99494949	12	parker	Coast
8/20/2014	Summer	73.775	23.20833333	5	parker	Coast
8/21/2014	Summer	75.05833333	23.9212963	9	parker	Coast
8/22/2014	Summer	73.58333333	23.10185185	11	parker	Coast
8/23/2014	Summer	72.40833333	22.44907407	3	parker	Coast
8/24/2014	Summer	67.24166667	19.5787037	4	parker	Coast
8/25/2014	Summer	66.20833333	19.00462963	9	parker	Coast
8/26/2014	Summer	68.76666667	20.42592593	9	parker	Coast
8/27/2014	Summer	65.975	18.875	5	parker	Coast
8/28/2014	Summer	74.06666667	23.37037037	11	parker	Coast
8/29/2014	Summer	71.74166667	22.0787037	2	parker	Coast
8/30/2014	Summer	72.58333333	22.5462963	4	parker	Coast

8/31/2014	Summer	75.26666667	24.03703704	5	parker	Coast
9/22/2012	fall	69.10769231	20.61538462	533	South River	Coast
9/23/2012	fall	57.33846154	14.07692308	80	South River	Coast
9/24/2012	fall	53.92727273	12.18181818	67	South River	Coast
9/25/2012	fall	59.69230769	15.38461538	121	South River	Coast
9/26/2012	fall	62.46153846	16.92307692	123	South River	Coast
9/27/2012	fall	65.92307692	18.84615385	213	South River	Coast
9/28/2012	fall	69.66153846	20.92307692	136	South River	Coast
9/29/2012	fall	65.9	18.83333333	47	South River	Coast
9/30/2012	fall	63.01538462	17.23076923	51	South River	Coast
10/1/2012	fall	72.43076923	22.46153846	53	South River	Coast
10/2/2012	fall	75.33846154	24.07692308	282	South River	Coast
10/3/2012	fall	73.12307692	22.84615384	127	South River	Coast
10/4/2012	fall	67.72307692	19.84615384	72	South River	Coast
10/5/2012	fall	62.87692308	17.15384616	163	South River	Coast
10/6/2012	fall	68.83076923	20.46153846	169	South River	Coast
10/7/2012	fall	55.95384615	13.30769231	83	South River	Coast
10/8/2012	fall	50.75	10.41666667	4	South River	Coast
10/9/2012	fall	53.6	12	49	South River	Coast
10/10/2012	fall	55.26153846	12.92307692	59	South River	Coast
10/11/2012	fall	49.16923077	9.538461539	42	South River	Coast
10/12/2012	fall	53.6	12	36	South River	Coast
10/13/2012	fall	52.21538462	11.23076923	30	South River	Coast
10/14/2012	fall	61.76923077	16.53846154	270	South River	Coast
10/15/2012	fall	58.44615385	14.69230769	229	South River	Coast
10/16/2012	fall	49.86153846	9.923076922	28	South River	Coast
10/17/2012	fall	51.93846154	11.07692308	28	South River	Coast
10/19/2012	fall	54.29230769	12.38461538	132	South River	Coast
10/20/2012	fall	52.82857143	11.57142857	53	South River	Coast
10/21/2012	fall	47.3	8.5	27	South River	Coast
10/22/2012	fall	51.02857143	10.57142857	35	South River	Coast
10/23/2012	fall	56.23076923	13.46153846	84	South River	Coast
10/24/2012	fall	58.74285714	14.85714286	278	South River	Coast

10/25/2012	fall	59.25714286	15.14285714	133	South River	Coast
10/26/2012	fall	66.45714286	19.14285714	137	South River	Coast
10/27/2012	fall	62.85714286	17.14285714	25	South River	Coast
10/28/2012	fall	55.91428571	13.28571428	28	South River	Coast
10/29/2012	fall	43.18571429	6.214285714	9	South River	Coast
10/30/2012	fall	45.08	7.266666667	34	South River	Coast
10/31/2012	fall	41.48	5.266666667	13	South River	Coast
11/1/2012	fall	41.36	5.2	11	South River	Coast
11/3/2012	fall	45.75714286	7.642857144	9	South River	Coast
12/1/2012	Winter	45.75714286	7.642857144	0	South River	Coast
12/2/2012	Winter	54.5	12.5	0	South River	Coast
12/3/2012	Winter	54.5	12.5	0	South River	Coast
12/4/2012	Winter	58.04	14.46666667	418	South River	Coast
12/6/2012	Winter	46.32666667	7.959259261	1	South River	Coast
12/7/2012	Winter	58.46	14.7	239	South River	Coast
12/8/2012	Winter	54.86	12.7	104	South River	Coast
12/9/2012	Winter	63.87333333	17.70740741	426	South River	Coast
12/10/2012	Winter	64.66666667	18.14814815	264	South River	Coast
12/11/2012	Winter	54.44	12.46666667	30	South River	Coast
12/12/2012	Winter	40.69333333	4.829629628	0	South River	Coast
12/13/2012	Winter	39.8	4.333333333	0	South River	Coast
12/14/2012	Winter	37.25333333	2.918518517	0	South River	Coast
12/15/2012	Winter	51.70666667	10.94814815	8	South River	Coast
12/16/2012	Winter	57.92	14.4	24	South River	Coast
12/17/2012	Winter	57.44666667	14.13703704	3	South River	Coast
12/18/2012	Winter	46.24	7.911111111	112	South River	Coast
12/19/2012	Winter	44.82666667	7.125925928	57	South River	Coast
12/20/2012	Winter	57.28666667	14.04814815	141	South River	Coast
12/21/2012	Winter	38.37333333	3.540740739	0	South River	Coast
12/22/2012	Winter	35.38	1.877777778	1	South River	Coast
12/23/2012	Winter	46.5	8.055555556	1	South River	Coast
12/24/2012	Winter	49.29333333	9.607407406	20	South River	Coast
12/25/2012	Winter	51	10.55555556	34	South River	Coast

12/26/2012	Winter	47.51333333	8.618518517	0	South River	Coast
12/27/2012	Winter	38.51333333	3.618518517	1	South River	Coast
12/28/2012	Winter	43.12	6.177777778	2	South River	Coast
12/30/2012	Winter	31.06	-0.522222222	2	South River	Coast
12/31/2012	Winter	51.14666667	10.63703704	9	South River	Coast
1/1/2013	Winter	52.46	11.36666667	0	South River	Coast
1/2/2013	Winter	43.09333333	6.162962961	0	South River	Coast
1/4/2013	Winter	35.20714286	1.781746033	0	South River	Coast
1/5/2013	Winter	41.52	5.288888889	0	South River	Coast
1/6/2013	Winter	42.55333333	5.862962961	0	South River	Coast
1/7/2013	Winter	39.48666667	4.159259261	0	South River	Coast
1/8/2013	Winter	46.66	8.144444444	37	South River	Coast
1/9/2013	Winter	56.52857143	13.62698413	267	South River	Coast
1/10/2013	Winter	54.50666667	12.50370371	121	South River	Coast
1/11/2013	Winter	62.08666667	16.71481482	263	South River	Coast
2/19/2013	Winter	45.88571429	7.714285717	9	South River	Coast
2/20/2013	Winter	37.20714286	2.892857144	0	South River	Coast
2/21/2013	Winter	44.32857143	6.84920635	7	South River	Coast
2/22/2013	Winter	40.5	4.722222222	0	South River	Coast
2/23/2013	Winter	45.59285714	7.5515873	3	South River	Coast
2/24/2013	Winter	44.08571429	6.714285717	0	South River	Coast
2/25/2013	Winter	41.62142857	5.345238094	2	South River	Coast
2/26/2013	Winter	55.19285714	12.88492063	18	South River	Coast
2/27/2013	Winter	47.00714286	8.337301589	19	South River	Coast
2/28/2013	Winter	38.97857143	3.876984128	0	South River	Coast
3/1/2013	Spring	42.57857143	5.876984128	2	South River	Coast
3/2/2013	Spring	36.7	2.611111111	8	South River	Coast
3/3/2013	Spring	34.93571429	1.630952383	3	South River	Coast
3/4/2013	Spring	37.36428571	2.980158728	0	South River	Coast
3/5/2013	Spring	54.27142857	12.37301587	15	South River	Coast
3/6/2013	Spring	41.6	5.333333333	7	South River	Coast
3/7/2013	Spring	40.26153846	4.58974359	3	South River	Coast
3/8/2013	Spring	42.86428571	6.035714283	0	South River	Coast

3/9/2013	Spring	41.37142857	5.206349206	9	South River	Coast
3/12/2013	Spring	52.08571429	11.15873016	75	South River	Coast
3/13/2013	Spring	42.07142857	5.595238094	33	South River	Coast
3/14/2013	Spring	38.00714286	3.337301589	2	South River	Coast
3/15/2013	Spring	54.38461538	12.43589743	32	South River	Coast
3/16/2013	Spring	63.96923077	17.76068376	16	South River	Coast
3/17/2013	Spring	49.79230769	9.884615383	19	South River	Coast
3/18/2013	Spring	51.11538462	10.61965812	30	South River	Coast
3/19/2013	Spring	50.18333333	10.10185185	15	South River	Coast
3/20/2013	Spring	38.13333333	3.407407406	2	South River	Coast
3/21/2013	Spring	33.91666667	1.064814817	1	South River	Coast
3/22/2013	Spring	38.075	3.375	1	South River	Coast
3/23/2013	Spring	45.18333333	7.324074072	3	South River	Coast
3/24/2013	Spring	40.925	4.958333333	3	South River	Coast
3/25/2013	Spring	39.48333333	4.157407406	0	South River	Coast
3/26/2013	Spring	39.325	4.069444444	0	South River	Coast
3/27/2013	Spring	38.95	3.861111111	0	South River	Coast
3/28/2013	Spring	37.84166667	3.245370372	0	South River	Coast
3/29/2013	Spring	50.55833333	10.31018518	3	South River	Coast
3/30/2013	Spring	50.71666667	10.39814815	23	South River	Coast
3/31/2013	Spring	60.23333333	15.68518518	13	South River	Coast
4/1/2013	Spring	53.06923077	11.70512821	15	South River	Coast
4/2/2013	Spring	48.65833333	9.254629628	0	South River	Coast
4/3/2013	Spring	47.18333333	8.435185183	1	South River	Coast
4/4/2013	Spring	43.05	6.138888889	1	South River	Coast
4/5/2013	Spring	44.225	6.791666667	1	South River	Coast
4/6/2013	Spring	44.84166667	7.134259261	1	South River	Coast
4/7/2013	Spring	56.625	13.68055556	5	South River	Coast
4/8/2013	Spring	60.85	16.02777778	20	South River	Coast
4/9/2013	Spring	65.05	18.36111111	12	South River	Coast
4/10/2013	Spring	63.58333333	17.5462963	16	South River	Coast
4/11/2013	Spring	70.48333333	21.37962963	14	South River	Coast
4/12/2013	Spring	63.525	17.51388889	70	South River	Coast

4/13/2013	Spring	53.21666667	11.78703704	16	South River	Coast
4/14/2013	Spring	63.08333333	17.26851852	56	South River	Coast
4/15/2013	Spring	59.45	15.25	112	South River	Coast
4/16/2013	Spring	59.90833333	15.50462963	214	South River	Coast
4/17/2013	Spring	62.175	16.76388889	267	South River	Coast
4/18/2013	Spring	67.68181818	19.82323232	104	South River	Coast
4/19/2013	Spring	62.09166667	16.71759259	22	South River	Coast
4/21/2013	Spring	46.05833333	7.810185183	32	South River	Coast
4/22/2013	Spring	51.36666667	10.75925926	18	South River	Coast
4/23/2013	Spring	53.225	11.79166667	66	South River	Coast
4/24/2013	Spring	63.62727273	17.57070707	314	South River	Coast
4/25/2013	Spring	52.58333333	11.43518519	139	South River	Coast
4/26/2013	Spring	55.97272727	13.31818182	138	South River	Coast
4/27/2013	Spring	58.48333333	14.71296296	353	South River	Coast
4/28/2013	Spring	63.50909091	17.50505051	194	South River	Coast
4/29/2013	Spring	62.01666667	16.67592593	307	South River	Coast
4/30/2013	Spring	59.8	15.44444444	250	South River	Coast
5/1/2013	Spring	61.18181818	16.21212121	90	South River	Coast
5/2/2013	Spring	57.7	14.27777778	105	South River	Coast
5/3/2013	Spring	53.98181818	12.21212121	176	South River	Coast
5/4/2013	Spring	56.57272727	13.65151515	251	South River	Coast
5/5/2013	Spring	62.05454545	16.6969697	309	South River	Coast
5/9/2013	Spring	64.00909091	17.78282828	330	South River	Coast
5/10/2013	Spring	69.45454545	20.80808081	164	South River	Coast
5/11/2013	Spring	70.44545455	21.35858586	158	South River	Coast
5/12/2013	Spring	59.7	15.38888889	207	South River	Coast
5/13/2013	Spring	48.91818182	9.398989899	65	South River	Coast
5/14/2013	Spring	59.02727273	15.01515152	130	South River	Coast
5/15/2013	Spring	68.83636364	20.46464646	236	South River	Coast
5/16/2013	Spring	71.01818182	21.67676768	208	South River	Coast
5/17/2013	Spring	70.54545455	21.41414141	167	South River	Coast
5/18/2013	Spring	69.64545455	20.91414141	147	South River	Coast
5/19/2013	Spring	70.29090909	21.27272727	101	South River	Coast

5/20/2013	Spring	69.16	20.64444444	101	South River	Coast
5/21/2013	Spring	68.9	20.5	90	South River	Coast
5/22/2013	Spring	70.14545455	21.19191919	44	South River	Coast
5/23/2013	Spring	67.96363636	19.97979798	114	South River	Coast
5/24/2013	Spring	53.25454545	11.80808081	62	South River	Coast
5/25/2013	Spring	57.9	14.38888889	101	South River	Coast
5/26/2013	Spring	55.54545455	13.08080808	77	South River	Coast
5/27/2013	Spring	63.19090909	17.32828283	108	South River	Coast
5/28/2013	Spring	66.32727273	19.07070707	52	South River	Coast
5/29/2013	Spring	65.4	18.55555556	58	South River	Coast
5/30/2013	Spring	68.97272727	20.54040404	12	South River	Coast
5/31/2013	Spring	70.45454545	21.36363636	13	South River	Coast
6/1/2013	Summer	70.33636364	21.2979798	37	South River	Coast
6/2/2013	Summer	74.51818182	23.62121212	15	South River	Coast
6/3/2013	Summer	69.77272727	20.98484848	12	South River	Coast
6/4/2013	Summer	69.93636364	21.07575758	7	South River	Coast
6/5/2013	Summer	71.25454545	21.80808081	3	South River	Coast
6/6/2013	Summer	72.9	22.72222222	1	South River	Coast
6/7/2013	Summer	73.11	22.83888889	21	South River	Coast
6/8/2013	Summer	74.66	23.7	10	South River	Coast
6/9/2013	Summer	73.67	23.15	5	South River	Coast
6/11/2013	Summer	70.72	21.51111111	8	South River	Coast
6/12/2013	Summer	78.58	25.87777778	10	South River	Coast
6/13/2013	Summer	70.11111111	21.17283951	28	South River	Coast
6/14/2013	Summer	64.64	18.13333333	83	South River	Coast
6/15/2013	Summer	67.12	19.51111111	74	South River	Coast
6/16/2013	Summer	70.84	21.57777778	156	South River	Coast
6/17/2013	Summer	71.42	21.9	132	South River	Coast
6/18/2013	Summer	72.2	22.33333333	173	South River	Coast
6/19/2013	Summer	67.67	19.81666667	153	South River	Coast
6/20/2013	Summer	64.9	18.27777778	172	South River	Coast
6/21/2013	Summer	71.64	22.02222222	61	South River	Coast
6/22/2013	Summer	73.38	22.98888889	32	South River	Coast

6/23/2013	Summer	74.69	23.71666667	31	South River	Coast
6/24/2013	Summer	73.53	23.07222222	26	South River	Coast
6/25/2013	Summer	72.81	22.67222222	5	South River	Coast
6/26/2013	Summer	71.01	21.67222222	12	South River	Coast
6/27/2013	Summer	75.46	24.14444444	7	South River	Coast
6/28/2013	Summer	79.01	26.11666667	9	South River	Coast
6/29/2013	Summer	74.25	23.47222222	6	South River	Coast
6/30/2013	Summer	74.08	23.37777778	25	South River	Coast
7/1/2013	Summer	73.75	23.19444444	14	South River	Coast
7/2/2013	Summer	74.38	23.54444444	22	South River	Coast
7/3/2013	Summer	74.52	23.62222222	31	South River	Coast
7/4/2013	Summer	73.5	23.05555556	40	South River	Coast
7/5/2013	Summer	72.97	22.76111111	53	South River	Coast
7/6/2013	Summer	72.24	22.35555556	94	South River	Coast
7/7/2013	Summer	74.34444444	23.52469136	324	South River	Coast
7/8/2013	Summer	76.59	24.77222222	292	South River	Coast
7/9/2013	Summer	76.56666667	24.75925926	483	South River	Coast
7/10/2013	Summer	75.86	24.36666667	568	South River	Coast
7/12/2013	Summer	72.23	22.35	191	South River	Coast
7/13/2013	Summer	73.17	22.87222222	194	South River	Coast
7/14/2013	Summer	74.16	23.42222222	351	South River	Coast
7/15/2013	Summer	74.03	23.35	537	South River	Coast
7/16/2013	Summer	78.02	25.56666667	935	South River	Coast
7/17/2013	Summer	76.75	24.86111111	685	South River	Coast
7/18/2013	Summer	77.32	25.17777778	637	South River	Coast
7/19/2013	Summer	76.99	24.99444444	801	South River	Coast
7/20/2013	Summer	75.51	24.17222222	1197	South River	Coast
7/21/2013	Summer	76.01	24.45	828	South River	Coast
7/24/2013	Summer	71.00909091	21.67171717	328	South River	Coast
7/25/2013	Summer	71.3	21.83333333	270	South River	Coast
7/26/2013	Summer	71.57272727	21.98484848	193	South River	Coast
7/27/2013	Summer	74.85454545	23.80808081	221	South River	Coast
7/28/2013	Summer	77.23	25.12777778	175	South River	Coast

7/29/2013	Summer	70.88181818	21.6010101	107	South River	Coast
7/30/2013	Summer	73.55454545	23.08585859	93	South River	Coast
7/31/2013	Summer	75.05454545	23.91919192	85	South River	Coast
8/1/2013	Summer	74.98333333	23.87962963	82	South River	Coast
8/2/2013	Summer	76.85833333	24.9212963	121	South River	Coast
8/3/2013	Summer	76.425	24.68055556	64	South River	Coast
8/4/2013	Summer	69.66666667	20.92592593	85	South River	Coast
8/5/2013	Summer	70.95	21.63888889	88	South River	Coast
8/6/2013	Summer	70.29166667	21.27314815	64	South River	Coast
8/7/2013	Summer	73.24166667	22.91203704	72	South River	Coast
8/8/2013	Summer	77.55	25.30555556	59	South River	Coast
8/9/2013	Summer	78.10833333	25.61574074	66	South River	Coast
8/10/2013	Summer	78.8	26	91	South River	Coast
8/12/2013	Summer	78.34545455	25.74747475	55	South River	Coast
8/13/2013	Summer	75.60833333	24.22685185	76	South River	Coast
8/14/2013	Summer	65.88333333	18.82407407	52	South River	Coast
8/15/2013	Summer	67.70833333	19.83796296	69	South River	Coast
8/16/2013	Summer	66.63333333	19.24074074	46	South River	Coast
8/17/2013	Summer	72.54166667	22.52314815	61	South River	Coast
8/18/2013	Summer	75.10833333	23.94907407	98	South River	Coast
8/19/2013	Summer	73.89166667	23.27314815	89	South River	Coast
8/20/2013	Summer	73.01666667	22.78703704	18	South River	Coast
8/21/2013	Summer	71.56666667	21.98148148	75	South River	Coast
8/22/2013	Summer	71.8	22.11111111	349	South River	Coast
9/2/2013	Fall	76.50833333	24.72685185	291	South River	Coast
9/3/2013	Fall	69.75	20.97222222	176	South River	Coast
9/4/2013	Fall	75.825	24.34722222	173	South River	Coast
9/5/2013	Fall	70.45833333	21.36574074	184	South River	Coast
9/6/2013	Fall	68.16666667	20.09259259	237	South River	Coast
9/7/2013	Fall	67.54166667	19.74537037	306	South River	Coast
9/8/2013	Fall	69.28333333	20.71296296	148	South River	Coast
9/9/2013	Fall	70.75	21.52777778	189	South River	Coast
9/10/2013	Fall	70.08333333	21.15740741	208	South River	Coast

9/11/2013	Fall	69.75	20.97222222	234	South River	Coast
9/12/2013	Fall	74.025	23.34722222	192	South River	Coast
9/13/2013	Fall	69.66666667	20.92592593	133	South River	Coast
9/14/2013	Fall	63.73846154	17.63247863	93	South River	Coast
9/15/2013	Fall	69.16153846	20.64529915	88	South River	Coast
9/16/2013	Fall	69.69230769	20.94017094	218	South River	Coast
9/17/2013	Fall	59.59230769	15.32905983	125	South River	Coast
9/18/2013	Fall	58.60769231	14.78205128	105	South River	Coast
9/19/2013	Fall	65.86923077	18.81623932	218	South River	Coast
9/20/2013	Fall	66.99230769	19.44017094	55	South River	Coast
9/21/2013	Fall	72.01538462	22.23076923	15	South River	Coast
9/22/2013	Fall	61.85384615	16.58547009	73	South River	Coast
9/23/2013	Fall	57.39230769	14.10683761	71	South River	Coast
9/24/2013	Fall	58.44615385	14.69230769	69	South River	Coast
9/25/2013	Fall	63.39230769	17.44017094	25	South River	Coast
9/26/2013	Fall	60.53076923	15.85042735	69	South River	Coast
9/27/2013	Fall	59.34615385	15.19230769	39	South River	Coast
9/28/2013	Fall	58.81538462	14.8974359	73	South River	Coast
9/29/2013	Fall	59.07692308	15.04273504	38	South River	Coast
9/30/2013	Fall	56.68461538	13.71367521	24	South River	Coast
10/1/2013	Fall	63.13076923	17.29487179	37	South River	Coast
10/2/2013	Fall	65.93076923	18.85042735	27	South River	Coast
10/3/2013	Fall	66.775	19.31944444	28	South River	Coast
10/4/2013	Fall	67.48461538	19.71367521	55	South River	Coast
10/5/2013	Fall	67.86153846	19.92307692	36	South River	Coast
10/6/2013	Fall	72.4	22.44444444	62	South River	Coast
10/7/2013	Fall	68.20769231	20.11538462	44	South River	Coast
10/8/2013	Fall	57.83846154	14.35470085	14	South River	Coast
11/13/2013	Fall	30.52142857	-0.821428571	1	South River	Coast
11/14/2013	Fall	37.18	2.877777778	0	South River	Coast
11/15/2013	Fall	59.08	15.04444444	123	South River	Coast
11/16/2013	Fall	57.94	14.41111111	72	South River	Coast
11/17/2013	Fall	69.19333333	20.66296296	28	South River	Coast

11/19/2013	Fall	43.12857143	6.182539683	36	South River	Coast
11/20/2013	Fall	46.74666667	8.192592593	27	South River	Coast
11/21/2013	Fall	57.62	14.23333333	84	South River	Coast
11/22/2013	Fall	63.8	17.66666667	92	South River	Coast
11/23/2013	Fall	48.24666667	9.025925926	241	South River	Coast
4/5/2014	Spring	57.46666667	14.14814815	18	South River	Coast
4/6/2014	Spring	55.63333333	13.12962963	7	South River	Coast
4/8/2014	Spring	55.80833333	13.22685185	14	South River	Coast
4/9/2014	Spring	49.61666667	9.787037037	3	South River	Coast
4/10/2014	Spring	57.25833333	14.03240741	8	South River	Coast
4/11/2014	Spring	61.13333333	16.18518519	10	South River	Coast
4/12/2014	Spring	60.48333333	15.82407407	8	South River	Coast
4/13/2014	Spring	61.225	16.23611111	9	South River	Coast
4/14/2014	Spring	69.21666667	20.67592593	28	South River	Coast
4/15/2014	Spring	43.48333333	6.37962963	2	South River	Coast
4/16/2014	Spring	39.15833333	3.976851852	7	South River	Coast
4/17/2014	Spring	48.55833333	9.199074074	10	South River	Coast
4/18/2014	Spring	52.125	11.18055556	0	South River	Coast
4/19/2014	Spring	49.475	9.708333333	4	South River	Coast
4/20/2014	Spring	52.725	11.51388889	22	South River	Coast
4/21/2014	Spring	53.90833333	12.1712963	34	South River	Coast
4/22/2014	Spring	62.56666667	16.98148148	62	South River	Coast
4/24/2014	Spring	56.49166667	13.60648148	24	South River	Coast
4/25/2014	Spring	64.91666667	18.28703704	90	South River	Coast
4/26/2014	Spring	60.17272727	15.65151515	45	South River	Coast
4/27/2014	Spring	64.11666667	17.84259259	47	South River	Coast
4/28/2014	Spring	65.11666667	18.39814815	12	South River	Coast
4/29/2014	Spring	72.06666667	22.25925926	24	South River	Coast
4/30/2014	Spring	69.75454545	20.97474747	17	South River	Coast
5/1/2014	Spring	64.92727273	18.29292929	68	South River	Coast
5/2/2014	Spring	57.93636364	14.40909091	23	South River	Coast
5/3/2014	Spring	60.54545455	15.85858586	37	South River	Coast
5/4/2014	Spring	66.82727273	19.34848485	96	South River	Coast

5/5/2014	Spring	61.36363636	16.31313131	81	South River	Coast
5/6/2014	Spring	67.10909091	19.50505051	117	South River	Coast
5/7/2014	Spring	69.38181818	20.76767677	30	South River	Coast
5/8/2014	Spring	70.83636364	21.57575758	67	South River	Coast
5/9/2014	Spring	72.16363636	22.31313131	31	South River	Coast
5/10/2014	Spring	71.90909091	22.17171717	27	South River	Coast
5/11/2014	Spring	69.84545455	21.02525253	14	South River	Coast
5/12/2014	Spring	71.37272727	21.87373737	48	South River	Coast
5/13/2014	Spring	70.8	21.55555556	18	South River	Coast
5/14/2014	Spring	68.42727273	20.23737374	21	South River	Coast
5/15/2014	Spring	69.50909091	20.83838384	1	South River	Coast
5/16/2014	Spring	58.59090909	14.77272727	43	South River	Coast
5/17/2014	Spring	60.77272727	15.98484848	98	South River	Coast
5/18/2014	Spring	54.29090909	12.38383838	73	South River	Coast
5/19/2014	Spring	54.88181818	12.71212121	72	South River	Coast
5/20/2014	Spring	64.12727273	17.84848485	31	South River	Coast
5/21/2014	Spring	72.04545455	22.24747475	15	South River	Coast
5/22/2014	Spring	72.5	22.5	8	South River	Coast
5/23/2014	Spring	71.38181818	21.87878788	22	South River	Coast
5/24/2014	Spring	61.69090909	16.49494949	35	South River	Coast
5/25/2014	Spring	66.37272727	19.0959596	39	South River	Coast
5/26/2014	Spring	71.35454545	21.86363636	22	South River	Coast
5/27/2014	Spring	70.21818182	21.23232323	11	South River	Coast
5/28/2014	Spring	74.66363636	23.7020202	11	South River	Coast
5/29/2014	Spring	69.30909091	20.72727273	13	South River	Coast
5/30/2014	Spring	68.66363636	20.36868687	1	South River	Coast
5/31/2014	Spring	63.97272727	17.76262626	36	South River	Coast
6/1/2014	Summer	59.32727273	15.18181818	19	South River	Coast
6/2/2014	Summer	65.30909091	18.50505051	21	South River	Coast
6/3/2014	Summer	72.32727273	22.4040404	7	South River	Coast
6/4/2014	Summer	76.4	24.66666667	7	South River	Coast
6/5/2014	Summer	73	22.77777778	38	South River	Coast
6/6/2014	Summer	68.72	20.4	8	South River	Coast

6/7/2014	Summer	66.73	19.29444444	0	South River	Coast
6/8/2014	Summer	69.21	20.67222222	14	South River	Coast
6/9/2014	Summer	75.58	24.21111111	10	South River	Coast
6/10/2014	Summer	76.82222222	24.90123457	3	South River	Coast
6/11/2014	Summer	73.46	23.03333333	33	South River	Coast
6/12/2014	Summer	70.2	21.22222222	6	South River	Coast
6/13/2014	Summer	75.08	23.93333333	11	South River	Coast
6/14/2014	Summer	73.48	23.04444444	9	South River	Coast
6/15/2014	Summer	71.43	21.90555556	0	South River	Coast
6/16/2014	Summer	74.13	23.40555556	10	South River	Coast
6/17/2014	Summer	73.99	23.32777778	11	South River	Coast
6/18/2014	Summer	75.82	24.34444444	7	South River	Coast
6/19/2014	Summer	75.28	24.04444444	11	South River	Coast
6/20/2014	Summer	73.67	23.15	3	South River	Coast
6/21/2014	Summer	71.82	22.12222222	2	South River	Coast
6/22/2014	Summer	71.25	21.80555556	7	South River	Coast
6/23/2014	Summer	72.3	22.38888889	0	South River	Coast
6/24/2014	Summer	74.99	23.88333333	7	South River	Coast
6/25/2014	Summer	77.27	25.15	59	South River	Coast
6/26/2014	Summer	74.73	23.73888889	14	South River	Coast
6/27/2014	Summer	71.71	22.06111111	16	South River	Coast
6/28/2014	Summer	69.77	20.98333333	3	South River	Coast
6/29/2014	Summer	69.87	21.03888889	34	South River	Coast
6/30/2014	Summer	71.99	22.21666667	30	South River	Coast
7/1/2014	Summer	75.58	24.21111111	40	South River	Coast
7/2/2014	Summer	76.95	24.97222222	48	South River	Coast
7/3/2014	Summer	74.93	23.85	21	South River	Coast
7/4/2014	Summer	73.98	23.32222222	39	South River	Coast
7/5/2014	Summer	67.2	19.55555556	16	South River	Coast
7/6/2014	Summer	68.91	20.50555556	44	South River	Coast
7/7/2014	Summer	76.19	24.55	33	South River	Coast
7/8/2014	Summer	78.48	25.82222222	40	South River	Coast
7/9/2014	Summer	78.12	25.62222222	27	South River	Coast

7/10/2014	Summer	70.36	21.31111111	3	South River	Coast
7/11/2014	Summer	72.66	22.58888889	50	South River	Coast
7/12/2014	Summer	72.9	22.72222222	57	South River	Coast
7/13/2014	Summer	75.33	24.07222222	73	South River	Coast
7/14/2014	Summer	78.49	25.82777778	72	South River	Coast
7/15/2014	Summer	72.54	22.52222222	8	South River	Coast
7/16/2014	Summer	69.7	20.94444444	40	South River	Coast
7/17/2014	Summer	69.44	20.8	42	South River	Coast
7/18/2014	Summer	69.41111111	20.78395062	60	South River	Coast
7/19/2014	Summer	71.68888889	22.04938272	47	South River	Coast
7/20/2014	Summer	74.8	23.77777778	27	South River	Coast
7/21/2014	Summer	72.47777778	22.48765432	20	South River	Coast
7/22/2014	Summer	75.13	23.96111111	39	South River	Coast
7/23/2014	Summer	77.16	25.08888889	36	South River	Coast
7/24/2014	Summer	72.61818182	22.56565657	34	South River	Coast
7/25/2014	Summer	74.64545455	23.69191919	49	South River	Coast
7/26/2014	Summer	78.10909091	25.61616162	47	South River	Coast
7/27/2014	Summer	83.10909091	28.39393939	56	South River	Coast
7/28/2014	Summer	71.94545455	22.19191919	69	South River	Coast
7/29/2014	Summer	68.77272727	20.42929293	121	South River	Coast
7/31/2014	Summer	73.03636364	22.7979798	37	South River	Coast
8/1/2014	Summer	71.60833333	22.00462963	47	South River	Coast
8/2/2014	Summer	70.89166667	21.60648148	11	South River	Coast
8/3/2014	Summer	70.81666667	21.56481481	85	South River	Coast
8/4/2014	Summer	73.64545455	23.13636364	62	South River	Coast
8/5/2014	Summer	74.45	23.58333333	61	South River	Coast
8/6/2014	Summer	74.46666667	23.59259259	60	South River	Coast
8/7/2014	Summer	71.96666667	22.2037037	97	South River	Coast
8/8/2014	Summer	74.46666667	23.59259259	168	South River	Coast
8/9/2014	Summer	70.88333333	21.60185185	83	South River	Coast
8/10/2014	Summer	72.08333333	22.26851852	124	South River	Coast
8/12/2014	Summer	76.75833333	24.86574074	144	South River	Coast
8/13/2014	Summer	69.51666667	20.84259259	205	South River	Coast

8/14/2014	Summer	67.075	19.48611111	186	South River	Coast
8/15/2014	Summer	71.9	22.16666667	221	South River	Coast
8/16/2014	Summer	74.075	23.375	139	South River	Coast
8/17/2014	Summer	75.29166667	24.05092593	104	South River	Coast
8/18/2014	Summer	75.1	23.94444444	119	South River	Coast
8/19/2014	Summer	73.80833333	23.22685185	65	South River	Coast
8/20/2014	Summer	76.95833333	24.97685185	79	South River	Coast
8/21/2014	Summer	77.88333333	25.49074074	105	South River	Coast
8/22/2014	Summer	74.525	23.625	78	South River	Coast
8/23/2014	Summer	73.34166667	22.96759259	143	South River	Coast
8/24/2014	Summer	65.14166667	18.41203704	222	South River	Coast
8/25/2014	Summer	66.80833333	19.33796296	203	South River	Coast
8/26/2014	Summer	66.23333333	19.01851852	270	South River	Coast
8/27/2014	Summer	66.65	19.25	216	South River	Coast
8/28/2014	Summer	74.10833333	23.39351852	204	South River	Coast
8/29/2014	Summer	73.325	22.95833333	150	South River	Coast
8/30/2014	Summer	73.23333333	22.90740741	106	South River	Coast
8/31/2014	Summer	76.16666667	24.53703704	167	South River	Coast
6/11/2013	Summer	65.59	18.66111111	608	uwharrie	peidmont
6/12/2013	Summer	74.54	23.63333333	581	uwharrie	peidmont
6/15/2013	Summer	64.6	18.11111111	815	uwharrie	peidmont
6/16/2013	Summer	70.14	21.18888889	1084	uwharrie	peidmont
6/17/2013	Summer	69.46	20.81111111	705	uwharrie	peidmont
6/18/2013	Summer	67.91	19.95	545	uwharrie	peidmont
6/19/2013	Summer	66.07	18.92777778	478	uwharrie	peidmont
6/20/2013	Summer	62.58	16.98888889	211	uwharrie	peidmont
6/21/2013	Summer	66.18	18.98888889	224	uwharrie	peidmont
6/22/2013	Summer	68.75	20.41666667	205	uwharrie	peidmont
6/23/2013	Summer	71.72	22.06666667	201	uwharrie	peidmont
6/24/2013	Summer	69.26	20.7	99	uwharrie	peidmont
6/25/2013	Summer	70.25	21.25	56	uwharrie	peidmont
6/26/2013	Summer	67.98	19.98888889	308	uwharrie	peidmont
6/27/2013	Summer	71.55	21.97222222	118	uwharrie	peidmont

6/28/2013	Summer	70.06	21.14444444	92	uwharrie	peidmont
6/29/2013	Summer	74.27	23.48333333	265	uwharrie	peidmont
6/30/2013	Summer	69.1	20.61111111	304	uwharrie	peidmont
7/1/2013	Summer	71.82	22.12222222	851	uwharrie	peidmont
7/2/2013	Summer	72.79	22.66111111	493	uwharrie	peidmont
7/3/2013	Summer	73.27	22.92777778	336	uwharrie	peidmont
7/4/2013	Summer	72.61	22.56111111	655	uwharrie	peidmont
7/5/2013	Summer	71.83	22.12777778	372	uwharrie	peidmont
7/6/2013	Summer	71.32	21.84444444	416	uwharrie	peidmont
7/7/2013	Summer	71.31	21.83888889	368	uwharrie	peidmont
7/8/2013	Summer	73.17	22.87222222	440	uwharrie	peidmont
7/9/2013	Summer	72.68	22.6	644	uwharrie	peidmont
7/10/2013	Summer	73.69	23.16111111	466	uwharrie	peidmont
7/11/2013	Summer	71.75	22.08333333	497	uwharrie	peidmont
7/12/2013	Summer	70.24	21.24444444	806	uwharrie	peidmont
7/13/2013	Summer	72.78	22.65555556	655	uwharrie	peidmont
7/14/2013	Summer	70.06	21.14444444	746	uwharrie	peidmont
7/16/2013	Summer	74.67	23.70555556	700	uwharrie	peidmont
7/17/2013	Summer	72.71	22.61666667	794	uwharrie	peidmont
7/18/2013	Summer	74.14	23.41111111	519	uwharrie	peidmont
7/19/2013	Summer	74.56	23.64444444	562	uwharrie	peidmont
7/20/2013	Summer	73.66	23.14444444	494	uwharrie	peidmont
7/21/2013	Summer	72.31	22.39444444	449	uwharrie	peidmont
7/22/2013	Summer	73.56	23.08888889	259	uwharrie	peidmont
7/23/2013	Summer	72.03	22.23888889	157	uwharrie	peidmont
7/24/2013	Summer	68.55	20.30555556	275	uwharrie	peidmont
7/25/2013	Summer	66.80909091	19.33838384	189	uwharrie	peidmont
7/26/2013	Summer	70.89090909	21.60606061	671	uwharrie	peidmont
7/27/2013	Summer	69.42727273	20.79292929	527	uwharrie	peidmont
7/28/2013	Summer	71.17272727	21.76262626	450	uwharrie	peidmont
7/29/2013	Summer	66.08181818	18.93434343	594	uwharrie	peidmont
7/30/2013	Summer	70.59090909	21.43939394	538	uwharrie	peidmont
7/31/2013	Summer	71.35454545	21.86363636	1390	uwharrie	peidmont

8/1/2013	Summer	72.18181818	22.32323232	864	uwharrie	peidmont
8/2/2013	Summer	73.53333333	23.07407407	1275	uwharrie	peidmont
8/3/2013	Summer	71.26666667	21.81481481	753	uwharrie	peidmont
8/4/2013	Summer	67.375	19.65277778	585	uwharrie	peidmont
8/5/2013	Summer	69.60833333	20.89351852	1167	uwharrie	peidmont
8/6/2013	Summer	68.79166667	20.43981481	611	uwharrie	peidmont
8/7/2013	Summer	71.93333333	22.18518519	614	uwharrie	peidmont
9/10/2013	Fall	67.23333333	19.57407407	126	uwharrie	peidmont
9/11/2013	Fall	69.73333333	20.96296296	72	uwharrie	peidmont
9/12/2013	Fall	68.99166667	20.55092593	32	uwharrie	peidmont
9/13/2013	Fall	62.30833333	16.83796296	61	uwharrie	peidmont
9/14/2013	Fall	57.21538462	14.00854701	21	uwharrie	peidmont
9/15/2013	Fall	67.60769231	19.78205128	641	uwharrie	peidmont
9/16/2013	Fall	67.01538462	19.45299145	46	uwharrie	peidmont
9/17/2013	Fall	54.93846154	12.74358974	36	uwharrie	peidmont
9/18/2013	Fall	54.56153846	12.53418803	18	uwharrie	peidmont
9/19/2013	Fall	63.53846154	17.52136752	40	uwharrie	peidmont
9/20/2013	Fall	66.66923077	19.26068376	268	uwharrie	peidmont
9/21/2013	Fall	64.87692308	18.26495726	58	uwharrie	peidmont
9/22/2013	Fall	56.46923077	13.59401709	36	uwharrie	peidmont
9/23/2013	Fall	54.86923077	12.70512821	29	uwharrie	peidmont
9/24/2013	Fall	56.23846154	13.46581197	55	uwharrie	peidmont
9/25/2013	Fall	60.47692308	15.82051282	29	uwharrie	peidmont
9/26/2013	Fall	55.64615385	13.13675214	31	uwharrie	peidmont
9/27/2013	Fall	54.09230769	12.27350427	41	uwharrie	peidmont
9/28/2013	Fall	53.81538462	12.11965812	44	uwharrie	peidmont
9/29/2013	Fall	52.03076923	11.12820513	23	uwharrie	peidmont
9/30/2013	Fall	52.90769231	11.61538462	42	uwharrie	peidmont
10/1/2013	Fall	57.85384615	14.36324786	96	uwharrie	peidmont
10/2/2013	Fall	59.12307692	15.06837607	90	uwharrie	peidmont
10/3/2013	Fall	61.53076923	16.40598291	151	uwharrie	peidmont
10/4/2013	Fall	62.22307692	16.79059829	143	uwharrie	peidmont
10/5/2013	Fall	63.88461538	17.71367521	197	uwharrie	peidmont

10/6/2013	Fall	70.07692308	21.15384615	1419	uwharrie	peidmont
10/7/2013	Fall	62.10769231	16.72649573	32	uwharrie	peidmont
10/8/2013	Fall	55.20769231	12.89316239	48	uwharrie	peidmont
10/9/2013	Fall	56.97692308	13.87606838	50	uwharrie	peidmont
10/10/2013	Fall	60.46153846	15.81196581	165	uwharrie	peidmont
10/11/2013	Fall	61.84615385	16.58119658	103	uwharrie	peidmont
10/12/2013	Fall	63.76153846	17.64529915	291	uwharrie	peidmont
10/13/2013	Fall	62.23076923	16.79487179	85	uwharrie	peidmont
10/14/2013	Fall	57.16923077	13.98290598	61	uwharrie	peidmont
10/15/2013	Fall	61.06153846	16.14529915	41	uwharrie	peidmont
10/16/2013	Fall	61.21538462	16.23076923	56	uwharrie	peidmont
10/17/2013	Fall	63.14615385	17.3034188	50	uwharrie	peidmont
10/18/2013	Fall	58.49230769	14.71794872	17	uwharrie	peidmont
10/19/2013	Fall	52.77142857	11.53968254	42	uwharrie	peidmont
10/20/2013	Fall	42.56428571	5.869047619	12	uwharrie	peidmont
10/21/2013	Fall	53.15	11.75	27	uwharrie	peidmont
10/22/2013	Fall	57.43571429	14.13095238	50	uwharrie	peidmont
10/23/2013	Fall	40.24285714	4.579365079	17	uwharrie	peidmont
10/24/2013	Fall	43.3	6.277777778	4	uwharrie	peidmont
10/25/2013	Fall	31.97857143	-0.011904762	0	uwharrie	peidmont
10/26/2013	Fall	41.73571429	5.408730159	1	uwharrie	peidmont
10/27/2013	Fall	45.38571429	7.436507937	31	uwharrie	peidmont
10/28/2013	Fall	48.42142857	9.123015873	27	uwharrie	peidmont
10/29/2013	Fall	51.22666667	10.68148148	52	uwharrie	peidmont
10/30/2013	Fall	55.47333333	13.04074074	62	uwharrie	peidmont
10/31/2013	Fall	67.07333333	19.48518519	1070	uwharrie	peidmont
11/1/2013	Fall	57.84	14.35555556	94	uwharrie	peidmont
11/2/2013	Fall	46.55	8.083333333	25	uwharrie	peidmont
11/26/2013	Fall	49.40666667	9.67037037	1	uwharrie	peidmont
11/27/2013	Fall	27.92	-2.266666667	1	uwharrie	peidmont
11/28/2013	Fall	26.72	-2.933333333	0	uwharrie	peidmont
11/29/2013	Fall	30.74666667	-0.696296296	1	uwharrie	peidmont
11/30/2013	Fall	31.58666667	-0.22962963	0	uwharrie	peidmont

12/1/2013	Winter	42.6	5.888888889	2	uwharrie	peidmont
12/2/2013	Winter	41.85333333	5.474074074	3	uwharrie	peidmont
12/3/2013	Winter	51.99333333	11.10740741	706	uwharrie	peidmont
12/4/2013	Winter	57.46	14.14444444	160	uwharrie	peidmont
12/5/2013	Winter	64.03333333	17.7962963	153	uwharrie	peidmont
12/6/2013	Winter	65.76666667	18.75925926	225	uwharrie	peidmont
12/7/2013	Winter	43.79333333	6.551851852	1	uwharrie	peidmont
12/8/2013	Winter	34.22	1.233333333	0	uwharrie	peidmont
12/9/2013	Winter	44.89333333	7.162962963	0	uwharrie	peidmont
12/10/2013	Winter	30.46666667	-0.851851852	0	uwharrie	peidmont
12/11/2013	Winter	28.1	-2.166666667	0	uwharrie	peidmont
12/12/2013	Winter	25.06666667	-3.851851852	0	uwharrie	peidmont
12/13/2013	Winter	35.28666667	1.825925926	0	uwharrie	peidmont
12/14/2013	Winter	41.78666667	5.437037037	0	uwharrie	peidmont
12/15/2013	Winter	33.38	0.766666667	2	uwharrie	peidmont
12/16/2013	Winter	36.86666667	2.703703704	1	uwharrie	peidmont
12/17/2013	Winter	44.76666667	7.092592593	4	uwharrie	peidmont
12/18/2013	Winter	29.27333333	-1.514814815	0	uwharrie	peidmont
12/19/2013	Winter	43.66	6.477777778	31	uwharrie	peidmont
12/20/2013	Winter	49.63333333	9.796296296	147	uwharrie	peidmont
12/21/2013	Winter	68.23333333	20.12962963	35	uwharrie	peidmont
12/22/2013	Winter	64.12666667	17.84814815	0	uwharrie	peidmont
12/23/2013	Winter	47.20666667	8.448148148	1	uwharrie	peidmont
12/24/2013	Winter	29.21333333	-1.548148148	0	uwharrie	peidmont
12/25/2013	Winter	30.08	-1.066666667	0	uwharrie	peidmont
12/26/2013	Winter	30.62	-0.766666667	0	uwharrie	peidmont
12/27/2013	Winter	28.41333333	-1.992592593	0	uwharrie	peidmont
12/28/2013	Winter	44.43333333	6.907407407	0	uwharrie	peidmont
12/29/2013	Winter	38.48	3.6	0	uwharrie	peidmont
12/30/2013	Winter	41.16	5.088888889	0	uwharrie	peidmont
12/31/2013	Winter	31.90666667	-0.051851852	1	uwharrie	peidmont
1/1/2014	Winter	37.12666667	2.848148148	0	uwharrie	peidmont
1/2/2014	Winter	37.68	3.155555556	4	uwharrie	peidmont

1/3/2014	Winter	20.23333333	-6.537037037	0	uwharrie	peidmont
1/4/2014	Winter	33.64	0.911111111	2	uwharrie	peidmont
1/5/2014	Winter	47.46666667	8.592592593	2	uwharrie	peidmont
1/6/2014	Winter	17.51333333	-8.048148148	0	uwharrie	peidmont
1/7/2014	Winter	15.64666667	-9.085185185	0	uwharrie	peidmont
1/8/2014	Winter	24.76	-4.022222222	0	uwharrie	peidmont
1/9/2014	Winter	44.02	6.677777778	3	uwharrie	peidmont
1/10/2014	Winter	44.12666667	6.737037037	1	uwharrie	peidmont
1/11/2014	Winter	49.59333333	9.774074074	3	uwharrie	peidmont
1/12/2014	Winter	34.58666667	1.437037037	2	uwharrie	peidmont
1/13/2014	Winter	47.77333333	8.762962963	10	uwharrie	peidmont
1/14/2014	Winter	37.64666667	3.137037037	2	uwharrie	peidmont
1/15/2014	Winter	36.35714286	2.420634921	1	uwharrie	peidmont
1/16/2014	Winter	30.36428571	-0.908730159	0	uwharrie	peidmont
1/17/2014	Winter	38.40714286	3.55952381	0	uwharrie	peidmont
1/18/2014	Winter	30.54285714	-0.80952381	0	uwharrie	peidmont
1/19/2014	Winter	32.62857143	0.349206349	0	uwharrie	peidmont
1/20/2014	Winter	34.51428571	1.396825397	5	uwharrie	peidmont
1/21/2014	Winter	30.13571429	-1.035714286	0	uwharrie	peidmont
1/22/2014	Winter	17.56428571	-8.01984127	0	uwharrie	peidmont
1/23/2014	Winter	22.70714286	-5.162698413	0	uwharrie	peidmont
1/24/2014	Winter	16.57692308	-8.568376068	0	uwharrie	peidmont
1/25/2014	Winter	31.66428571	-0.186507937	0	uwharrie	peidmont
1/26/2014	Winter	43.17857143	6.21031746	0	uwharrie	peidmont
1/27/2014	Winter	37.47142857	3.03968254	4	uwharrie	peidmont
1/28/2014	Winter	20.51428571	-6.380952381	0	uwharrie	peidmont
1/29/2014	Winter	13.22142857	-10.43253968	0	uwharrie	peidmont
1/30/2014	Winter	16.77857143	-8.456349206	0	uwharrie	peidmont
1/31/2014	Winter	27.85714286	-2.301587302	1	uwharrie	peidmont
2/1/2014	Winter	43.17142857	6.206349206	0	uwharrie	peidmont
2/2/2014	Winter	52.96428571	11.6468254	4	uwharrie	peidmont
2/3/2014	Winter	42.27857143	5.71031746	3	uwharrie	peidmont
2/4/2014	Winter	37.12857143	2.849206349	0	uwharrie	peidmont

2/5/2014	Winter	39.88571429	4.380952381	1	uwharrie	peidmont
2/6/2014	Winter	34.42142857	1.345238095	0	uwharrie	peidmont
2/7/2014	Winter	33.99285714	1.107142857	0	uwharrie	peidmont
2/8/2014	Winter	33.75	0.972222222	1	uwharrie	peidmont
2/9/2014	Winter	41.38571429	5.214285714	1	uwharrie	peidmont
2/10/2014	Winter	33.87142857	1.03968254	0	uwharrie	peidmont
2/11/2014	Winter	27.31428571	-2.603174603	0	uwharrie	peidmont
2/12/2014	Winter	26.32307692	-3.153846154	0	uwharrie	peidmont
2/13/2014	Winter	31.72857143	-0.150793651	0	uwharrie	peidmont
2/14/2014	Winter	39.45714286	4.142857143	0	uwharrie	peidmont
2/15/2014	Winter	31.40714286	-0.329365079	0	uwharrie	peidmont
2/16/2014	Winter	32.05714286	0.031746032	0	uwharrie	peidmont
2/17/2014	Winter	36.06428571	2.257936508	0	uwharrie	peidmont
2/18/2014	Winter	44.92857143	7.182539683	12	uwharrie	peidmont
2/19/2014	Winter	54.13571429	12.29761905	30	uwharrie	peidmont
2/20/2014	Winter	65.75	18.75	225	uwharrie	peidmont
2/21/2014	Winter	38.71428571	3.73015873	10	uwharrie	peidmont
2/22/2014	Winter	37.40714286	3.003968254	1	uwharrie	peidmont
2/23/2014	Winter	47.62857143	8.682539683	123	uwharrie	peidmont
2/24/2014	Winter	37.2	2.888888889	8	uwharrie	peidmont
2/25/2014	Winter	40.32142857	4.623015873	0	uwharrie	peidmont
2/26/2014	Winter	29.25	-1.527777778	2	uwharrie	peidmont
2/27/2014	Winter	32.15714286	0.087301587	0	uwharrie	peidmont
2/28/2014	Winter	34.64285714	1.468253968	0	uwharrie	peidmont
3/1/2014	Spring	34.49285714	1.384920635	0	uwharrie	peidmont
3/2/2014	Spring	57.27857143	14.04365079	155	uwharrie	peidmont
3/3/2014	Spring	22.8	-5.111111111	0	uwharrie	peidmont
3/4/2014	Spring	32.82142857	0.456349206	0	uwharrie	peidmont
3/5/2014	Spring	31.18571429	-0.452380952	0	uwharrie	peidmont
3/6/2014	Spring	33.62857143	0.904761905	0	uwharrie	peidmont
3/7/2014	Spring	31.84285714	-0.087301587	0	uwharrie	peidmont
3/8/2014	Spring	43.27142857	6.261904762	1	uwharrie	peidmont
3/28/2014	Spring	56.76666667	13.75925926	8	uwharrie	peidmont

3/29/2014	Spring	51.35833333	10.75462963	20	uwharrie	peidmont
3/30/2014	Spring	40.80833333	4.893518519	0	uwharrie	peidmont
3/31/2014	Spring	41.5	5.277777778	84	uwharrie	peidmont
4/1/2014	Spring	49.90833333	9.949074074	239	uwharrie	peidmont
4/2/2014	Spring	56.675	13.70833333	415	uwharrie	peidmont
4/3/2014	Spring	62.73333333	17.07407407	304	uwharrie	peidmont
4/4/2014	Spring	63.375	17.43055556	844	uwharrie	peidmont
4/5/2014	Spring	50.15	10.08333333	79	uwharrie	peidmont
4/6/2014	Spring	51.65833333	10.9212963	5	uwharrie	peidmont
4/7/2014	Spring	60.73333333	15.96296296	99	uwharrie	peidmont
4/8/2014	Spring	48.65	9.25	18	uwharrie	peidmont
4/9/2014	Spring	40.26666667	4.592592593	14	uwharrie	peidmont
4/10/2014	Spring	52.19166667	11.21759259	97	uwharrie	peidmont
4/11/2014	Spring	56.64166667	13.68981481	299	uwharrie	peidmont
4/12/2014	Spring	61.21666667	16.23148148	249	uwharrie	peidmont
4/13/2014	Spring	64.54166667	18.0787037	150	uwharrie	peidmont
4/14/2014	Spring	66.04166667	18.91203704	290	uwharrie	peidmont
4/15/2014	Spring	36.28333333	2.37962963	0	uwharrie	peidmont
4/16/2014	Spring	34.76666667	1.537037037	0	uwharrie	peidmont
4/17/2014	Spring	42.66666667	5.925925926	5	uwharrie	peidmont
4/18/2014	Spring	48.81666667	9.342592593	0	uwharrie	peidmont
4/19/2014	Spring	48.78333333	9.324074074	0	uwharrie	peidmont
4/20/2014	Spring	48.11666667	8.953703704	182	uwharrie	peidmont
4/21/2014	Spring	49.01666667	9.453703704	166	uwharrie	peidmont
4/22/2014	Spring	56.025	13.34722222	495	uwharrie	peidmont
4/23/2014	Spring	45.775	7.652777778	102	uwharrie	peidmont
4/24/2014	Spring	49.64166667	9.800925926	184	uwharrie	peidmont
4/25/2014	Spring	56.36666667	13.53703704	414	uwharrie	peidmont
4/26/2014	Spring	55.725	13.18055556	267	uwharrie	peidmont
4/27/2014	Spring	62.70833333	17.06018519	312	uwharrie	peidmont
4/28/2014	Spring	57.55833333	14.19907407	606	uwharrie	peidmont
4/29/2014	Spring	63.475	17.48611111	548	uwharrie	peidmont
4/30/2014	Spring	68.74545455	20.41414141	894	uwharrie	peidmont

5/1/2014	Spring	55.72727273	13.18181818	459	uwharrie	peidmont
5/2/2014	Spring	54.19090909	12.32828283	101	uwharrie	peidmont
5/3/2014	Spring	51.81818182	11.01010101	183	uwharrie	peidmont
5/4/2014	Spring	60.85454545	16.03030303	581	uwharrie	peidmont
5/5/2014	Spring	59.2	15.11111111	323	uwharrie	peidmont
5/6/2014	Spring	60.81818182	16.01010101	138	uwharrie	peidmont
5/7/2014	Spring	61.17272727	16.20707071	130	uwharrie	peidmont
5/8/2014	Spring	61.8	16.55555556	90	uwharrie	peidmont
5/9/2014	Spring	68.01818182	20.01010101	169	uwharrie	peidmont
5/10/2014	Spring	60.90909091	16.06060606	83	uwharrie	peidmont
5/11/2014	Spring	61.63636364	16.46464646	72	uwharrie	peidmont
5/12/2014	Spring	65.74545455	18.74747475	53	uwharrie	peidmont
5/13/2014	Spring	70.1	21.16666667	57	uwharrie	peidmont
5/14/2014	Spring	70.4	21.33333333	131	uwharrie	peidmont
5/15/2014	Spring	60.43636364	15.7979798	81	uwharrie	peidmont
5/16/2014	Spring	49.65454545	9.808080808	15	uwharrie	peidmont
5/17/2014	Spring	53.36363636	11.86868687	88	uwharrie	peidmont
5/18/2014	Spring	47.10909091	8.393939394	12	uwharrie	peidmont
5/19/2014	Spring	49.97272727	9.984848485	39	uwharrie	peidmont
5/20/2014	Spring	61.9	16.61111111	438	uwharrie	peidmont
5/23/2014	Spring	62.3	16.83333333	323	uwharrie	peidmont
5/24/2014	Spring	57.03636364	13.90909091	241	uwharrie	peidmont
5/25/2014	Spring	62.44545455	16.91414141	452	uwharrie	peidmont
5/26/2014	Spring	69.97272727	21.0959596	759	uwharrie	peidmont
5/27/2014	Spring	67.00909091	19.44949495	1015	uwharrie	peidmont
5/28/2014	Spring	68	20	827	uwharrie	peidmont
5/29/2014	Spring	68.64545455	20.35858586	642	uwharrie	peidmont
5/30/2014	Spring	67.78181818	19.87878788	1087	uwharrie	peidmont
5/31/2014	Spring	61.07272727	16.15151515	1227	uwharrie	peidmont
