# Supplementary material for: Winter Activity of Coastal Plain Populations of Bat Species Affected by White-Nose Syndrome and Wind Energy Facilities
Source: PLoS One. 2016 Nov 16;11(11):e0166512. doi: 10.1371/journal.pone.0166512 (PMC5112809; doi:10.1371/journal.pone.0166512)
Supplement: S5 Table — (PDF) [file pone.0166512.s005.pdf]

date	temp	celsius	site	region
12/1/12	44.5214286	6.95634921	Greensboro	Piedmont
12/2/12	55.0071429	12.781746	Greensboro	Piedmont
12/3/12	55.4266667	13.0148148	Greensboro	Piedmont
12/4/12	60.1933333	15.662963	Greensboro	Piedmont
12/5/12	46.7866667	8.21481482	Greensboro	Piedmont
12/6/12	44.9333333	7.18518519	Greensboro	Piedmont
12/7/12	47.9333333	8.85185185	Greensboro	Piedmont
12/8/12	54.88	12.7111111	Greensboro	Piedmont
12/9/12	59.92	15.5111111	Greensboro	Piedmont
12/10/12	59.5933333	15.3296296	Greensboro	Piedmont
12/11/12	42.5866667	5.88148148	Greensboro	Piedmont
12/12/12	41.48	5.26666667	Greensboro	Piedmont
12/13/12	33.8066667	1.0037037	Greensboro	Piedmont
12/14/12	37.5933333	3.10740741	Greensboro	Piedmont
12/15/12	49.2533333	9.58518519	Greensboro	Piedmont
12/16/12	53.32	11.8444444	Greensboro	Piedmont
12/17/12	55.0066667	12.7814815	Greensboro	Piedmont
12/18/12	42.6133333	5.8962963	Greensboro	Piedmont
12/19/12	44.3	6.83333333	Greensboro	Piedmont
12/20/12	48.5066667	9.17037037	Greensboro	Piedmont
12/21/12	36.3933333	2.44074074	Greensboro	Piedmont
12/22/12	35.2866667	1.82592593	Greensboro	Piedmont
12/23/12	44.72	7.06666667	Greensboro	Piedmont
12/24/12	44.6866667	7.04814815	Greensboro	Piedmont
12/25/12	45.22	7.34444444	Greensboro	Piedmont
12/26/12	37.1466667	2.85925926	Greensboro	Piedmont
12/27/12	33.3266667	0.73703704	Greensboro	Piedmont
12/28/12	36.5666667	2.53703704	Greensboro	Piedmont
12/29/12	37.96875	3.31597222	Greensboro	Piedmont
12/30/12	32.05	0.02777778	Greensboro	Piedmont
12/31/12	45.7375	7.63194444	Greensboro	Piedmont
1/1/13	46.28125	7.93402778	Greensboro	Piedmont
1/2/13	39.81875	4.34375	Greensboro	Piedmont
1/3/13	32.80625	0.44791667	Greensboro	Piedmont
1/4/13	35.275	1.81944444	Greensboro	Piedmont
1/5/13	41.16875	5.09375	Greensboro	Piedmont
1/6/13	41.25	5.13888889	Greensboro	Piedmont
1/7/13	33.90625	1.05902778	Greensboro	Piedmont
1/8/13	43.6875	6.49305556	Greensboro	Piedmont
1/9/13	53.16875	11.7604167	Greensboro	Piedmont
1/10/13	48.025	8.90277778	Greensboro	Piedmont

1/11/13	47.28125	8.48958333	Greensboro	Piedmont
1/12/13	56.49375	13.6076389	Greensboro	Piedmont
1/13/13	63.3125	17.3958333	Greensboro	Piedmont
1/14/13	52.6133333	11.4518519	Greensboro	Piedmont
1/15/13	40.1133333	4.50740741	Greensboro	Piedmont
1/16/13	49.1866667	9.54814815	Greensboro	Piedmont
1/17/13	33.0714286	0.5952381	Greensboro	Piedmont
1/18/13	29.2428571	-1.531746	Greensboro	Piedmont
1/19/13	40.1357143	4.51984127	Greensboro	Piedmont
1/20/13	38.7785714	3.76587302	Greensboro	Piedmont
1/21/13	38.8	3.77777778	Greensboro	Piedmont
1/22/13	24.3571429	-4.2460317	Greensboro	Piedmont
1/23/13	31.9285714	-0.0396825	Greensboro	Piedmont
1/24/13	23.2357143	-4.8690476	Greensboro	Piedmont
1/25/13	25.3	-3.7222222	Greensboro	Piedmont
1/26/13	29.3142857	-1.4920635	Greensboro	Piedmont
1/27/13	34.7214286	1.51190476	Greensboro	Piedmont
1/28/13	46.7071429	8.17063492	Greensboro	Piedmont
1/29/13	63.2428571	17.3571429	Greensboro	Piedmont
1/30/13	56.8071429	13.781746	Greensboro	Piedmont
1/31/13	37.5071429	3.05952381	Greensboro	Piedmont
2/1/13	24.2642857	-4.297619	Greensboro	Piedmont
2/2/13	37.0928571	2.82936508	Greensboro	Piedmont
2/3/13	34.9428571	1.63492064	Greensboro	Piedmont
2/4/13	40.5642857	4.75793651	Greensboro	Piedmont
2/5/13	41.0928571	5.0515873	Greensboro	Piedmont
2/6/13	41.2857143	5.15873016	Greensboro	Piedmont
2/7/13	37.1214286	2.8452381	Greensboro	Piedmont
2/8/13	39.2714286	4.03968254	Greensboro	Piedmont
2/9/13	33.4428571	0.8015873	Greensboro	Piedmont
2/10/13	45.3714286	7.42857143	Greensboro	Piedmont
2/11/13	51.6285714	10.9047619	Greensboro	Piedmont
2/12/13	47.15	8.41666667	Greensboro	Piedmont
2/13/13	38.1857143	3.43650794	Greensboro	Piedmont
2/14/13	38.1071429	3.39285714	Greensboro	Piedmont
2/15/13	43.8857143	6.6031746	Greensboro	Piedmont
2/16/13	29.0857143	-1.6190476	Greensboro	Piedmont
2/17/13	23.95	-4.4722222	Greensboro	Piedmont
2/18/13	39.7071429	4.28174603	Greensboro	Piedmont
2/19/13	38.2214286	3.45634921	Greensboro	Piedmont
2/20/13	35.7285714	2.07142857	Greensboro	Piedmont
2/21/13	39.7928571	4.32936508	Greensboro	Piedmont

2/22/13	34.4071429	1.33730159	Greensboro	Piedmont
2/23/13	42.3642857	5.75793651	Greensboro	Piedmont
2/24/13	42.8285714	6.01587302	Greensboro	Piedmont
2/25/13	34.5785714	1.43253968	Greensboro	Piedmont
2/26/13	39.85	4.36111111	Greensboro	Piedmont
2/27/13	43.7	6.5	Greensboro	Piedmont
2/28/13	39.2857143	4.04761905	Greensboro	Piedmont
12/1/13	38.4533333	3.58518519	Greensboro	Piedmont
12/2/13	44.3733333	6.87407407	Greensboro	Piedmont
12/3/13	50.16	10.0888889	Greensboro	Piedmont
12/4/13	57.9266667	14.4037037	Greensboro	Piedmont
12/5/13	64.8466667	18.2481482	Greensboro	Piedmont
12/7/13	41.54	5.3	Greensboro	Piedmont
12/8/13	32.5133333	0.28518519	Greensboro	Piedmont
12/9/13	43.1066667	6.17037037	Greensboro	Piedmont
12/10/13	33.2133333	0.67407407	Greensboro	Piedmont
12/11/13	37.6666667	3.14814815	Greensboro	Piedmont
12/12/13	31.34	-0.3666667	Greensboro	Piedmont
12/13/13	39.5933333	4.21851852	Greensboro	Piedmont
12/14/13	37.08	2.82222222	Greensboro	Piedmont
12/15/13	35.5066667	1.94814815	Greensboro	Piedmont
12/16/13	43.3133333	6.28518519	Greensboro	Piedmont
12/17/13	45.82	7.67777778	Greensboro	Piedmont
12/18/13	35.6	2	Greensboro	Piedmont
12/19/13	48.7266667	9.29259259	Greensboro	Piedmont
12/20/13	55.12	12.8444444	Greensboro	Piedmont
12/22/13	62.7866667	17.1037037	Greensboro	Piedmont
12/23/13	43.8266667	6.57037037	Greensboro	Piedmont
12/24/13	29.1733333	-1.5703704	Greensboro	Piedmont
12/25/13	27.0733333	-2.737037	Greensboro	Piedmont
12/26/13	36.3866667	2.43703704	Greensboro	Piedmont
12/27/13	33.4466667	0.8037037	Greensboro	Piedmont
12/28/13	45.0733333	7.26296296	Greensboro	Piedmont
12/29/13	44.91875	7.17708333	Greensboro	Piedmont
12/30/13	40.6375	4.79861111	Greensboro	Piedmont
12/31/13	36.9375	2.74305556	Greensboro	Piedmont
1/1/14	38.41875	3.56597222	Greensboro	Piedmont
1/2/14	34.1875	1.21527778	Greensboro	Piedmont
1/3/14	21.3625	-5.9097222	Greensboro	Piedmont
1/4/14	33.16875	0.64930556	Greensboro	Piedmont
1/5/14	41.6125	5.34027778	Greensboro	Piedmont
1/6/14	15.1	-9.3888889	Greensboro	Piedmont

1/7/14	17.36875	-8.1284722	Greensboro	Piedmont
1/8/14	31.01875	-0.5451389	Greensboro	Piedmont
1/9/14	41.43125	5.23958333	Greensboro	Piedmont
1/10/14	38.925	3.84722222	Greensboro	Piedmont
1/11/14	49.2875	9.60416667	Greensboro	Piedmont
1/12/14	42.95	6.08333333	Greensboro	Piedmont
1/13/14	48.05625	8.92013889	Greensboro	Piedmont
1/14/14	38.85625	3.80902778	Greensboro	Piedmont
1/15/14	37.58	3.1	Greensboro	Piedmont
1/16/14	34.3133333	1.28518519	Greensboro	Piedmont
1/17/14	38.7785714	3.76587302	Greensboro	Piedmont
1/18/14	31.3571429	-0.3571429	Greensboro	Piedmont
1/19/14	37.8285714	3.23809524	Greensboro	Piedmont
1/20/14	40.35	4.63888889	Greensboro	Piedmont
1/21/14	26.3357143	-3.1468254	Greensboro	Piedmont
1/22/14	21.3571429	-5.9126984	Greensboro	Piedmont
1/23/14	18.8857143	-7.2857143	Greensboro	Piedmont
1/24/14	19.4785714	-6.9563492	Greensboro	Piedmont
1/25/14	31.6	-0.2222222	Greensboro	Piedmont
1/26/14	44.1928571	6.77380952	Greensboro	Piedmont
1/27/14	32.5	0.27777778	Greensboro	Piedmont
1/28/14	17.5642857	-8.0198413	Greensboro	Piedmont
1/29/14	13.6928571	-10.170635	Greensboro	Piedmont
1/30/14	21.45	-5.8611111	Greensboro	Piedmont
1/31/14	33.2785714	0.71031746	Greensboro	Piedmont
2/1/14	46.8857143	8.26984127	Greensboro	Piedmont
2/2/14	54.1428571	12.3015873	Greensboro	Piedmont
2/3/14	38.7	3.72222222	Greensboro	Piedmont
2/4/14	36.9	2.72222222	Greensboro	Piedmont
2/5/14	37.7714286	3.20634921	Greensboro	Piedmont
2/6/14	35.85	2.13888889	Greensboro	Piedmont
2/7/14	36.8857143	2.71428571	Greensboro	Piedmont
2/8/14	38.0714286	3.37301587	Greensboro	Piedmont
2/9/14	42.5571429	5.86507937	Greensboro	Piedmont
2/10/14	32.2214286	0.12301587	Greensboro	Piedmont
2/11/14	25.6714286	-3.515873	Greensboro	Piedmont
2/12/14	24.8	-4	Greensboro	Piedmont
2/13/14	33.9928571	1.10714286	Greensboro	Piedmont
2/14/14	41.2785714	5.15476191	Greensboro	Piedmont
2/15/14	30.75	-0.6944444	Greensboro	Piedmont
2/16/14	37.0571429	2.80952381	Greensboro	Piedmont
2/17/14	37.1785714	2.87698413	Greensboro	Piedmont

2/18/14	48.0357143	8.90873016	Greensboro	Piedmont
2/19/14	54.5285714	12.515873	Greensboro	Piedmont
2/20/14	61.1285714	16.1825397	Greensboro	Piedmont
2/21/14	42.0571429	5.58730159	Greensboro	Piedmont
2/22/14	45.6142857	7.56349206	Greensboro	Piedmont
2/23/14	50.0785714	10.0436508	Greensboro	Piedmont
2/24/14	40.7285714	4.84920635	Greensboro	Piedmont
2/25/14	39.2142857	4.00793651	Greensboro	Piedmont
2/26/14	30.1428571	-1.031746	Greensboro	Piedmont
2/27/14	33.9285714	1.07142857	Greensboro	Piedmont
2/28/14	30.9785714	-0.5674603	Greensboro	Piedmont
12/1/12	42.25	5.69444444	Lenoir	Coast
12/2/12	53.4642857	11.9246032	Lenoir	Coast
12/3/12	53.9571429	12.1984127	Lenoir	Coast
12/4/12	59.8333333	15.462963	Lenoir	Coast
12/5/12	50.8866667	10.4925926	Lenoir	Coast
12/6/12	45.8333333	7.68518519	Lenoir	Coast
12/7/12	60.5333333	15.8518519	Lenoir	Coast
12/8/12	53.46	11.9222222	Lenoir	Coast
12/9/12	64.2066667	17.8925926	Lenoir	Coast
12/10/12	64.2866667	17.937037	Lenoir	Coast
12/11/12	50.7533333	10.4185185	Lenoir	Coast
12/12/12	40.86	4.92222222	Lenoir	Coast
12/13/12	40.58	4.76666667	Lenoir	Coast
12/14/12	36.3133333	2.3962963	Lenoir	Coast
12/15/12	50.08	10.0444444	Lenoir	Coast
12/16/12	57.72	14.2888889	Lenoir	Coast
12/17/12	58.1133333	14.5074074	Lenoir	Coast
12/18/12	45	7.22222222	Lenoir	Coast
12/19/12	44.4933333	6.94074074	Lenoir	Coast
12/20/12	57.78	14.3222222	Lenoir	Coast
12/21/12	39.1533333	3.97407407	Lenoir	Coast
12/22/12	35.5333333	1.96296296	Lenoir	Coast
12/23/12	44.8733333	7.15185185	Lenoir	Coast
12/24/12	48.52	9.17777778	Lenoir	Coast
12/25/12	48.5933333	9.21851852	Lenoir	Coast
12/26/12	49.9533333	9.97407407	Lenoir	Coast
12/27/12	36.5866667	2.54814815	Lenoir	Coast
12/28/12	41.24	5.13333333	Lenoir	Coast
12/29/12	42.12	5.62222222	Lenoir	Coast
12/30/12	31.5133333	-0.2703704	Lenoir	Coast
12/31/12	47.0666667	8.37037037	Lenoir	Coast

1/1/13	51.54	10.8555556	Lenoir	Coast
1/2/13	40.4933333	4.71851852	Lenoir	Coast
1/3/13	33.3666667	0.75925926	Lenoir	Coast
1/4/13	37.2666667	2.92592593	Lenoir	Coast
1/5/13	41.7266667	5.4037037	Lenoir	Coast
1/6/13	40.46875	4.70486111	Lenoir	Coast
1/7/13	34.5133333	1.3962963	Lenoir	Coast
1/8/13	43.1066667	6.17037037	Lenoir	Coast
1/9/13	52.1266667	11.1814815	Lenoir	Coast
1/10/13	47.56	8.64444444	Lenoir	Coast
1/11/13	59.4733333	15.262963	Lenoir	Coast
1/12/13	59.7866667	15.437037	Lenoir	Coast
1/13/13	61.9533333	16.6407407	Lenoir	Coast
1/14/13	62.6266667	17.0148148	Lenoir	Coast
1/15/13	53.2133333	11.7851852	Lenoir	Coast
1/16/13	52.0133333	11.1185185	Lenoir	Coast
1/17/13	39.46	4.14444444	Lenoir	Coast
1/18/13	32.7866667	0.43703704	Lenoir	Coast
1/19/13	45.4066667	7.44814815	Lenoir	Coast
1/20/13	40.68	4.82222222	Lenoir	Coast
1/21/13	46.0133333	7.78518519	Lenoir	Coast
1/22/13	26.88	-2.8444444	Lenoir	Coast
1/23/13	33.9466667	1.08148148	Lenoir	Coast
1/24/13	23.65	-4.6388889	Lenoir	Coast
1/25/13	27.8714286	-2.2936508	Lenoir	Coast
1/26/13	28.95	-1.6944444	Lenoir	Coast
1/27/13	30	-1.1111111	Lenoir	Coast
1/28/13	49.7785714	9.87698413	Lenoir	Coast
1/29/13	60.6214286	15.9007937	Lenoir	Coast
1/30/13	63.2142857	17.3412698	Lenoir	Coast
1/31/13	41.4214286	5.23412698	Lenoir	Coast
2/1/13	26.5785714	-3.0119048	Lenoir	Coast
2/2/13	39.9428571	4.41269841	Lenoir	Coast
2/3/13	32.9666667	0.53703704	Lenoir	Coast
2/4/13	41.4142857	5.23015873	Lenoir	Coast
2/5/13	41.8428571	5.46825397	Lenoir	Coast
2/6/13	42.75	5.97222222	Lenoir	Coast
2/7/13	48.0428571	8.91269841	Lenoir	Coast
2/8/13	43.8571429	6.58730159	Lenoir	Coast
2/9/13	32.2571429	0.14285714	Lenoir	Coast
2/10/13	50.2214286	10.1230159	Lenoir	Coast
2/11/13	60.15	15.6388889	Lenoir	Coast

2/12/13	50.2928571	10.1626984	Lenoir	Coast
2/13/13	47.6785714	8.71031746	Lenoir	Coast
2/14/13	37.3857143	2.99206349	Lenoir	Coast
2/15/13	43.6142857	6.45238095	Lenoir	Coast
2/16/13	32.1714286	0.0952381	Lenoir	Coast
2/17/13	26.1142857	-3.2698413	Lenoir	Coast
2/18/13	38.1214286	3.40079365	Lenoir	Coast
2/19/13	46.2571429	7.92063492	Lenoir	Coast
2/20/13	35.9214286	2.17857143	Lenoir	Coast
2/21/13	40.5357143	4.74206349	Lenoir	Coast
2/22/13	41.2357143	5.13095238	Lenoir	Coast
2/23/13	45.25	7.36111111	Lenoir	Coast
2/24/13	42.7428571	5.96825397	Lenoir	Coast
2/25/13	39.25	4.02777778	Lenoir	Coast
2/26/13	57.2571429	14.031746	Lenoir	Coast
2/27/13	46.6642857	8.1468254	Lenoir	Coast
2/28/13	40.3928571	4.66269841	Lenoir	Coast
12/1/13	42.2466667	5.69259259	Lenoir	Coast
12/2/13	40.0533333	4.47407407	Lenoir	Coast
12/3/13	52.5866667	11.437037	Lenoir	Coast
12/4/13	57.8066667	14.337037	Lenoir	Coast
12/7/13	43.1266667	6.18148148	Lenoir	Coast
12/8/13	38.0466667	3.35925926	Lenoir	Coast
12/9/13	52.2333333	11.2407407	Lenoir	Coast
12/10/13	35.16	1.75555556	Lenoir	Coast
12/11/13	35.56	1.97777778	Lenoir	Coast
12/12/13	29.64	-1.3111111	Lenoir	Coast
12/13/13	38.6733333	3.70740741	Lenoir	Coast
12/14/13	56.76	13.7555556	Lenoir	Coast
12/15/13	40.5	4.72222222	Lenoir	Coast
12/16/13	41.3733333	5.20740741	Lenoir	Coast
12/17/13	49.06	9.47777778	Lenoir	Coast
12/18/13	34.42	1.34444444	Lenoir	Coast
12/19/13	45.3933333	7.44074074	Lenoir	Coast
12/20/13	56.7133333	13.7296296	Lenoir	Coast
12/23/13	54.18	12.3222222	Lenoir	Coast
12/24/13	35.6666667	2.03703704	Lenoir	Coast
12/25/13	34.2	1.22222222	Lenoir	Coast
12/26/13	34.6733333	1.48518519	Lenoir	Coast
12/27/13	33.3866667	0.77037037	Lenoir	Coast
12/28/13	49.5466667	9.74814815	Lenoir	Coast
12/29/13	51.5	10.8333333	Lenoir	Coast

12/30/13	46.1133333	7.84074074	Lenoir	Coast
12/31/13	38.52	3.62222222	Lenoir	Coast
1/1/14	46.1133333	7.84074074	Lenoir	Coast
1/2/14	45.6533333	7.58518519	Lenoir	Coast
1/3/14	26.6933333	-2.9481481	Lenoir	Coast
1/4/14	39.1533333	3.97407407	Lenoir	Coast
1/5/14	63.0666667	17.2592593	Lenoir	Coast
1/6/14	29.3	-1.5	Lenoir	Coast
1/7/14	21	-6.1111111	Lenoir	Coast
1/8/14	28.1333333	-2.1481481	Lenoir	Coast
1/9/14	43.88	6.6	Lenoir	Coast
1/10/14	63.26	17.3666667	Lenoir	Coast
1/11/14	55.8	13.2222222	Lenoir	Coast
1/12/14	41.4533333	5.25185185	Lenoir	Coast
1/13/14	55.24	12.9111111	Lenoir	Coast
1/14/14	41.3733333	5.20740741	Lenoir	Coast
1/15/14	44.1	6.72222222	Lenoir	Coast
1/16/14	32.9066667	0.5037037	Lenoir	Coast
1/17/14	47.1466667	8.41481482	Lenoir	Coast
1/18/14	31.18	-0.4555556	Lenoir	Coast
1/19/14	40.5733333	4.76296296	Lenoir	Coast
1/20/14	45.92	7.73333333	Lenoir	Coast
1/21/14	33.2466667	0.69259259	Lenoir	Coast
1/22/14	22.3133333	-5.3814815	Lenoir	Coast
1/23/14	26.9733333	-2.7925926	Lenoir	Coast
1/24/14	19.6	-6.8888889	Lenoir	Coast
1/25/14	36.95	2.75	Lenoir	Coast
1/26/14	43.2428571	6.24603175	Lenoir	Coast
1/27/14	42.6142857	5.8968254	Lenoir	Coast
1/28/14	18.9571429	-7.2460317	Lenoir	Coast
1/29/14	16.5857143	-8.5634921	Lenoir	Coast
1/30/14	33.1357143	0.63095238	Lenoir	Coast
1/31/14	29.45	-1.4166667	Lenoir	Coast
2/1/14	42.6928571	5.94047619	Lenoir	Coast
2/2/14	48.95	9.41666667	Lenoir	Coast
2/3/14	42.4571429	5.80952381	Lenoir	Coast
2/4/14	40.7642857	4.86904762	Lenoir	Coast
2/5/14	46.4357143	8.01984127	Lenoir	Coast
2/6/14	34.8428571	1.57936508	Lenoir	Coast
2/7/14	33.7071429	0.9484127	Lenoir	Coast
2/8/14	31.4	-0.3333333	Lenoir	Coast
2/9/14	43.3428571	6.3015873	Lenoir	Coast

2/10/14	35.7071429	2.05952381	Lenoir	Coast
2/12/14	35.9076923	2.17094017	Lenoir	Coast
2/13/14	36.4357143	2.46428571	Lenoir	Coast
2/14/14	44.9	7.16666667	Lenoir	Coast
2/15/14	34.5357143	1.40873016	Lenoir	Coast
2/16/14	34.3285714	1.29365079	Lenoir	Coast
2/17/14	37.0928571	2.82936508	Lenoir	Coast
2/18/14	47.3142857	8.50793651	Lenoir	Coast
2/19/14	56.2083333	13.4490741	Lenoir	Coast
2/21/14	49.9642857	9.98015873	Lenoir	Coast
2/22/14	40.1142857	4.50793651	Lenoir	Coast
2/23/14	51.95	11.0833333	Lenoir	Coast
2/24/14	38.2076923	3.44871795	Lenoir	Coast
2/25/14	39.4071429	4.11507937	Lenoir	Coast
2/26/14	31.9785714	-0.0119048	Lenoir	Coast
2/27/14	35.4928571	1.94047619	Lenoir	Coast
2/28/14	32.9357143	0.51984127	Lenoir	Coast
12/1/12	42.4928571	5.82936508	North River	Coast
12/2/12	56.3142857	13.5079365	North River	Coast
12/3/12	54.7333333	12.6296296	North River	Coast
12/4/12	61.54	16.4111111	North River	Coast
12/5/12	48.5466667	9.19259259	North River	Coast
12/6/12	44.8066667	7.11481482	North River	Coast
12/7/12	63.1933333	17.3296296	North River	Coast
12/8/12	54.5	12.5	North River	Coast
12/9/12	64.2133333	17.8962963	North River	Coast
12/10/12	66.06	18.9222222	North River	Coast
12/11/12	52.36	11.3111111	North River	Coast
12/12/12	48.4933333	9.16296296	North River	Coast
12/13/12	44.8266667	7.12592593	North River	Coast
12/14/12	39.4466667	4.13703704	North River	Coast
12/15/12	52.48	11.3777778	North River	Coast
12/16/12	59.1133333	15.062963	North River	Coast
12/17/12	60.36	15.7555556	North River	Coast
12/18/12	48.4666667	9.14814815	North River	Coast
12/19/12	48.1733333	8.98518518	North River	Coast
12/20/12	60.7	15.9444444	North River	Coast
12/21/12	40.6066667	4.78148148	North River	Coast
12/22/12	38.7066667	3.72592593	North River	Coast
12/23/12	47.22	8.45555556	North River	Coast
12/24/12	51.5866667	10.8814815	North River	Coast
12/25/12	49.4666667	9.70370371	North River	Coast

12/26/12	53.7266667	12.0703704	North River	Coast
12/27/12	39.38	4.1	North River	Coast
12/28/12	44.2333333	6.79629629	North River	Coast
12/29/12	42.58	5.87777778	North River	Coast
12/30/12	34.9333333	1.62962963	North River	Coast
12/31/12	48.2133333	9.00740741	North River	Coast
1/1/13	47.96	8.86666667	North River	Coast
1/2/13	41.06	5.03333333	North River	Coast
1/3/13	34.12	1.17777778	North River	Coast
1/4/13	39.02	3.9	North River	Coast
1/5/13	43.0133333	6.11851852	North River	Coast
1/6/13	43.7133333	6.50740741	North River	Coast
1/7/13	36.5866667	2.54814815	North River	Coast
1/8/13	44.94	7.18888889	North River	Coast
1/9/13	55.6	13.1111111	North River	Coast
1/10/13	42.9866667	6.10370371	North River	Coast
1/11/13	61.1533333	16.1962963	North River	Coast
1/12/13	55.2	12.8888889	North River	Coast
1/13/13	60.36	15.7555556	North River	Coast
1/14/13	58.4533333	14.6962963	North River	Coast
1/15/13	52.1066667	11.1703704	North River	Coast
1/16/13	53.1533333	11.7518519	North River	Coast
1/17/13	41.38	5.21111111	North River	Coast
1/18/13	33.8466667	1.02592593	North River	Coast
1/19/13	48.2866667	9.04814815	North River	Coast
1/20/13	46.6	8.11111111	North River	Coast
1/21/13	45.0533333	7.25185185	North River	Coast
1/22/13	27.6933333	-2.3925926	North River	Coast
1/23/13	34.4666667	1.37037037	North River	Coast
1/24/13	25.48	-3.6222222	North River	Coast
1/25/13	29.8266667	-1.2074074	North River	Coast
1/26/13	31.3214286	-0.3769841	North River	Coast
1/27/13	33.5857143	0.88095238	North River	Coast
1/28/13	52.1928571	11.218254	North River	Coast
1/29/13	60.3142857	15.7301587	North River	Coast
1/30/13	66.7428571	19.3015873	North River	Coast
1/31/13	42.2214286	5.67857143	North River	Coast
2/1/13	27.2285714	-2.6507937	North River	Coast
2/2/13	40.05	4.47222222	North River	Coast
2/3/13	36.6428571	2.57936508	North River	Coast
2/4/13	43.6785714	6.48809524	North River	Coast
2/5/13	42.9214286	6.06746032	North River	Coast

2/6/13	43.9214286	6.62301587	North River	Coast
2/7/13	51.6928571	10.9404762	North River	Coast
2/8/13	43.9714286	6.65079365	North River	Coast
2/9/13	38.85	3.80555556	North River	Coast
2/10/13	49.4357143	9.68650794	North River	Coast
2/11/13	61.9142857	16.6190476	North River	Coast
2/12/13	51.9642857	11.0912698	North River	Coast
2/13/13	48.1071429	8.9484127	North River	Coast
2/14/13	39.4285714	4.12698413	North River	Coast
2/15/13	45.5071429	7.50396826	North River	Coast
2/16/13	34.5642857	1.42460317	North River	Coast
2/17/13	26.1714286	-3.2380952	North River	Coast
2/18/13	40.7071429	4.83730159	North River	Coast
2/19/13	48.7928571	9.32936508	North River	Coast
2/20/13	37.7142857	3.17460317	North River	Coast
2/21/13	40.5285714	4.73809524	North River	Coast
2/22/13	49.5071429	9.72619048	North River	Coast
2/23/13	47.1	8.38888889	North River	Coast
2/24/13	41.4785714	5.26587302	North River	Coast
2/25/13	41	5	North River	Coast
2/26/13	60.2	15.6666667	North River	Coast
2/27/13	49.3571429	9.64285714	North River	Coast
2/28/13	40.6357143	4.79761905	North River	Coast
12/1/13	35.8133333	2.11851852	North River	Coast
12/2/13	35.6466667	2.02592593	North River	Coast
12/3/13	48.5666667	9.2037037	North River	Coast
12/4/13	55.26	12.9222222	North River	Coast
12/5/13	65.0533333	18.362963	North River	Coast
12/6/13	61.46	16.3666667	North River	Coast
12/7/13	41.1733333	5.0962963	North River	Coast
12/11/13	33.5	0.83333333	North River	Coast
12/12/13	27.2066667	-2.662963	North River	Coast
12/14/13	54.8466667	12.6925926	North River	Coast
12/15/13	38.1466667	3.41481482	North River	Coast
12/16/13	37.7133333	3.17407407	North River	Coast
12/17/13	43.1533333	6.1962963	North River	Coast
12/18/13	30.6	-0.7777778	North River	Coast
12/19/13	44.5733333	6.98518519	North River	Coast
12/20/13	56.2933333	13.4962963	North River	Coast
12/21/13	64.9733333	18.3185185	North River	Coast
12/22/13	69.22	20.6777778	North River	Coast
12/23/13	48.8333333	9.35185185	North River	Coast

12/24/13	31.8666667	-0.0740741	North River	Coast
12/25/13	29.1733333	-1.5703704	North River	Coast
12/26/13	30.06	-1.0777778	North River	Coast
12/27/13	29.9266667	-1.1518519	North River	Coast
12/28/13	45.82	7.67777778	North River	Coast
12/29/13	47.92	8.84444444	North River	Coast
12/30/13	43.4466667	6.35925926	North River	Coast
12/31/13	33.7466667	0.97037037	North River	Coast
1/1/14	43.0266667	6.12592593	North River	Coast
1/2/14	42.2733333	5.70740741	North River	Coast
1/3/14	23.1933333	-4.8925926	North River	Coast
1/4/14	36.2733333	2.37407407	North River	Coast
1/5/14	61.7266667	16.5148148	North River	Coast
1/6/14	26.8133333	-2.8814815	North River	Coast
1/7/14	16.62	-8.5444444	North River	Coast
1/8/14	24.26	-4.3	North River	Coast
1/9/14	36.34	2.41111111	North River	Coast
1/10/14	58.9933333	14.9962963	North River	Coast
1/11/14	55.0333333	12.7962963	North River	Coast
1/12/14	38.34	3.52222222	North River	Coast
1/13/14	55.46	13.0333333	North River	Coast
1/14/14	42.3133333	5.72962963	North River	Coast
1/15/14	42.7533333	5.97407407	North River	Coast
1/16/14	31.18	-0.4555556	North River	Coast
1/17/14	45.6466667	7.58148148	North River	Coast
1/18/14	29.5933333	-1.337037	North River	Coast
1/19/14	38.2	3.44444444	North River	Coast
1/20/14	42.0533333	5.58518519	North River	Coast
1/21/14	26.54	-3.0333333	North River	Coast
1/22/14	11.3266667	-11.485185	North River	Coast
1/23/14	24.66	-4.0777778	North River	Coast
1/24/14	17.5533333	-8.0259259	North River	Coast
1/25/14	33.2333333	0.68518519	North River	Coast
1/26/14	41.18	5.1	North River	Coast
1/27/14	35.7266667	2.07037037	North River	Coast
1/28/14	19.5	-6.9444444	North River	Coast
1/29/14	14.1466667	-9.9185185	North River	Coast
1/30/14	32.78	0.43333333	North River	Coast
2/1/14	42.1214286	5.62301587	North River	Coast
2/2/14	47.7785714	8.76587302	North River	Coast
2/3/14	37.8928571	3.27380952	North River	Coast
2/4/14	40.5571429	4.75396825	North River	Coast

2/6/14	31.6642857	-0.1865079	North River	Coast
2/7/14	35.35	1.86111111	North River	Coast
2/9/14	39.9142857	4.3968254	North River	Coast
2/10/14	31.4928571	-0.281746	North River	Coast
2/11/14	28.3285714	-2.0396825	North River	Coast
2/12/14	46.2714286	7.92857143	North River	Coast
2/13/14	36.8285714	2.68253968	North River	Coast
2/15/14	32.4	0.22222222	North River	Coast
2/16/14	36.7	2.61111111	North River	Coast
2/17/14	36.5	2.5	North River	Coast
2/18/14	46.5785714	8.09920635	North River	Coast
2/22/14	42	5.55555556	North River	Coast
2/23/14	50.0642857	10.0357143	North River	Coast
2/24/14	41.4214286	5.23412698	North River	Coast
2/25/14	37.5428571	3.07936508	North River	Coast
2/26/14	32.8642857	0.48015873	North River	Coast
2/27/14	35.2928571	1.82936508	North River	Coast
2/28/14	31.35	-0.3611111	North River	Coast
12/1/12	40.6533333	4.80740741	Parker Tract	Coast
12/2/12	53.6466667	12.0259259	Parker Tract	Coast
12/3/12	51.26	10.7	Parker Tract	Coast
12/4/12	59.02	15.0111111	Parker Tract	Coast
12/5/12	47.2733333	8.48518519	Parker Tract	Coast
12/6/12	42.5	5.83333333	Parker Tract	Coast
12/7/12	61.6533333	16.4740741	Parker Tract	Coast
12/8/12	53.8466667	12.137037	Parker Tract	Coast
12/9/12	61.9066667	16.6148148	Parker Tract	Coast
12/10/12	62.4866667	16.937037	Parker Tract	Coast
12/11/12	49.6933333	9.82962963	Parker Tract	Coast
12/12/12	43.9066667	6.61481482	Parker Tract	Coast
12/13/12	41.54	5.3	Parker Tract	Coast
12/14/12	35.88	2.15555556	Parker Tract	Coast
12/15/12	49.5733333	9.76296296	Parker Tract	Coast
12/16/12	57.64	14.2444444	Parker Tract	Coast
12/17/12	57.8466667	14.3592593	Parker Tract	Coast
12/18/12	45.6266667	7.57037037	Parker Tract	Coast
12/19/12	43.88	6.6	Parker Tract	Coast
12/20/12	58.5533333	14.7518519	Parker Tract	Coast
12/21/12	39.4466667	4.13703704	Parker Tract	Coast
12/22/12	33.0666667	0.59259259	Parker Tract	Coast
12/23/12	42.5733333	5.87407407	Parker Tract	Coast
12/24/12	49.1133333	9.50740741	Parker Tract	Coast

12/25/12	47.82	8.78888889	Parker Tract	Coast
12/26/12	51.4533333	10.8074074	Parker Tract	Coast
12/27/12	37.4333333	3.01851852	Parker Tract	Coast
12/28/12	41.7533333	5.41851852	Parker Tract	Coast
12/29/12	41.7733333	5.42962963	Parker Tract	Coast
12/30/12	31.9066667	-0.0518519	Parker Tract	Coast
12/31/12	46.3866667	7.99259259	Parker Tract	Coast
1/1/13	46.3933333	7.9962963	Parker Tract	Coast
1/2/13	39.7733333	4.31851852	Parker Tract	Coast
1/3/13	32.22	0.12222222	Parker Tract	Coast
1/4/13	36.4	2.44444444	Parker Tract	Coast
1/5/13	39.8066667	4.33703704	Parker Tract	Coast
1/6/13	41.0266667	5.01481482	Parker Tract	Coast
1/7/13	33.6333333	0.90740741	Parker Tract	Coast
1/8/13	42.78	5.98888889	Parker Tract	Coast
1/9/13	52.4733333	11.3740741	Parker Tract	Coast
1/10/13	43.6066667	6.44814815	Parker Tract	Coast
1/11/13	60.1666667	15.6481482	Parker Tract	Coast
1/12/13	57.0666667	13.9259259	Parker Tract	Coast
1/13/13	58.7533333	14.862963	Parker Tract	Coast
1/14/13	57.9866667	14.437037	Parker Tract	Coast
1/15/13	49.9333333	9.96296296	Parker Tract	Coast
1/16/13	51.94	11.0777778	Parker Tract	Coast
1/17/13	39.0266667	3.9037037	Parker Tract	Coast
1/18/13	30.74	-0.7	Parker Tract	Coast
1/19/13	44.5133333	6.95185185	Parker Tract	Coast
1/20/13	40.2733333	4.5962963	Parker Tract	Coast
1/21/13	44.6466667	7.02592593	Parker Tract	Coast
1/22/13	25.8666667	-3.4074074	Parker Tract	Coast
1/23/13	31.58	-0.2333333	Parker Tract	Coast
1/24/13	24.9866667	-3.8962963	Parker Tract	Coast
1/25/13	29.7133333	-1.2703704	Parker Tract	Coast
1/26/13	29.6266667	-1.3185185	Parker Tract	Coast
1/27/13	30.7466667	-0.6962963	Parker Tract	Coast
1/28/13	50.7466667	10.4148148	Parker Tract	Coast
1/29/13	58.6357143	14.7976191	Parker Tract	Coast
1/30/13	64.3428571	17.968254	Parker Tract	Coast
1/31/13	39.7428571	4.3015873	Parker Tract	Coast
2/1/13	25.7642857	-3.4642857	Parker Tract	Coast
2/2/13	36.8928571	2.71825397	Parker Tract	Coast
2/3/13	36.2714286	2.37301587	Parker Tract	Coast
2/4/13	40.1285714	4.51587302	Parker Tract	Coast

2/5/13	39.3571429	4.08730159	Parker Tract	Coast
2/6/13	41.6857143	5.38095238	Parker Tract	Coast
2/7/13	48.8571429	9.36507937	Parker Tract	Coast
2/8/13	42.4928571	5.82936508	Parker Tract	Coast
2/9/13	31.55	-0.25	Parker Tract	Coast
2/10/13	47.4642857	8.59126984	Parker Tract	Coast
2/11/13	60.4214286	15.7896825	Parker Tract	Coast
2/12/13	49.4285714	9.68253968	Parker Tract	Coast
2/13/13	46.7857143	8.21428571	Parker Tract	Coast
2/14/13	36.4928571	2.49603175	Parker Tract	Coast
2/15/13	40.6785714	4.82142857	Parker Tract	Coast
2/16/13	32.3285714	0.18253968	Parker Tract	Coast
2/17/13	25.6214286	-3.5436508	Parker Tract	Coast
2/18/13	34.4642857	1.36904762	Parker Tract	Coast
2/19/13	47.6571429	8.6984127	Parker Tract	Coast
2/20/13	36.0214286	2.23412698	Parker Tract	Coast
2/21/13	38.7928571	3.77380952	Parker Tract	Coast
2/22/13	44.75	7.08333333	Parker Tract	Coast
2/23/13	45.0071429	7.22619048	Parker Tract	Coast
2/24/13	40.2142857	4.56349206	Parker Tract	Coast
2/25/13	39.2071429	4.00396825	Parker Tract	Coast
2/26/13	58.05	14.4722222	Parker Tract	Coast
2/27/13	45.2857143	7.38095238	Parker Tract	Coast
2/28/13	45.2857143	7.38095238	Parker Tract	Coast
12/1/13	39.9133333	4.3962963	Parker Tract	Coast
12/2/13	37.5933333	3.10740741	Parker Tract	Coast
12/3/13	50.2	10.1111111	Parker Tract	Coast
12/4/13	55.86	13.2555556	Parker Tract	Coast
12/5/13	65.92	18.8444444	Parker Tract	Coast
12/6/13	67.9666667	19.9814815	Parker Tract	Coast
12/7/13	42.4933333	5.82962963	Parker Tract	Coast
12/8/13	39.9666667	4.42592593	Parker Tract	Coast
12/9/13	49.8133333	9.8962963	Parker Tract	Coast
12/10/13	33.86	1.03333333	Parker Tract	Coast
12/11/13	33.32	0.73333333	Parker Tract	Coast
12/12/13	28.8266667	-1.762963	Parker Tract	Coast
12/13/13	34.8466667	1.58148148	Parker Tract	Coast
12/15/13	38.5133333	3.61851852	Parker Tract	Coast
12/16/13	39.0466667	3.91481482	Parker Tract	Coast
12/17/13	47.0266667	8.34814815	Parker Tract	Coast
12/18/13	32.18	0.1	Parker Tract	Coast
12/19/13	45.2	7.33333333	Parker Tract	Coast

12/20/13	58.26	14.5888889	Parker Tract	Coast
12/23/13	53.3466667	11.8592593	Parker Tract	Coast
12/24/13	34.98	1.65555556	Parker Tract	Coast
12/25/13	34.22	1.23333333	Parker Tract	Coast
12/26/13	33.1066667	0.61481482	Parker Tract	Coast
12/27/13	31.76	-0.1333333	Parker Tract	Coast
12/28/13	47.5533333	8.64074074	Parker Tract	Coast
12/29/13	52.18	11.2111111	Parker Tract	Coast
12/30/13	45.5	7.5	Parker Tract	Coast
12/31/13	36.04	2.24444444	Parker Tract	Coast
1/1/14	45.3733333	7.42962963	Parker Tract	Coast
1/2/14	45.52	7.51111111	Parker Tract	Coast
1/3/14	25.9866667	-3.3407407	Parker Tract	Coast
1/4/14	38.64	3.68888889	Parker Tract	Coast
1/5/14	62.96	17.2	Parker Tract	Coast
1/6/14	28.71875	-1.8229167	Parker Tract	Coast
1/7/14	17.88	-7.8444444	Parker Tract	Coast
1/8/14	26.26	-3.1888889	Parker Tract	Coast
1/9/14	41.5866667	5.32592593	Parker Tract	Coast
1/10/14	60.72	15.9555556	Parker Tract	Coast
1/11/14	56.4466667	13.5814815	Parker Tract	Coast
1/12/14	40.74	4.85555556	Parker Tract	Coast
1/13/14	55.3533333	12.9740741	Parker Tract	Coast
1/14/14	43.6066667	6.44814815	Parker Tract	Coast
1/15/14	43.8666667	6.59259259	Parker Tract	Coast
1/16/14	30.9933333	-0.5592593	Parker Tract	Coast
1/17/14	46.22	7.9	Parker Tract	Coast
1/18/14	29.76	-1.2444444	Parker Tract	Coast
1/19/14	39.4533333	4.14074074	Parker Tract	Coast
1/20/14	35.46	1.92222222	Parker Tract	Coast
1/21/14	29.4733333	-1.4037037	Parker Tract	Coast
1/22/14	10.58	-11.9	Parker Tract	Coast
1/23/14	25.0733333	-3.8481481	Parker Tract	Coast
1/24/14	12.28	-10.955556	Parker Tract	Coast
1/25/14	35.2142857	1.78571429	Parker Tract	Coast
1/26/14	40.6214286	4.78968254	Parker Tract	Coast
1/27/14	35.8428571	2.13492064	Parker Tract	Coast
1/28/14	19.6142857	-6.8809524	Parker Tract	Coast
1/29/14	15.3928571	-9.2261905	Parker Tract	Coast
1/30/14	32.35	0.19444444	Parker Tract	Coast
1/31/14	22.5	-5.2777778	Parker Tract	Coast
2/1/14	41.9642857	5.53571429	Parker Tract	Coast

2/2/14	41.4285714	5.23809524	Parker Tract	Coast
2/3/14	38.9857143	3.88095238	Parker Tract	Coast
2/4/14	39.8642857	4.36904762	Parker Tract	Coast
2/5/14	43.8071429	6.55952381	Parker Tract	Coast
2/6/14	32.0785714	0.04365079	Parker Tract	Coast
2/7/14	30.9928571	-0.5595238	Parker Tract	Coast
2/8/14	31.4857143	-0.2857143	Parker Tract	Coast
2/9/14	38.2857143	3.49206349	Parker Tract	Coast
2/10/14	32.7571429	0.42063492	Parker Tract	Coast
2/11/14	28.3857143	-2.0079365	Parker Tract	Coast
2/12/14	46.9571429	8.30952381	Parker Tract	Coast
2/13/14	36.5571429	2.53174603	Parker Tract	Coast
2/14/14	39.9785714	4.43253968	Parker Tract	Coast
2/15/14	31.3642857	-0.3531746	Parker Tract	Coast
2/16/14	33.0285714	0.57142857	Parker Tract	Coast
2/17/14	34.5285714	1.40476191	Parker Tract	Coast
2/18/14	37.1428571	2.85714286	Parker Tract	Coast
2/19/14	52.3357143	11.2976191	Parker Tract	Coast
2/20/14	62.6142857	17.0079365	Parker Tract	Coast
2/21/14	49.15	9.52777778	Parker Tract	Coast
2/22/14	35.1285714	1.73809524	Parker Tract	Coast
2/23/14	44.0642857	6.70238095	Parker Tract	Coast
2/24/14	37.3214286	2.95634921	Parker Tract	Coast
2/25/14	38.8	3.77777778	Parker Tract	Coast
2/26/14	32.2785714	0.15476191	Parker Tract	Coast
2/27/14	33.2071429	0.67063492	Parker Tract	Coast
2/28/14	32.4571429	0.25396825	Parker Tract	Coast
12/1/12	45.7571429	7.64285714	South River	Coast
12/2/12	54.5	12.5	South River	Coast
12/3/12	54.5	12.5	South River	Coast
12/4/12	58.04	14.4666667	South River	Coast
12/6/12	46.3266667	7.95925926	South River	Coast
12/7/12	58.46	14.7	South River	Coast
12/8/12	54.86	12.7	South River	Coast
12/9/12	63.8733333	17.7074074	South River	Coast
12/10/12	64.6666667	18.1481482	South River	Coast
12/11/12	54.44	12.4666667	South River	Coast
12/12/12	40.6933333	4.82962963	South River	Coast
12/13/12	39.8	4.33333333	South River	Coast
12/14/12	37.2533333	2.91851852	South River	Coast
12/15/12	51.7066667	10.9481482	South River	Coast
12/16/12	57.92	14.4	South River	Coast

12/17/12	57.4466667	14.137037	South River	Coast
12/18/12	46.24	7.91111111	South River	Coast
12/19/12	44.8266667	7.12592593	South River	Coast
12/20/12	57.2866667	14.0481482	South River	Coast
12/21/12	38.3733333	3.54074074	South River	Coast
12/22/12	35.38	1.87777778	South River	Coast
12/23/12	46.5	8.05555556	South River	Coast
12/24/12	49.2933333	9.60740741	South River	Coast
12/25/12	51	10.5555556	South River	Coast
12/26/12	47.5133333	8.61851852	South River	Coast
12/27/12	38.5133333	3.61851852	South River	Coast
12/28/12	43.12	6.17777778	South River	Coast
12/30/12	31.06	-0.5222222	South River	Coast
12/31/12	51.1466667	10.637037	South River	Coast
1/1/13	52.46	11.3666667	South River	Coast
1/2/13	43.0933333	6.16296296	South River	Coast
1/4/13	35.2071429	1.78174603	South River	Coast
1/5/13	41.52	5.28888889	South River	Coast
1/6/13	42.5533333	5.86296296	South River	Coast
1/7/13	39.4866667	4.15925926	South River	Coast
1/8/13	46.66	8.14444444	South River	Coast
1/9/13	56.5285714	13.6269841	South River	Coast
1/10/13	54.5066667	12.5037037	South River	Coast
1/11/13	62.0866667	16.7148148	South River	Coast
1/12/13	61.7533333	16.5296296	South River	Coast
1/13/13	61.36	16.3111111	South River	Coast
1/14/13	63.3428571	17.4126984	South River	Coast
1/15/13	61.25	16.25	South River	Coast
1/16/13	57.0785714	13.9325397	South River	Coast
1/17/13	42.1846154	5.65811966	South River	Coast
1/18/13	33.3071429	0.72619048	South River	Coast
1/19/13	45.0285714	7.23809524	South River	Coast
1/20/13	41.8142857	5.45238095	South River	Coast
1/21/13	48.2428571	9.02380952	South River	Coast
1/22/13	29.4142857	-1.4365079	South River	Coast
1/23/13	38.1428571	3.41269841	South River	Coast
1/24/13	25.2461539	-3.7521368	South River	Coast
1/25/13	29.9571429	-1.1349206	South River	Coast
1/26/13	29.1	-1.6111111	South River	Coast
1/27/13	34.4714286	1.37301587	South River	Coast
1/28/13	50.6642857	10.3690476	South River	Coast
1/29/13	62.45	16.9166667	South River	Coast

1/30/13	62.2142857	16.7857143	South River	Coast
1/31/13	43.6928571	6.49603174	South River	Coast
2/1/13	28.3071429	-2.0515873	South River	Coast
2/2/13	39.7785714	4.32142857	South River	Coast
2/3/13	38.35	3.52777778	South River	Coast
2/4/13	41.8714286	5.48412698	South River	Coast
2/5/13	49.1	9.5	South River	Coast
2/6/13	45.4714286	7.48412698	South River	Coast
2/7/13	49.9214286	9.95634921	South River	Coast
2/8/13	46.3	7.94444444	South River	Coast
2/9/13	35.1357143	1.74206349	South River	Coast
2/10/13	53.5642857	11.9801587	South River	Coast
2/11/13	59.9714286	15.5396825	South River	Coast
2/12/13	51.1076923	10.6153846	South River	Coast
2/13/13	48.4642857	9.14682539	South River	Coast
2/14/13	38.3	3.5	South River	Coast
2/15/13	44.0214286	6.67857143	South River	Coast
2/16/13	33.2357143	0.68650794	South River	Coast
2/17/13	28.8153846	-1.7692308	South River	Coast
2/18/13	37.1642857	2.86904762	South River	Coast
2/19/13	45.8857143	7.71428572	South River	Coast
2/20/13	37.2071429	2.89285714	South River	Coast
2/21/13	44.3285714	6.84920635	South River	Coast
2/22/13	40.5	4.72222222	South River	Coast
2/23/13	45.5928571	7.5515873	South River	Coast
2/24/13	44.0857143	6.71428572	South River	Coast
2/25/13	41.6214286	5.34523809	South River	Coast
2/26/13	55.1928571	12.8849206	South River	Coast
2/27/13	47.0071429	8.33730159	South River	Coast
2/28/13	38.9785714	3.87698413	South River	Coast
12/1/13	47.52	8.62222222	South River	Coast
12/2/13	42.72	5.95555556	South River	Coast
12/3/13	53.9533333	12.1962963	South River	Coast
12/4/13	57.06	13.9222222	South River	Coast
12/6/13	68.7733333	20.4296296	South River	Coast
12/7/13	42.82	6.01111111	South River	Coast
12/8/13	38.44	3.57777778	South River	Coast
12/9/13	56.4357143	13.5753968	South River	Coast
12/10/13	37.28	2.93333333	South River	Coast
12/11/13	36.58	2.54444444	South River	Coast
12/12/13	31.3066667	-0.3851852	South River	Coast
12/13/13	41.3333333	5.18518519	South River	Coast

12/14/13	58.7733333	14.8740741	South River	Coast
12/16/13	41.92	5.51111111	South River	Coast
12/17/13	50.9933333	10.5518519	South River	Coast
12/18/13	34.62	1.45555556	South River	Coast
12/19/13	45.7866667	7.65925926	South River	Coast
12/20/13	56.52	13.6222222	South River	Coast
12/21/13	67.3666667	19.6481482	South River	Coast
12/23/13	54.1933333	12.3296296	South River	Coast
12/24/13	35.1066667	1.72592593	South River	Coast
12/25/13	34.66	1.47777778	South River	Coast
12/26/13	36.6666667	2.59259259	South River	Coast
12/27/13	34.4	1.33333333	South River	Coast
12/28/13	52.9466667	11.637037	South River	Coast
12/29/13	49.8333333	9.90740741	South River	Coast
12/30/13	47.18	8.43333333	South River	Coast
12/31/13	38.08	3.37777778	South River	Coast
1/1/14	44.7066667	7.05925926	South River	Coast
1/2/14	44.9333333	7.18518519	South River	Coast
1/3/14	26.2733333	-3.1814815	South River	Coast
1/4/14	39.8933333	4.38518519	South River	Coast
1/6/14	28.22	-2.1	South River	Coast
1/7/14	20.6466667	-6.3074074	South River	Coast
1/8/14	29.48	-1.4	South River	Coast
1/9/14	47.94	8.85555556	South River	Coast
1/10/14	65.9266667	18.8481482	South River	Coast
1/11/14	54.3733333	12.4296296	South River	Coast
1/12/14	40.1533333	4.52962963	South River	Coast
1/13/14	54.1266667	12.2925926	South River	Coast
1/14/14	41.1857143	5.1031746	South River	Coast
1/15/14	42.7214286	5.95634921	South River	Coast
1/16/14	32.8	0.44444444	South River	Coast
1/17/14	45.8214286	7.67857143	South River	Coast
1/18/14	32.65	0.36111111	South River	Coast
1/20/14	46.0571429	7.80952381	South River	Coast
1/21/14	36.5357143	2.51984127	South River	Coast
1/22/14	24.2785714	-4.2896825	South River	Coast
1/23/14	26.7357143	-2.9246032	South River	Coast
1/24/14	17.7142857	-7.9365079	South River	Coast
1/26/14	43.6285714	6.46031746	South River	Coast
1/27/14	44.5357143	6.96428571	South River	Coast
1/28/14	21.9071429	-5.6071429	South River	Coast
1/29/14	20.1357143	-6.5912698	South River	Coast

1/30/14	33.5571429	0.86507937	South River	Coast
1/31/14	31.9928571	-0.0039683	South River	Coast
2/1/14	41.9	5.5	South River	Coast
2/2/14	53.3285714	11.8492064	South River	Coast
2/3/14	45.6357143	7.57539683	South River	Coast
2/4/14	40.8666667	4.92592593	South River	Coast
2/5/14	47.9857143	8.88095238	South River	Coast
2/6/14	35.6714286	2.03968254	South River	Coast
2/7/14	37.0285714	2.79365079	South River	Coast
2/8/14	34.4142857	1.34126984	South River	Coast
2/9/14	45.6785714	7.59920635	South River	Coast
2/10/14	37.7071429	3.17063492	South River	Coast
2/11/14	28.1428571	-2.1428571	South River	Coast
2/13/14	35.1357143	1.74206349	South River	Coast
2/14/14	46.3714286	7.98412698	South River	Coast
2/15/14	36.0142857	2.23015873	South River	Coast
2/16/14	38.4714286	3.5952381	South River	Coast
2/17/14	40.7071429	4.83730159	South River	Coast
2/18/14	50.3285714	10.1825397	South River	Coast
2/19/14	60.8142857	16.0079365	South River	Coast
2/20/14	65.3571429	18.531746	South River	Coast
2/21/14	52.25	11.25	South River	Coast
2/22/14	45.5214286	7.51190476	South River	Coast
2/23/14	54.3785714	12.4325397	South River	Coast
2/24/14	45.3846154	7.43589744	South River	Coast
2/25/14	45.5785714	7.54365079	South River	Coast
2/26/14	33.6285714	0.90476191	South River	Coast
2/27/14	38.2285714	3.46031746	South River	Coast
12/1/12	41.56	5.31111111	Uwharrie	Piedmont
12/2/12	49.7333333	9.85185185	Uwharrie	Piedmont
12/3/12	48.7866667	9.32592593	Uwharrie	Piedmont
12/4/12	56.7	13.7222222	Uwharrie	Piedmont
12/5/12	46.0533333	7.80740741	Uwharrie	Piedmont
12/6/12	47.28	8.48888889	Uwharrie	Piedmont
12/7/12	49.4133333	9.67407407	Uwharrie	Piedmont
12/8/12	50.9866667	10.5481482	Uwharrie	Piedmont
12/9/12	59.88	15.4888889	Uwharrie	Piedmont
12/10/12	61.92	16.6222222	Uwharrie	Piedmont
12/11/12	46.8266667	8.23703704	Uwharrie	Piedmont
12/12/12	38.5466667	3.63703704	Uwharrie	Piedmont
12/13/12	31.5	-0.2777778	Uwharrie	Piedmont
12/14/12	33.22	0.67777778	Uwharrie	Piedmont

12/15/12	49.2933333	9.60740741	Uwharrie	Piedmont
12/16/12	54	12.2222222	Uwharrie	Piedmont
12/17/12	56.1866667	13.437037	Uwharrie	Piedmont
12/18/12	40.0266667	4.45925926	Uwharrie	Piedmont
12/19/12	40.1933333	4.55185185	Uwharrie	Piedmont
12/20/12	50.0266667	10.0148148	Uwharrie	Piedmont
12/21/12	34.9	1.61111111	Uwharrie	Piedmont
12/22/12	29.0733333	-1.6259259	Uwharrie	Piedmont
12/23/12	41.8733333	5.48518519	Uwharrie	Piedmont
12/24/12	47.0466667	8.35925926	Uwharrie	Piedmont
12/25/12	47.1466667	8.41481482	Uwharrie	Piedmont
12/26/12	39.44	4.13333333	Uwharrie	Piedmont
12/27/12	32.3466667	0.19259259	Uwharrie	Piedmont
12/28/12	38.3266667	3.51481482	Uwharrie	Piedmont
12/29/12	37.92	3.28888889	Uwharrie	Piedmont
12/30/12	27.6266667	-2.4296296	Uwharrie	Piedmont
12/31/12	44.7133333	7.06296296	Uwharrie	Piedmont
1/1/13	49.52	9.73333333	Uwharrie	Piedmont
1/2/13	41.8666667	5.48148148	Uwharrie	Piedmont
1/3/13	30.7466667	-0.6962963	Uwharrie	Piedmont
1/4/13	32.1066667	0.05925926	Uwharrie	Piedmont
1/5/13	40	4.44444444	Uwharrie	Piedmont
1/6/13	36.28	2.37777778	Uwharrie	Piedmont
1/7/13	32.72	0.4	Uwharrie	Piedmont
1/8/13	45.4066667	7.44814815	Uwharrie	Piedmont
1/9/13	51.46	10.8111111	Uwharrie	Piedmont
1/10/13	49.82	9.9	Uwharrie	Piedmont
1/11/13	49.7066667	9.83703704	Uwharrie	Piedmont
1/12/13	56.2133333	13.4518519	Uwharrie	Piedmont
1/13/13	62.0266667	16.6814815	Uwharrie	Piedmont
1/14/13	59.2266667	15.1259259	Uwharrie	Piedmont
1/15/13	45.7714286	7.65079365	Uwharrie	Piedmont
1/16/13	50.9928571	10.5515873	Uwharrie	Piedmont
1/17/13	35.5285714	1.96031746	Uwharrie	Piedmont
1/18/13	26.3642857	-3.1309524	Uwharrie	Piedmont
1/19/13	34.8785714	1.59920635	Uwharrie	Piedmont
1/20/13	34.3642857	1.31349206	Uwharrie	Piedmont
1/21/13	40.9357143	4.96428571	Uwharrie	Piedmont
1/22/13	24.5857143	-4.1190476	Uwharrie	Piedmont
1/23/13	30.0785714	-1.0674603	Uwharrie	Piedmont
1/24/13	22.9857143	-5.0079365	Uwharrie	Piedmont
1/25/13	23.7928571	-4.5595238	Uwharrie	Piedmont

1/26/13	27.65	-2.4166667	Uwharrie	Piedmont
1/27/13	34.3857143	1.32539683	Uwharrie	Piedmont
1/28/13	44.2714286	6.81746032	Uwharrie	Piedmont
1/29/13	62.5571429	16.9761905	Uwharrie	Piedmont
1/30/13	57.3285714	14.0714286	Uwharrie	Piedmont
1/31/13	37.8857143	3.26984127	Uwharrie	Piedmont
2/1/13	22.8857143	-5.0634921	Uwharrie	Piedmont
2/2/13	39.8785714	4.37698413	Uwharrie	Piedmont
2/3/13	31.6071429	-0.218254	Uwharrie	Piedmont
2/4/13	37.8571429	3.25396825	Uwharrie	Piedmont
2/5/13	37.0428571	2.8015873	Uwharrie	Piedmont
2/6/13	39.2285714	4.01587302	Uwharrie	Piedmont
2/7/13	41.4714286	5.26190476	Uwharrie	Piedmont
2/8/13	40.2928571	4.60714286	Uwharrie	Piedmont
2/9/13	29.8142857	-1.2142857	Uwharrie	Piedmont
2/10/13	51.1285714	10.6269841	Uwharrie	Piedmont
2/11/13	53.2928571	11.8293651	Uwharrie	Piedmont
2/12/13	47.2214286	8.45634921	Uwharrie	Piedmont
2/13/13	39.1071429	3.9484127	Uwharrie	Piedmont
2/14/13	32.2857143	0.15873016	Uwharrie	Piedmont
2/15/13	41.1214286	5.06746032	Uwharrie	Piedmont
2/16/13	29.2285714	-1.5396825	Uwharrie	Piedmont
2/17/13	22.9214286	-5.0436508	Uwharrie	Piedmont
2/18/13	37	2.77777778	Uwharrie	Piedmont
2/19/13	36.6571429	2.58730159	Uwharrie	Piedmont
2/20/13	31.5714286	-0.2380952	Uwharrie	Piedmont
2/21/13	41.3214286	5.17857143	Uwharrie	Piedmont
2/22/13	35.9571429	2.1984127	Uwharrie	Piedmont
2/23/13	43.1071429	6.17063492	Uwharrie	Piedmont
2/24/13	38.2142857	3.45238095	Uwharrie	Piedmont
2/25/13	36	2.22222222	Uwharrie	Piedmont
2/26/13	45.1642857	7.31349206	Uwharrie	Piedmont
2/27/13	38.2714286	3.48412698	Uwharrie	Piedmont
2/28/13	33.1571429	0.64285714	Uwharrie	Piedmont
12/1/13	42.6	5.88888889	Uwharrie	Piedmont
12/2/13	41.8533333	5.47407407	Uwharrie	Piedmont
12/3/13	51.9933333	11.1074074	Uwharrie	Piedmont
12/4/13	57.46	14.1444444	Uwharrie	Piedmont
12/5/13	64.0333333	17.7962963	Uwharrie	Piedmont
12/6/13	65.7666667	18.7592593	Uwharrie	Piedmont
12/7/13	43.7933333	6.55185185	Uwharrie	Piedmont
12/8/13	34.22	1.23333333	Uwharrie	Piedmont

12/9/13	44.8933333	7.16296296	Uwharrie	Piedmont
12/10/13	30.4666667	-0.8518519	Uwharrie	Piedmont
12/11/13	28.1	-2.1666667	Uwharrie	Piedmont
12/12/13	25.0666667	-3.8518519	Uwharrie	Piedmont
12/13/13	35.2866667	1.82592593	Uwharrie	Piedmont
12/14/13	41.7866667	5.43703704	Uwharrie	Piedmont
12/15/13	33.38	0.76666667	Uwharrie	Piedmont
12/16/13	36.8666667	2.7037037	Uwharrie	Piedmont
12/17/13	44.7666667	7.09259259	Uwharrie	Piedmont
12/18/13	29.2733333	-1.5148148	Uwharrie	Piedmont
12/19/13	43.66	6.47777778	Uwharrie	Piedmont
12/20/13	49.6333333	9.7962963	Uwharrie	Piedmont
12/21/13	68.2333333	20.1296296	Uwharrie	Piedmont
12/22/13	64.1266667	17.8481482	Uwharrie	Piedmont
12/23/13	47.2066667	8.44814815	Uwharrie	Piedmont
12/24/13	29.2133333	-1.5481481	Uwharrie	Piedmont
12/25/13	30.08	-1.0666667	Uwharrie	Piedmont
12/26/13	30.62	-0.7666667	Uwharrie	Piedmont
12/27/13	28.4133333	-1.9925926	Uwharrie	Piedmont
12/28/13	44.4333333	6.90740741	Uwharrie	Piedmont
12/29/13	38.48	3.6	Uwharrie	Piedmont
12/30/13	41.16	5.08888889	Uwharrie	Piedmont
12/31/13	31.9066667	-0.0518519	Uwharrie	Piedmont
1/1/14	37.1266667	2.84814815	Uwharrie	Piedmont
1/2/14	37.68	3.15555556	Uwharrie	Piedmont
1/3/14	20.2333333	-6.537037	Uwharrie	Piedmont
1/4/14	33.64	0.91111111	Uwharrie	Piedmont
1/5/14	47.4666667	8.59259259	Uwharrie	Piedmont
1/6/14	17.5133333	-8.0481481	Uwharrie	Piedmont
1/7/14	15.6466667	-9.0851852	Uwharrie	Piedmont
1/8/14	24.76	-4.0222222	Uwharrie	Piedmont
1/9/14	44.02	6.67777778	Uwharrie	Piedmont
1/10/14	44.1266667	6.73703704	Uwharrie	Piedmont
1/11/14	49.5933333	9.77407407	Uwharrie	Piedmont
1/12/14	34.5866667	1.43703704	Uwharrie	Piedmont
1/13/14	47.7733333	8.76296296	Uwharrie	Piedmont
1/14/14	37.6466667	3.13703704	Uwharrie	Piedmont
1/15/14	36.3571429	2.42063492	Uwharrie	Piedmont
1/16/14	30.3642857	-0.9087302	Uwharrie	Piedmont
1/17/14	38.4071429	3.55952381	Uwharrie	Piedmont
1/18/14	30.5428571	-0.8095238	Uwharrie	Piedmont
1/19/14	32.6285714	0.34920635	Uwharrie	Piedmont

1/20/14	34.5142857	1.3968254	Uwharrie	Piedmont
1/21/14	30.1357143	-1.0357143	Uwharrie	Piedmont
1/22/14	17.5642857	-8.0198413	Uwharrie	Piedmont
1/23/14	22.7071429	-5.1626984	Uwharrie	Piedmont
1/24/14	16.5769231	-8.5683761	Uwharrie	Piedmont
1/25/14	31.6642857	-0.1865079	Uwharrie	Piedmont
1/26/14	43.1785714	6.21031746	Uwharrie	Piedmont
1/27/14	37.4714286	3.03968254	Uwharrie	Piedmont
1/28/14	20.5142857	-6.3809524	Uwharrie	Piedmont
1/29/14	13.2214286	-10.43254	Uwharrie	Piedmont
1/30/14	16.7785714	-8.4563492	Uwharrie	Piedmont
1/31/14	27.8571429	-2.3015873	Uwharrie	Piedmont
2/1/14	43.1714286	6.20634921	Uwharrie	Piedmont
2/2/14	52.9642857	11.6468254	Uwharrie	Piedmont
2/3/14	42.2785714	5.71031746	Uwharrie	Piedmont
2/4/14	37.1285714	2.84920635	Uwharrie	Piedmont
2/5/14	39.8857143	4.38095238	Uwharrie	Piedmont
2/6/14	34.4214286	1.3452381	Uwharrie	Piedmont
2/7/14	33.9928571	1.10714286	Uwharrie	Piedmont
2/8/14	33.75	0.97222222	Uwharrie	Piedmont
2/9/14	41.3857143	5.21428571	Uwharrie	Piedmont
2/10/14	33.8714286	1.03968254	Uwharrie	Piedmont
2/11/14	27.3142857	-2.6031746	Uwharrie	Piedmont
2/12/14	26.3230769	-3.1538462	Uwharrie	Piedmont
2/13/14	31.7285714	-0.1507937	Uwharrie	Piedmont
2/14/14	39.4571429	4.14285714	Uwharrie	Piedmont
2/15/14	31.4071429	-0.3293651	Uwharrie	Piedmont
2/16/14	32.0571429	0.03174603	Uwharrie	Piedmont
2/17/14	36.0642857	2.25793651	Uwharrie	Piedmont
2/18/14	44.9285714	7.18253968	Uwharrie	Piedmont
2/19/14	54.1357143	12.2976191	Uwharrie	Piedmont
2/20/14	65.75	18.75	Uwharrie	Piedmont
2/21/14	38.7142857	3.73015873	Uwharrie	Piedmont
2/22/14	37.4071429	3.00396825	Uwharrie	Piedmont
2/23/14	47.6285714	8.68253968	Uwharrie	Piedmont
2/24/14	37.2	2.88888889	Uwharrie	Piedmont
2/25/14	40.3214286	4.62301587	Uwharrie	Piedmont
2/26/14	29.25	-1.5277778	Uwharrie	Piedmont
2/27/14	32.1571429	0.08730159	Uwharrie	Piedmont
2/28/14	34.6428571	1.46825397	Uwharrie	Piedmont
